# The Mechanism of Histone Ubiquitylation by the ASB9-CUL5 Ubiquitin Ligase

**DOI:** 10.1016/j.mcpro.2025.101471

**Published:** 2025-11-17

**Authors:** Calvin P. Lin, Nathan H. Lee, Francis X. Alipranti, Harry Li, Elizabeth A. Komives

**Affiliations:** Department of Chemistry and Biochemistry, University of California San Diego, La Jolla, California, USA

**Keywords:** histones, cullin-RING ligase, ubiquitylation, protein degradation, ankyrin, SOCS box substrate receptor

## Abstract

The E3 ligase substrate receptor ankyrin and suppressor of cytokine signaling box protein 9 (ASB9) was shown to bind over 10 different proteins including metabolic enzymes such as creatine kinase, filament proteins such as vimentin, and histones. In previous work, we characterized the ASB9-Cullin 5 E3 ligase (ASB9-CRL5) ubiquitylation of creatine kinase and showed that ubiquitylation required the ring-between-ring ligase, ARIH2. Here, we characterized the ASB9-CRL5 ubiquitylation of histones and show that histones histone 3 (H3) and histone 4 (H4) are polyubiquitylated by the ASB9-CRL5 whereas histones Histone 2A and Histone 2B are much poorer substrates. Many, but not all lysines in the histones are ubiquitylated suggesting some substrate specificity. Binding experiments show that the ligase-histone interaction is highly electrostatic and the neddylated ASB9-CRL5 binds with the highest affinity. Histones in nucleosomes or in complex with the chaperone Asf1, are not ubiquitylated. Only K48 and K63 polyubiquitin chains were observed, suggesting that the ubiquitylation probably drives histone degradation. The presence of ASB9 in specific cell types correlates with situations in which free histones H3 and H4 need to be degraded. In this work, we demonstrate that the ASB9-CRL5 is the ligase that facilitates degradation of histones H3 and H4. In addition, this work represents the first example of Cullin-5 mediated ubiquitylation that does not require a ring-between-ring “helper” ligase.

## Highlights

• Histones H3 and H4 are *bona fide* substrates for the ASB9-CRL.
• Histones H3 and H4 are preferentially polyubiquitylated by the ASB9-CRL.
• Polyubiquitylation of H3 and H4 by the ASB9-CRL does not require the RBR ARIH2.

## Graphical Abstract

**Figure:**
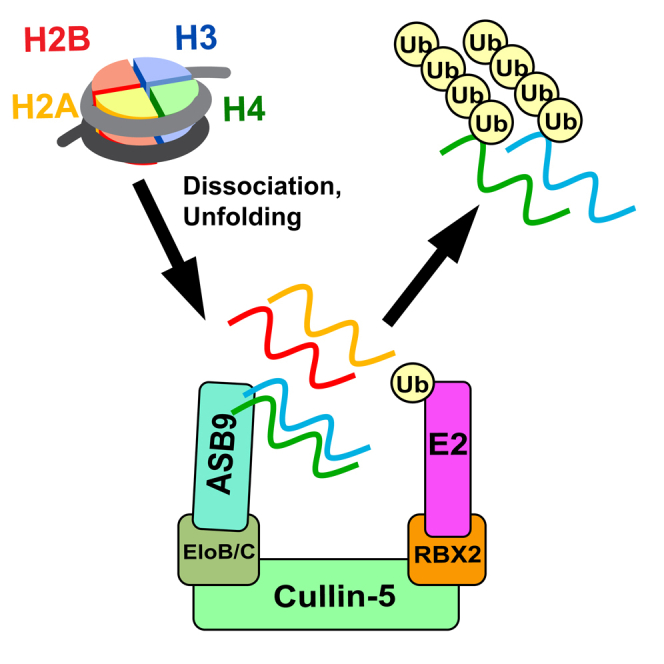

## In Brief

The ASB9-CUL5 Ubiquitin Ligase was identified as the E3 ligase that polyubiquitylates extranucleosomal histones H3 and H4 by a novel mechanism not requiring ARIH2. ASB9 is found mainly in testes, where degradation of free histones is linked to spermatoid maturation.

Ubiquitylation is a post-translational modification that regulates a variety of biological processes and is mediated through a multi-step enzymatic cascade (1). Most commonly, ubiquitin-conjugation mediates protein degradation required for regulating gene transcription, cell cycle progression, signaling cascades, viral immunity, and tumor suppression (2, 3, 4). The E1 ubiquitin (Ub) activating enzyme is first conjugated to Ub in an ATP-dependent manner. The Ub is then transferred to an E2 conjugating enzyme through a trans-thiolation reaction. In the third step, E3 ubiquitin ligases interact with Ub-conjugated E2s and substrate proteins to promote covalent Ub attachment to the substrate ϵ-amino group of lysine residues by either a direct or indirect mechanism. In the direct mechanism, E3s such as Homologous to the E6-AP Carboxyl Terminus E3 ligases or RING-between-RING (RBR) E3 ligases receive a Ub from an E2-Ub before ubiquitylating the substrate. In the more common indirect mechanism, E3s bind the E2-Ub and the Ub is transferred from the E2 to the substrate. E3s such as Cullin-RING ligases (CRLs) act as a scaffold to allow ubiquitin conjugation from E2s to substrates. The human genome is comprised of two E1s, 37 E2s, and over 600 E3s to ubiquitylate a wide variety of proteins (5). The diversity of E3s is needed to recognize and ubiquitylate the diversity of cellular proteins (6).

The Cullin-RING ligase family, the largest group of E3 ligases, consists of a complex of proteins including a substrate receptor, adapter protein(s), a Cullin, and a RING-Box protein (RBX) which binds an E2 carrying the thioester-linked Ub (7). Unique combinations of adapter proteins and substrate receptors allow CRLs to recruit and target thousands of substrates. Adapter protein(s) interact with the N-terminal region of Cullins and recruit specific classes of substrate receptors depending on the interaction motif they contain. For example, Cullin 5 (CUL5) bind Elongin B and Elongin C (ELOB/C) adapter proteins, which then bind different ankyrin and suppressor of cytokine signaling (SOCS)-box (ASB) substrate receptors containing a consensus SOCS-box binding sequence (4). A quantitative SILAC mass spectrometry study comprehensively identified proteins that bound to each of the 18 ASB proteins (8). We decided to focus on the structurally characterized ankyrin and SOCS box protein 9 (ASB9) (9, 10, 11), which is found in only three main organs, kidney, liver, and most abundantly testis (12). Creatine kinase B (CKB), a verified substrate of the ASB9-ELOB/C-CUL5-RBX2 (ASB9-CRL5), immunoprecipitated with ASB9 (12, 13, 14, 15, 16) in high abundance. Other interacting proteins varied in structure and spanned a variety of cellular functions such as metabolism (CKB, ENO2), membrane trafficking (RAB1A), and chromatin formation (Histone 2A (H2A)FZ, Histone 2B (H2B) FD, Histone 3 (H3).3A, H4/A, and H1F2) (8). Previous work from our lab developed a structural model of the ASB9-CRL5 with CKB bound from structures of CKB-ASB9-ELOB/C and CUL5-RBX2 (10) and showed that ubiquitylation of CKB requires the ring-between-ring ligase, ARIH2 and its obligate E2 enzyme, UBE2L3 (13). Cullin-mediated substrate ubiquitylation often requires CRLs to partner with a RING-between-RING E3 ARIH ligase family member (14), to form cooperative E3-E3 super-assemblies. One model suggests that certain folded Cullin substrates require an additional ARIH E3 ligase because the E2∼Ub located at the Cullin C-terminus is unable to reach the substrate near the Cullin N-terminus, while an ARIH-UBE2L3∼Ub E3 ligase is positioned closer to such substrates (15). Mechanistic studies have only characterized CRL5 substrates that require ARIH2, specifically CKB ubiquitylation by ASB9-CRL5 (16, 17) and APOBEC3G ubiquitylation by Vif-CBFβ-CRL5 (13, 16, 18). Here we focus on the mechanism of histone ubiquitylation by the ASB9-CRL5 and ask whether this reaction also requires ARIH2.

Histone accumulation (19, 20) and histone scarcity (21) have been shown to lead to genome instability and cell death. Thus, histone levels are tightly regulated through transcriptional, posttranscriptional, and posttranslational mechanisms including ubiquitin-mediated degradation. Histone ubiquitylation can occur in either DNA-bound or free histones that are not DNA-bound. CUL4 ligases have been shown to mediate histone mono- and poly-ubiquitylation in nucleosomes at sites of DNA damage (22) or chromatin remodeling (23) to release histones from DNA and recruit repair/remodeling proteins. Excess histones that accumulate in the cytosol have been shown to be ubiquitylated and degraded by HEL1 and HEL2 (24). Cullin-5 has been shown to localize to both the cytosol (14, 25) and the nucleus at sites of DNA damage (26) and transcriptional stalling (27).

Here we present *in vitro* studies of histone ubiquitylation with reconstituted ASB9-CRL5 to determine which histones are ubiquitylated and in what form (nucleosomal or free histones). We identify which lysines are modified (28). The results demonstrate that ASB9-CRL5 is a novel histone E3 ligase and presents a model in which ASB9-CRL5 poly-ubiquitylates free histones H3 and H4 for proteasomal degradation (12).

## Experimental Procedures

### Expression Vectors

Vectors pertaining to ASB9-CRL5 complex, ubiquitin, E1 activating enzyme, and E2 conjugating enzyme expression in *Escherichia coli* (*E. coli*) were obtained as previously described (10, 17). Vectors for expression of *Xenopus laevis* histones H2A, H2B, H3, and H4 in *E. Coli* were a gift from Shannon Lauberth. Site directed mutagenesis of the WT H4 vector was done using blunt-end cloning to generate the H4 V22C vector. pQTEV-ASF1A was a gift from Konrad Buessow (Addgene plasmid # 31591; http://n2t.net/addgene:31591; RRID:Addgene_31591) (29).

### E1/E2/E3 Expression

Expression of ASB9-CRL5 and ubiquitin machinery in *E. coli* was performed as previously described (10, 17) with some modifications. Co-expression cells containing plasmids for ASB9/ELOB/C or CUL5/RBX2/ELOB/C were made using sequential transformation. The vector containing ELOB/C (Cam^R^) was transformed into competent BL21(DE3) cells (Invitrogen) after which those cells were made competent again. ELOB/C in pACYC was selected for chloramphenicol (CAM) resistance. The vector for expression of ASB9 (Kan^R^) was transformed into ELOB/C-containing BL21 cells and plated on a kanamycin (Kan)-Cam LB agar plate for later culture inoculation and glycerol stock production. Similarly, vectors containing RBX2 (Amp^R^) and CUL5 (Kan^R^) were sequentially transformed into competent cells containing the ELOB/C vector (Cam^R^) to generate *E. coli* cells for co-expression of CUL5/RBX2/ELOB/C from cell plates or glycerol stocks. *E. coli* expression vectors containing RBX1 (Amp^R^) and CUL1 (Kan^R^) were sequentially transformed into BL21(DE3) cells for co-expression from KAN-AMP LB agar plates or from glycerol stocks. *E. coli* expression vectors containing Ub (Kan^R^) and UBE1 (Amp^R^) were sequentially transformed into BL21(DE3) cells for co-expression from KAN-AMP LB agar plates or from glycerol stocks. Vectors for expression of UBE2D2, UBE2L3, UBE2F, Ub, and neural precursor cell expressed, developmentally down-regulated (NEDD8) were transformed individually into BL21 cells and plated on KAN LB agar plates. Vectors for expression of NAE1/UBA3 and ARIH2 were transformed individually into BL21 cells and plated on AMP LB agar plates. Transformation of the NAE1/UBA3 vector into competent cells containing the NEDD8 vector was performed as mentioned above for co-expression of NAE1/UBA3 and NEDD8.

All non-histone proteins were expressed as follows. A 20 ml M9-ZN (1.5 × M9 salts, NZ-Amine media, 0.8% dextrose, 1 mM MgSO_4_, 0.2 mM CaCl_2_) overnight culture was inoculated with a single colony from the plate or from a glycerol stock. Then 1 L M9-ZN media was inoculated with the entire 20 ml starter culture and grown to OD_600_ = 0.8 at 37 °C. After placing the cultures on ice for 15 min, protein expression was induced by addition of IPTG to a final concentration of 0.5 mM, and the cultures were transferred to an 18 °C incubator for 20 h. RBX1, RBX2, and ARIH2 were expressed in media containing 200 μM ZnCl_2_ added just prior to induction.

### Histone Expression

Vectors containing *Xenopus laevis* H2A, H2B, H3, and H4 were individually transformed into BL21 Star(DE3)pLysS (Invitrogen) and plated on AMP-CAM LB agar plates. Single colonies were used to inoculate 20 ml LB cultures and grown overnight with shaking at 37 °C. The overnight cultures were then used to inoculate 1 L large cultures of 2xYT media and grown to OD_600_ = 1.0 at 37 °C. Protein expression was induced by addition of IPTG (final concentration 0.2 mM), and the cultures were grown for another 3 h. Cells were collected by centrifugation at 5500 *g* for 25 min and the collected pellet was frozen at −80 °C until use.

### Protein Purification of ASB9-CRL5 Complexes, Ubiquitin Machinery, and Asf1

Purification of ASB9-containing complexes; ASB9-ELOB/C, ASB9-ELOB/C-CUL5-RBX2, or ubiquitin machinery; UBE1, UBE2D2, UBE2L3, UBE2F, and ARIH2 was performed as previously described (10, 17) with some modifications. Cells were resuspended in a buffer supplemented with protease inhibitor cocktail (Sigma P2714) and 5 mM PMSF, sonicated, and clarified by centrifugation at 25,000 *g* for 35 min. The clarified lysate was incubated with 2 ml HisPur Ni-NTA Resin (Thermo Fisher Scientific 88,221) (pre-equilibrated in resuspension buffer) for 2 h at 4 °C with rocking. Ni-NTA beads were pelleted by centrifugation at 800 *g* for 5 min. The supernatant was discarded, and the Ni-NTA beads were poured into a glass Econo-Column Chromatography Column (Bio-Rad), where Ni-NTA beads were washed with 20 ml wash buffer (50 mM Tris-HCl pH 8.0, 400 mM NaCl, 30 mM imidazole pH 8.0, 2 mM β-mercaptoethanol, 5% glycerol) at 4 °C, and His-tagged protein was eluted from the Ni-NTA beads using 14 ml elution buffer (50 mM Tris-HCl pH 8.0, 250 mM NaCl, 250 mM imidazole pH 8.0, 2 mM β-mercaptoethanol, 5% glycerol) over 30 min at 4 °C. The eluate was dialyzed overnight (10 kDa cut-off) in dialysis buffer (20 mM Tris-HCl pH 8.0, 100 mM NaCl, 5% glycerol, 1 mM DTT. Samples were concentrated to 2 ml and purified using size-exclusion chromatography over a Superdex 200 16 × 600 column (Cytiva) in dialysis buffer. Peak fractions were combined and concentrated with Vivaspin 10 kDa MWCO concentrator (Sartorius) for use in experiments. Asf1 was purified in the same manner as described for the components of ASB9-containing complexes except without glycerol. The Asf1-H3-H4 complex was made by incubating the three proteins in a 2:1:1 ratio and purified by size-exclusion on a Superdex 200 Increase 10/300 column.

### Neddylation of CUL5

ASB9-ELOB/C-CUL5-RBX2 (ASB9-CRL5) was neddylated during its purification as previously described with some modifications (10, 17). Pellets of ASB9-ELOB/C (2/3 L cell pellet), ELOB/C-CUL5-RBX2 (0.5 L cell pellet), and the NAE1/UBA3∼NEDD8 (0.5 L cell pellet) were resuspended in 100 ml resuspension buffer with protease inhibitor cocktail (Sigma P2714), 5 mM PMSF, and 10 mM MgCl_2_ and 2 mM ATP at 4 °C and sonicated. Lysates were purified with 1 ml Ni-NTA resin as described above and dialyzed overnight. The dialyzed sample was brought to 5 mM MgCl_2_ and 2 mM ATP (Thermo Fisher Scientific BP413) by addition 1 M MgCl_2_ and a freshly made 100 mM ATP solution in dialysis buffer. 2 mg of previously purified ∼4 mg/ml UBE2F stocks in in 20 mM Tris pH 8.0, 250 mM NaCl, 50% glycerol, 1 mM DTT (the E2 for CUL5 neddylation) was added to the dialyzed sample and incubated at 4 °C for 2 h. The ∼8 ml sample was concentrated to ∼4 ml. The neddylated CUL5 complex was purified from NAE1/UBA3, UBE2F, and excess NEDD8 on a Superdex S200 16 × 600 column (Cytiva) in dialysis buffer using a 2-mL injection loop. The fractions were analyzed by SDS-PAGE and fractions containing pure NEDD8-ASB9-ELOB/C-CUL5-RBX2 complex were combined, concentrated, and used immediately.

### Histone Purification

Histone monomers were prepared using established protocols (30, 31). Histones were expressed in BL21(DE3) pLysS *E. coli* cells and purified from inclusion bodies by gel-filtration using a Sephacryl 200 column (GE Healthcare) under denaturing conditions, followed by dialysis in MQ-H_2_O containing 5 mM β-mercaptoethanol and lyophilization. Histones were resuspended in 0.5 M Na Acetate pH 5.2, 7 M urea, 200 mM NaCl, and 5 mM β-mercaptoethanol and further purified by ion-exchange chromatography using a 5-mL HiTrap SP HP column (Cytiva) followed by dialysis in MQ-H_2_O containing 5 mM β-mercaptoethanol, lyophilization, and storage at −80 °C.

### Histone Octamer Assembly and Purification

Histone octamers were prepared using established protocols (31, 32). Briefly, lyophilized histones were individually dissolved using 20 mM Tris-HCl pH 7.5, 7 M guanidine HCl, 5 mM β-mercaptoethanol to 2 mg/ml as determined by UV absorbance (ε_280_ = 44,700 M^−1^ cm^−1^). The four histone proteins were combined in equimolar amounts and adjusted to a final octamer concentration of 1 mg/ml. The solution was dialyzed against 10 mM Tris-HCl pH 7.5, 2 M NaCl, 1 mM DTT and octamers were purified from tetramer and dimer species using size-exclusion chromatography on a Superdex 200 Increase 10/300 column (Cytiva). Pure fractions, as determined by SDS-PAGE, were combined and stored at −80 °C. Histone tetramers (H3-H4)_2_ were prepared the same way as octamers by using only lyophilized H3 and H4.

### Reconstitution of Mononucleosomes

Mononucleosomes were formed as previously described (30, 31, 32) with some modifications. A plasmid containing 12 repeats of the Widom 601 DNA sequence (33) separated by 30 bp linkers was used to transform DH5α cells and the DNA was purified *via* phenol-chloroform extraction. DNA was digested into 177 bp repeats using ScaI (New England Biolabs) and purified. DNA pellets were dissolved in 10 mM Tris-HCl pH 8.0, 0.1 mM EDTA and stored at −20 °C. The DNA was mixed with histone octamers in a 1.2:1 ratio in 2 M TEK buffer (10 mM Tris-HCl pH 7.5, 2 M KCl, 0.1 mM EDTA, 1 mM DTT) and dialyzed into 10 mM Tris-HCl pH 7.5, 10 mM KCl, 1 mM DTT at 4 °C. Nucleosomes were analyzed on a 5% TBE gel gel to confirm DNA-octamer interaction forming nucleosomes (30).

### Experimental Design and Statistical Rationale

Having obtained all of the proteins and substrates necessary for the study, the experimental design involved three components. (1) At least two biological replicates of each *in vitro* ubiquitylation assay was performed and analyzed by SDS PAGE and mass spectrometry. ImageJ (https://imagej.net/nih-image/) was used to measure the remaining histones relative to the histone bands present at t = 0 (100%) included on the same gel. Results are reported with standard deviations of the average of at least two independent experiments. (2) Bands and/or high molecular weight species were excised and subjected to in-gel trypsin digestion followed by mass spectrometry. Two experiments were performed, and three time points were collected. Only those peptides identified in at least two independent experiments and at least two time points are reported. (3) Fluorescence anisotropy was used to determine the binding affinity of the histones for the ASB9-CRL5. In these experiments, the histones were fluorescently labeled at a specific cysteine and increasing concentrations of ASB9-CRL5 were then added to a constant concentration of the labeled histones. At least two experiments were performed and the average and standard deviations are reported.

### Nucleosome Ubiqiutylation

Reactions to assess histone ubiquitylation in mono-nucleosomes were performed for 1.5 h at 37 °C in a buffer containing 20 mM Tris-HCl pH 8.0, 200 mM NaCl, 5 mM MgCl_2_, 1 mM DTT, and 2 mM ATP with 0.2 μM UBE1, 0.5 μM UBE2D2, 50 μM Ub, 2 μM mono-nucleosomes, 0.5 μM NEDD8-ASB9-CRL5 or 0.5 μM ASB9-CRL5 and/or 0.5 μM ARIH2-UBE2L3. Quenched reactions were run on a 4 to 20% Mini-PROTEAN TGX Precast SDS-PAGE gel. Two biological replicates were analyzed. Reactions comparing ubiquitin activity of ASB9-ELOB/C-CUL5-RBX2 (ASB9-CRL5), CUL5-RBX2, CUL1-RBX1 were also done in a similar manner except incubation was only for 30 min with octamers and tetramers (n = 2 biological replicates).

### Assessing Histone Ubiquitylation Specificity

Reactions to assess ubiquitylation of free histones were carried out in a buffer containing 20 mM Tris-HCl pH 8.0, 200 mM NaCl, 5 mM MgCl_2_, 1 mM DTT, and 2 mM ATP with 0.2 μM UBE1, 0.5 μM UBE2D2, and/or ARIH2+UBE2L3, 50 μM Ub, and 0.5 μM NEDD8-ASB9-CRL5 or 0.5 μM ASB9-CRL5 with CUL5(K724R) at 37 °C. Reactions were initiated by addition of an E2-E3 master mix for concentrations of 0.5 μM UBE2D2 and 0.5 μM ASB9-CRL5. Reactions were quenched at various time points by adding 2X SDS-PAGE buffer and boiling at 90 °C for 10 min prior to separation on a 4 to 20% Mini-PROTEAN TGX SDS-PAGE gel. The degree of ubiquitylation was measured by quantifying band intensity of all unmodified histones at various time points compared to them at t = 0 using ImageJ (https://imagej.net/nih-image/). The mean and standard deviation were calculated from three biological replicates from different protein preparations.

Control reactions to assess the specificity of histone ubiquitylation were performed for 30 min at 37 °C in the same buffer with 0.2 μM UBE1, 0.5 μM UBE2D2, and 50 μM Ub as controls with either 4 μM H2A, 4 μM H2B, 4 μM H3, 4 μM H4, 4 μM H2A/H2B dimers, or 2 μM histone octamers. Reactions were initiated by adding 0.5 μM NEDD8-ASB9-CRL5. Two biological replicates were analyzed.

Reactions with the Asf1-H3-H4 complex were performed in 20 mM Tris-HCl pH 8.0, 300 mM NaCl, 5 mM MgCl_2_, 1 mM DTT, and 2 mM ATP.

### Binding Assays Using Fluorescence Anisotropy

Fluorescently-labeled histone monomers were prepared as follows. Single lyophilized histone monomers of H3 C110 or H4 (V22C) were resuspended at a concentration of ∼2 mg/ml in unfolding buffer (20 mM Tris-HCl pH 7.5, 7 M guanidine HCl, 25 mM NaCl, 0.5 mM TCEP). Oregon Green 488 Maleimide (OG488, Invitrogen O6034), was prepared as a 100 mM stock in dimethylformamide and added to the histone suspension to achieve 1 molar equivalent and allowed to incubate overnight at 4 °C. To remove remaining free OG488, 500 μl samples were injected onto a Superdex 200 Increase 10/300 column (GE Life Sciences) equilibrated in unfolding buffer at 4 °C. Fractions were analyzed by SDS-PAGE and fractions containing pure labeled histones were pooled dialyzed, lyophilized, and stored at −80 °C.

Labeled histones were dissolved in unfolding buffer at ∼1 mg/ml and dialyzed overnight into buffers of various salt concentrations (20 mM Tris-HCl pH 7.5, 10 mM NaCl or 150 mM NaCl, 5% glycerol, 1 mM DTT) just prior to fluorescence anisotropy experiments. Protein concentration and labeling efficiency was assessed by measuring the absorbance at 280 nm and 495 nm on a Nanodrop spectrophotometer.

Black 96-well plates (Greiner Bio 675,076) were first prepared by incubating 20 μl 5.5 mg/ml BSA in each well for 20 min at 25 °C. OG488-labeled histones (5 nM) were mixed with various concentrations of ASB9-CRL5 or NEDD8-ASB9-CRL5 complexes in triplicate in the coated wells and incubated at 25 °C for 1 to 2.5 h. Fluorescence anisotropy was measured at 25 °C on a SpectraMax iD5 Multi-Mode Microplate Reader (Molecular Devices) using an excitation wavelength of 485 nm, an emission wavelength of 535 nm, and an integration time of 100 ms. Anisotropy was calculated using r = [I_(V,V)_ − GI_(V,H)_]/[I_(V,V)_ − 2GI_(V,H)_], where r is anisotropy, I_(V,V)_ is the fluorescence intensity in the parallel direction, I_(V,H)_ is the fluorescence intensity in the perpendicular direction, and G, the grating factor used for calculating the anisotropy is 1.0, as previously determined for the instrument measurement pathway.

Fluorescence anisotropy data for the histones were fit to the following equation to determine the equilibrium binding affinity:

$$y=A\frac{\left({\left[Histone\right]}_{0}+x+K_{D}-\sqrt{{\left({\left[Histone\right]}_{0}+x+K_{D}\right)}^{2}-4{\left[Histone\right]}_{0}x}\right)}{2{\left[Histone\right]}_{0}}$$

where y is the change in fluorescence anisotropy, A is the maximum change in fluorescence anisotropy, x is the varying concentration of ASB9-CRL5 complex, [Histone]_0_ is the concentration of histone (5 nM), and K_D_ is the equilibrium binding affinity. A-values were determined for each individual set of triplicates due to slight variability in the anisotropy plateau values. The fluorescence anisotropy graphs show data from all experiments of a single condition fit to the above equation.

At least two biological replicates for each condition were conducted on separate days with different histone and ASB9-CRL5 complex preparations. For each assay, data were fit to the aforementioned equation to determine the K_D_. The reported K_D_ values are the mean and SEM of the K_D_ values determined for each of the two or three replicate experiments. Normalized data from all 3 sets of triplicates were plotted.

### Ubiquitylation Analysis by Mass Spectrometry

The aforementioned ubiquitylation reactions were carried out for 2, 5, and 10 min, quenched by addition of gel-loading buffer, and electrophoresed on a 13% Tris-glycine SDS-PAGE gel and stained with Coomassie Brilliant Blue G-250 (Thermo Fisher Scientific 20,279). Gel bands were excised and cut into small pieces and washed according to standard procedures (34). In-gel proteins were acetylated with 5 μl acetic anhydride and brought to pH ∼7 with ∼150 uL 1 M ammonium bicarbonate followed by 1 h incubation at 37 °C. The cysteines were reduced and alkylated with 1.36 mg/ml chloroacetamide in 100 mM ammonium bicarbonate, 8 mM TCEP, washed and immediately digested with trypsin for 30 min at 4 °C, then 37 °C overnight. The supernatant containing tryptic peptides was collected (34). Samples were dried in a speed-vac and stored at −20 °C until analysis.

Trypsin-digested peptides were analyzed by ultra high pressure liquid chromatography coupled with tandem mass spectrometry (LC-MS/MS) using nano-spray ionization on an Orbitrap fusion Lumos hybrid mass spectrometer (Thermo Fisher Scientific) interfaced with nano-scale reversed-phase (Thermo Dionex UltiMate 3000 RSLC nano System) using a 25 cm, 75-micron ID glass capillary packed with 1.7-μm C18 (130) BEHTM beads (Waters corporation). Peptides were eluted from the C18 column into the mass spectrometer using a linear gradient (5–80%) of Acetonitrile (ACN) at a flow rate of 375 nl/min for 1.5 h. The buffers used to create the ACN gradient were: Buffer A (98% H_2_O, 2% ACN, 0.1% formic acid) and Buffer B (100% ACN, 0.1% formic acid). Data dependent acquisition mode was used for data collection and the mass spectrometer parameters were as follows; the MS1 survey scan using the orbitrap detector (mass range (m/z): 400–1500 using quadrupole isolation, 60,000 resolution setting, spray voltage of 2200 V, ion transfer tube temperature of 275 °C, AGC target of 400,000, and maximum injection time of 50 ms). Data dependent scans (top speed for most intense ions, with charge state set to only include +2- +5 ions, and 5 s exclusion time, while selecting ions with minimal intensities of 50,000 in which the collision event was carried out in the high energy collision cell (HCD Collision Energy of 30%), and the fragment masses were analyzed in the ion trap mass analyzer (with ion trap scan rate of turbo, first mass m/z was 100, AGC Target 5000 and maximum injection time of 35 ms). Protein identification was carried out using Peaks Studio 11.5 build 20,231,206 (Bioinformatics solutions Inc.) searched against a custom database consisting of EcoliK12_DH10B_NCBI_100808.fasta with the sequences of human CUL5, EloB, EloC, ASB9, Ub, and *xenopus laevis* histones H2A, H2B, H3 and H4 added. The number of entries searched was 4149. The Search parameters were: Precursor Mass Error Tolerance: 10.00 ppm, Fragment Mass Error Tolerance: 0.02 Da, Max Missed Cleavage: 2, Max Variable post-translational modification per Peptide: 2. Carbamidomethylation (+57.02) was the only fixed modification and variable modifications included: acetic anhydride (K) (+42.01), ubiquitin (K) (+114.04), oxidation (M) (+15.99), and deamidation (N, Q) (+0.98). Peptide −10logP >15, Protein -logP >15, Proteins unique peptides >1, De novo score (%) >50%. Although the data for the mono- and di-ubiquitylated samples are included, our analysis focused on the high molecular weight “smear” containing the polyubiquitylated species. The statistics of the filtered results were different for each sample. Ubiquitylation of lysines was monitored over 2, 5, and 10 min ubiquitin reactions. For the high molecular weight samples 9 (2 min); FDR 0.6%, FDR protein group 6.9%. For sample 10 (5 min); FDR 0.6% and FDR protein group 8.9% and for sample 11 (10 min); FDR 0.5% and FDR protein group 14.3%.Ubiquitin modifications were observed through the trypsin product “GG” left on ubiquitylated lysines, with mass of 114.04 Da. A cutoff score of (−10logP) > 14 (*p*-value <0.040) was used to validate post-translational modifications. Only those modifications that were observed in at least two of the three timepoints are reported.

## Results

### Histones in Nucleosomal Core Particles are not the Preferred Substrate for ASB9-CRL5

**Fig. 1:**
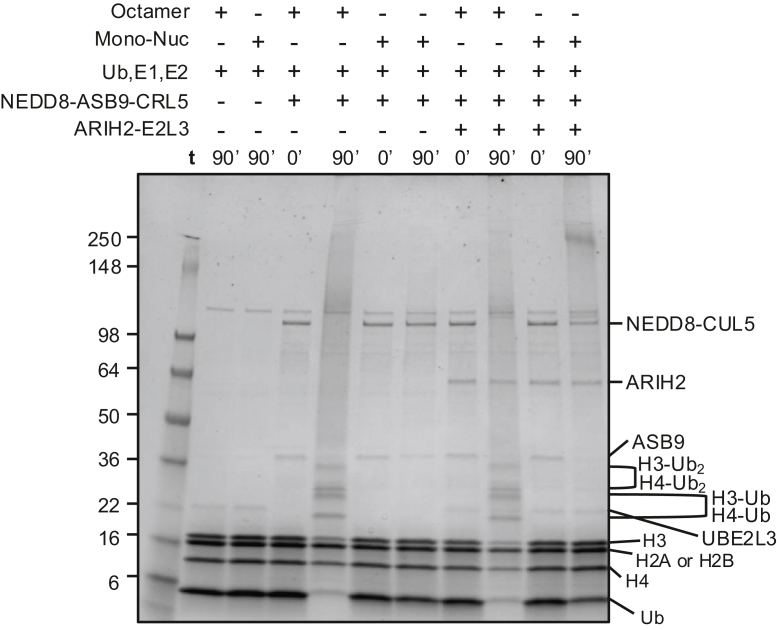
**ASB9-CRL does not ubiquitylate histones in mononucleosomes.** NEDD8-ASB9-CRL5 ubiquitylation assays using nucleosomes with or without an additional E3 ligase, ARIH2 and its obligate E2, UBE2L3, required for ubiquitylation of another ASB9 substrate, CKB. Representative image of n = 2 independent experiments. ASB9, ankyrin and SOCS box protein 9; CKB, creatine kinase; NEDD8, neural precursor cell expressed developmentally down-regulated.

Because all core histones were highly enriched in an ASB9 pull-down (8), initial ubiquitylation assays were performed with mononucleosomes as substrates. Mononucleosomes were assembled using previously established methods using a 177 bp DNA sequence comprised of a high-affinity 147 bp Widom sequence with 30 bp linkers mixed with histone octamers. Nucleosome formation was confirmed by native gel (Supplemental Fig. S1). To assay ubiquitylation of mononucleosomes, they were incubated with NEDD8-ASB9-CRL5 at 37 °C, but no ubiquitylation was observed even after 1.5 h (Fig. 1). Addition of ARIH2 and its obligate E2, UBE2L3, to reactions still did not elicit any histone ubiquitylation.

### Histones H3 and H4 are Poly-ubiquitylated by the ASB9 Cullin5 E3 Ligase

**Fig. 2:**
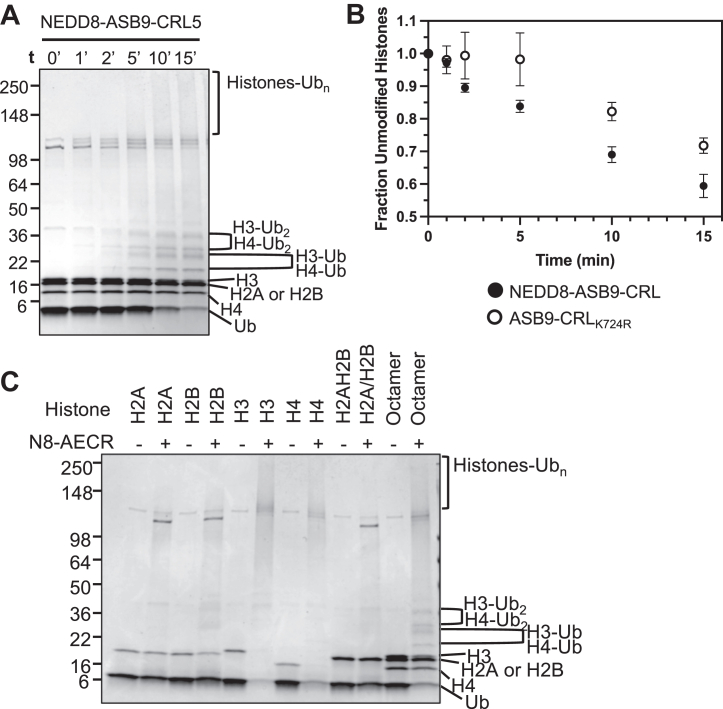
***In vitro* ubiquitylation assays of histone octamers shows specificity towards Histones H3 and H4.***A*, time course of histone octamer ubiquitylation by NEDD8-ASB9-CRL5. Covalently ubiquitin-modified histones appeared as higher weight mono-ubiquitylated bands, di-ubiquitylated bands, and poly-ubiquitylated high-molecular weight smears. *B*, plot of decrease in histone octamers when reacted with either NEDD8-ASB9-CRL (corresponding to the gel shown in *panel A*) and an unneddylated ASB9-CRL5 using a K724R CUL5 mutant. ImageJ was used to quantify band intensity of all remaining unmodified histones from biological triplicates using separate protein preparations. The fraction of unmodified histones approached 0.5 because the gel band of histones H2A/H2B, which ran together, did not appear to decrease over time. *C*, 30 min ubiquitylation reactions of individual histones, H2A/H2B dimer, and histone octamers. Ubiquitylation of octamer samples showed a strong preference towards H3 and H4. Reactions with H2A and H2B, as monomers or dimers, showed minimal ubiquitylation. Note that the H3 and H4 bands disappear by 30 min whereas the H2A and H2B bands are still present. In addition, the Ub_n_ polyubiquitin smears are much darker whenever H3 and H4 are present. Images are representative of at least n = 3 biological replicates. H3, histone 3; H4, histone 4; H2A, histone 2A; H2B, histone 2B; NEDD8, neural precursor cell expressed developmentally down-regulated.

We next attempted to ubiquitylate extranucleosomal histones. Reactions were performed with histone octamers against ASB9-CRL5 with and without a NEDD8 post-translational modification (neddylation) of Cullin5 which has been shown to enhance Cullin-mediated substrate ubiquitylation (35, 36, 37). Assays with NEDD8-ASB9-CRL5 showed mono- and di-ubiquitylation of histones H3 and H4 by 2 min as identified by mass spectrometry (Fig. 2*A*). Polyubiquitylation of histones H3 and H4 was also very rapid (Fig. 2*A* and Supplemental Tables S1–S3). To analyze the effect of neddylation on ASB9-CRL5 ubiquitylation, a Cullin5 K724R mutant that cannot be neddylated was also used (Fig. 2*B*). Neddylation was not required for histone ubiquitylation, but the neddylated ASB9-CRL5 ubiquitylated histones more efficiently (Fig. 2*B*).

Previous studies showed dissociation of histone octamers into H2A/H2B dimers and H3 and H4 monomers (38), which would be expected in our experimental conditions, so it is possible that the ASB9-CRL5 only ubiquitylates monomeric histones. To test this hypothesis, ubiquitylation assays with NEDD8-ASB9-CRL5 were performed on individual histone monomers, H2A/H2B dimers, and histone octamers. Again, the poly-ubiquitylated species were produced from histones H3 and H4 (Fig. 2*C*). The bands corresponding to monomeric H3 and H4 completely disappeared, whereas monomeric histones H2A and/or H2B remained mostly unmodified. This is consistent with the mass spectrometry results which showed no observable ubiquitylation of H2B and less than 10% of the peptides in any sample corresponding to ubiquitylated peptides from H2A. These results indicated that ASB9-CRL5 histone ubiquitylation strongly prefers histones H3 and H4 as substrates. Finally, when the histone chaperone, Asf1 was in complex with histones H3 and H4, no histone ubiquitylation was observed (Supplementaly Fig. S2). This result further substantiated that free, extranucleosomal H3 and H4 were the preferred substrates of ASB9-CRL5. H3 and H4 ubiquitylation in the absence of ASB9 or Elongins B/C was similar to ubiquitylation by CUL1-RBX1, and lower than observed for the ASB9-CRL5 (Supplemental Fig. S3, *A* and *B*).

### Identification of Poly-ubiquitylated Histone Species

**Fig. 3:**
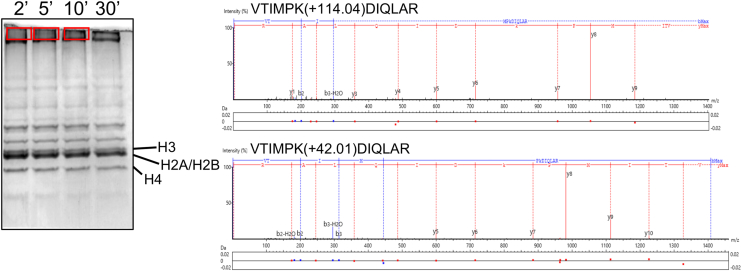
**Mass spectrometry identifies specific ubiquitylated lysines.***In vitro* ubiquitylation PTM analysis workflow. The same 2, 5, and 10 min ubiquitylation reactions in Fig. 2 of histone octamers with NEDD8-ASB9-CUL5-ELOB/C-RBX2 were run on a 13% SDS-PAGE gel for 45 min and stained with *Coomassie Blue*. Individual bands of polyubiquitylated species from the gel (marked by boxes) were excised, acetylated, alkylated, and trypsin digested before analysis using LC-MS/MS. Two example MS2 spectra of ubiquitylated and acetylated (unmodified) H3 peptide 117 to 128 are shown, illustrating identification of modification. Here, the m/z differences between the y6 and y7 ions reveal m/z changes relative to unmodified peptides used to identify post-translational modification. NEDD8, neural precursor cell expressed developmentally down-regulated; PTM, post-translational modification; RBX, RING-Box protein.

MS/MS analysis was performed on bands isolated from the gel shown in Figure 2*A* to identify the ubiquitylated histone species based on the presence of the ‘GG’ (114.04 Da) modification on lysine. The mono- and di-ubiquitylated species labeled in Figure 2*A* were confirmed, and histone species in the high molecular weight smear were identified (Fig. 3). Unmodified lysines were identified by acetylation as indicated by a modification of 42.01 Da (Fig. 3).

The high molecular weight bands were mostly composed of Ub as indicated by the large number of Ub peptides (57, 71, 79 peptides were observed in the 2, 5, and 10 min samples) and the large number of peptides with di-Gly modifications on K48 (10 ± 1 peptide across time points) and/or K63 (7, 11, 16 peptides were observed in the 2, 5, and 10 min samples) (Table S1-S3). All of the lysines in Ub were covered in the mass spectrometry data. However, lysines 6, 11, 27, 29, and 33 were never observed to be ubiquitylated. These results strongly indicate that the ASB9-CRL5 with UBE2D2 only installed K48 and K63 linkages.

Histones H3 and H4 were rapidly polyubiquitylated. By 2 min, we observed 16 unique ubiquitylated peptides for H3 and 18 for H4 (Supplemental Table S1), whereas only one unique ubiquitin-modified peptide was observed from histone H2A in the 5 and 10 min sample. This peptide, with a ‘GG’ on K95 was mainly observed with an acetylation at the same lysine. Although such analyses are not strictly quantitative, the H2A ‘GG’ peptide represented less than 10% of the total ion counts. These low amounts of ubiquitylation contrast with the results for histones H3 and H4 in which several lysines are over 50% ubiquitylated by similar calculations (7, 39).

**Fig. 4:**
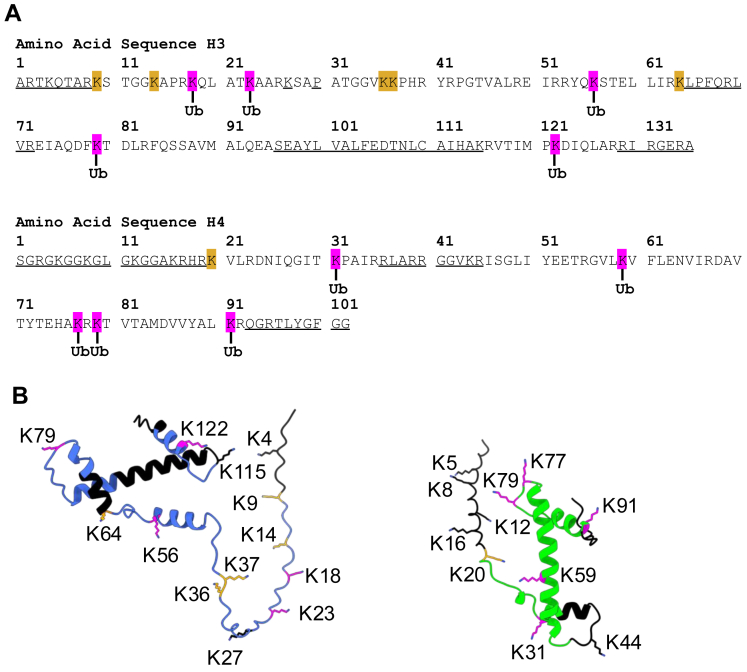
**Histones H3 and H4 ubiquitylation pattern.***A*, primary amino acid sequence of histones *Xenopus laevis* H3 and H4. Identified lysines modified with ubiquitin are shown in *magenta*. Unmodified lysines that were covered by MS data were shown in *orange*, while sequences that were not covered by mass spectrometry were underlined. *B*, structures of histones H3 (*blue*) and H4 (*green*) were predicted by Alphafold and the identified lysines were mapped onto the structure (modified with ubiquitin in *magenta*, unmodified lysines in *orange*, and regions unidentified by MS in *black*). H3, histone 3; H4, histone 4.

Mapping of the ubiquitylated lysines onto the primary sequence and structures of H3 and H4 predicted by Alphafold (Fig. 4, *A* and *B*) showed that ubiquitylated lysines were present in both the disordered histone tails (H3K18, H3K23), and the core histone fold (H3K56, H3K79, H3K122, H4K31, H4K77, H4K79 and H4K91) (40). Importantly, ubiquitylation was not observed in some lysines that were covered in the mass spectrometry data (H3K9, H3K14, H3K36, H3K37, H3K64, H4K20), indicating that the ASB9-CRL5 has some apparent substrate specificity for which lysines it modifies. Of the ubiquitylated lysines we identified, all have been reported in high throughput proteomics experiments (Phosphosite.org) and one, H3K23 has also been identified in a low throughput experiment (41). Recently, Skrajna *et al*. observed the anaphase promoting complex/cyclosome (APC/C) -mediated ubiquitylation of extranucleosomal histone complexes (42). These authors showed that APC/C binds the acidic patch on the nucleosome, but it does not ubiquitylate nucleosomal histones. Instead, it ubiquitylates all four (H3/H4 and H2A/H2B) histones at multiple lysines only when they are extranucleosomal. In contrast, the ASB9-CRL5, which also ubiquitylates extranucleosomal histones, appears to be strongly biased towards ubiquitylation of only histones H3 and H4.

### Quantitative Determination of H3 and H4 Binding Affinity to ASB9-CRL5 Ligases

**Fig. 5:**
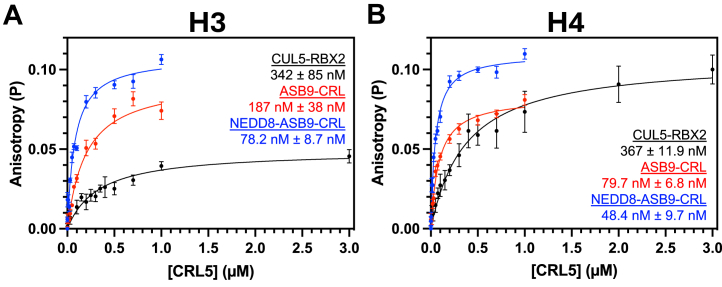
**Fluorescence Anisotropy of histones H3 and H4 with CRL5.** Fluorescence anisotropy with *Oregon Green* 488 labeled histones (*A*) H3 and (*B*) H4V22C showed nM affinity against CUL5-RBX2, ASB9-CRL5, and NEDD8-ASB9-CRL5 at 10 mM NaCl. Slight binding enhancements for both histones were observed upon neddylation of CUL5. Data represent the mean and SEM of at least two biological replicates and the reported K_D_ values are the mean and SEM of K_D_ calculated from each individual experiment. NEDD8, neural precursor cell expressed developmentally down-regulated; H3, histone 3.

To determine whether the extent of ubiquitylation was related to binding affinity, we measured equilibrium binding affinities of NEDD8-ASB9-CRL5 and ASB9-CRL5 to monomeric histones H2A, H2B, H3, and H4 by fluorescence anisotropy. The single cysteine in histone H3, C110, and installed cysteines in histone H2A K119C, H2B T115C, H4 V22C were fluorescently labeled with the cysteine-reactive Oregon Green 488 Maleimide, purified, and a constant amount of each labeled histone was mixed with increasing amounts of NEDD8-ASB9-CRL5, ASB9-CRL5, or CUL5-RBX2. Preliminary experiments revealed a strong electrostatic dependence on the binding affinities, as experiments performed at 150 mM NaCl did not produce quantifiable binding affinities whereas decreasing the salt concentration to 10 mM NaCl significantly enhanced ASB9-CRL5 binding of H3 and H4 (43). ASB9-CRL5 bound to H3 and H4 with K_D_ of 187 ± 38 nM and 79.7 ± 6.8 nM respectively and neddylated ASB9-CRL5 showed an approximate 2-fold enhancement of binding affinity with K_D_ of 78.2 ± 8.7 nM and 48.4 ± 9.7 nM respectively (Fig. 5, *A* and *B*). Binding to ASB9-ELOB/C was too weak and could not be accurately measured. This result was surprising because previous studies showed ASB9-CRL5 bound to another substrate, CKB, with sub-nanomolar affinity (44). Intriguingly, CUL5-RBX2 alone bound to histones H3 and H4 but with lower affinity than the full ASB9-CRL5 ligases (Fig. 5, *A*and *B*). Binding experiments using histone H2A, which was not a preferred substrate, had a binding affinity of 341 ± 42 nM to ASB9-CRL5 (Fig. S4). Binding could not be measured for ASB9-CRL5 to histone H2B, which was never observed to be ubiquitylated.

### ARIH2 is Not Required for Histone Ubiquitylation

**Fig. 6:**
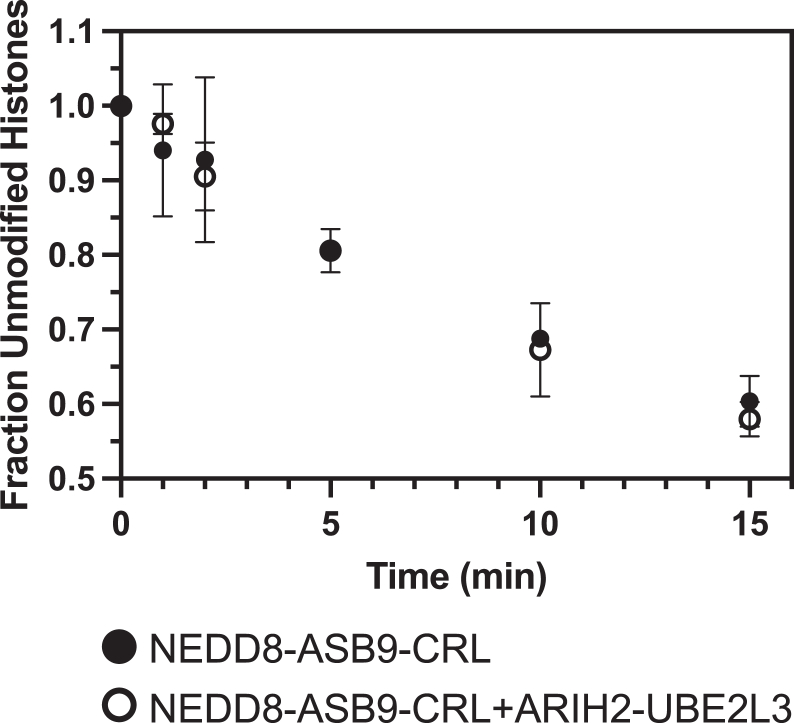
**ARIH2 is not required for ubiquitylation of histones.** The RING-between-RING ligase ARIH2 along with E2 UBEL23 have been shown to be required for ubiquitylation of certain CRL5 substrates. Ubiquitylation reactions of octamers with NEDD8-ASB9-CRL5 ± ARIH2-UBE2L3 did not affect ubiquitylation. ImageJ was used to quantify band intensity of all remaining unmodified histones from two biological replicates using separate protein preparations. NEDD8, neural precursor cell expressed developmentally down-regulated.

Previous studies showed that the RING-between-RING E3 ligase, ARIH2 was essential for ASB9-CRL5 ubiquitylation of CKB (16, 17). To test if ARIH2 participated in NEDD8-ASB9-CRL5 ubiquitylation of histones, ubiquitylation assays were performed with ARIH2-UBE2L3 added to the NEDD8-ASB9-CRL5. No differences were observed in the amount of ubiquitylation as compared to reactions with only NEDD8-ASB9-CRL5 (Fig. 6), showing that histone ubiquitylation is not ARIH2 dependent.

## Discussion

Our results show that ASB9-CRL5 polyubiquitylates primarily histones H3 and H4. Given that the other *bona fide* substrate of the ASB9-CRL5 is the metabolic enzyme, CKB, this finding reveals that the ASB9-CRL5 can ubiquitylate structurally diverse targets (45).In contrast to the requirement of ARIH2 for ubiquitylation of CKB, ASB9-CRL5-mediated polyubiquitylation of histones does not require ARIH2. This is the first demonstration of rapid polyubiquitylation by a CRL5 ligase that does not require ARIH2. The Schulman group has proposed that ARIH proteins are required for ubiquitylation of well-folded substrates because when bound to the substrate receptor, they are too far from the Ub ∼ E2s (13, 15). Our observation that histone ubiquitylation does not require ARIH2 is consistent with the idea that histone monomers, which are disordered (46, 47, 48) might be able to directly interact with the Ub ∼ E2.

Another contrast between ubiquitylation of CKB vs. histones by ASB9-CRL5 was that CKB bound with extremely high affinity to the substrate receptor, ASB9 (44). Histones appeared to bind CUL5-RBX2, even without a substrate receptor, although with two-fold lower affinity to ASB9-CRL5 and four-fold lower affinity to NEDD8-ASB9-CRL5. These results suggest that the disordered histones may be interacting with several components of the ASB9-CRL5 including the CUL5 (44). In our previous work on CKB ubiquitylation by the ASB9-CRL5, we showed that neddylation was primarily required for opening the binding site for ARIH2. Our results showed a conformational equilibrium between the “open state” for ARIH2 binding and the closed state that was shifted upon neddylation (17). Although it was previously thought that NEDD8 functions as an on-off switch, in the case of CUL5, its effects may be more subtle. In fact, the Schulman group has recently observed both of these states in cryoEM structures of RBX1 in a neddylated Cullin-1 ligase (35). Here we see that for histones, neddylation improves binding and improves ubiquitylation while the unneddylated ASB9-CRL5 is still capable of ubiquitylation.

H3 and H4 were rapidly polyubiquitylated (within 2 min) by the ASB9-CRL5 and mass spectrometry analysis of the high molecular weight H3 and H4 species contained only K48 and K63 ubiquitin linkages. Since the functional consequence of K48 and K63 linked polyubiquitylation is proteasomal degradation (49, 50), it is likely that ASB9-CRL5 polyubiquitylation of histones H3 and H4 targets them for proteasomal degradation (49, 50). The ASB9-CRL5 is now the second example of extranucleosomal ubiquitylation of histones targeting them for degradation, the first being the APC/C complex reported earlier this year (42).

Ordinarily, histones are found stabilized in nucleosomes. All eukaryotes have the potential to generate excess histones (51), resulting in extranucleosomal histones that lead to deleterious effects including cell death (20). UV-mediated DNA damage causes nucleosomes to break down and histones to be released. In this situation also, histones H3 and H4 have been reported to be ubiquitylated (52). CUL5 has been shown to translocate into the nucleus to sites of DNA damage (27) and pol II stalling (26). Our work presents the first functional link between this nuclear translocation of CUL5 and one of its functions – ubiquitylation of histones. It was previously shown that UV-irradiation results in mono-ubiquitylation of histones H3 and H4 by CUL4, which causes them to be ejected from nucleosomes (22). It is possible that mono-ubiquitylation destabilizes the nucleosomes releasing the histones for ASB9-CRL5 polyubiquitylation.

DNA damage can also cause an accumulation of excess cytoplasmic histones (24, 53, 54). Previously, yeast have been shown to combat cytoplasmic histones through proteasomal degradation mediated by RING ligases, HEL1 and HEL2, and E2 Ubc4 (homologous to human UBE2D2) (24) and Ubc5 (homologous to UBE2D1). Thus, ASB9-CRL5 may also ubiquitylate free histones in the cytoplasm (49, 50, 55). Since the ASB9-CRL5 is found in both the nucleus and the cytoplasm, it could play a role in removing free histones in both locations during DNA damage.

The APC/C complex was recently reported to ubiquitylate histones (56). Although this report found ubiquitylation of both nucleosomal and extranucleosomal histones *in vitro*, more recent work has shown that only extranucleosomal histones are degraded by the APC/C complex (42). The APC/C complex is recruited to specific transcription start sites during mitosis and is important for chromatin remodeling during differentiation of pluripotent stem cells. Skrajna *et al.* reported that while histone stabilization in nucleosomes prevented ubiquitylation, stabilization by the chaperone Nap1 still allowed ubiquitylation. In our experiments, the ASB9-CRL5 was unable to ubiquitylate chaperone (Asf1)-bound histones H3 and H4, perhaps because the histones become sequestered in the Asf1 complex.

The first report of H3 ubiquitylation showed polyubiquitylation of H3 in elongating spermatids of rat testes (23). Polyubiquitylation of H3 is likely linked to degradation of histones to facilitate protamine-mediated chromatin compaction in the sperm head (57). In fact, testis is the organ showing the highest level of ASB9 expression (12) and expression of ASB9 increases over the maturation of spermatogonial stem cells into spermatids (58, 59). Low expression of ASB9 was observed in patients with abnormal spermatogenesis (58). It is likely that ASB9-CRL5 mediated histone H3 and H4 degradation is at least one of the functions of ASB9 that is required for normal spermatogenesis and chromatin compaction.

In conclusion, our results show, for the first time, that ASB9-CRL5 primarily polyubiquitylates histones H3 and H4 revealing a new function for the ASB9-CRL5. Ubiquitylation of free histones has been shown to be important for degradation of histones in normal spermatogenesis, the tissue where ASB9 expression is highest. Although all other CUL5 substrates known so far require the additional ring-between-ring ligase, ARIH2, our results show that the ubiquitylation of histones H3 and H4 by ASB9-CRL5 does not require ARIH2.

## Data Availability

All mass spectrometry data is available at MassIVE (massive.ucsd.edu) with the data set MSV000099427 and password a.

## Supplemental data

This article contains supplemental data (30).

## Conflict of interest

The authors declare no competing interests.

## Supplementary Data

TableS1

TableS2

TableS3

Supplementary Figures

SupplementaryMSspectra
