# Supplementary material for: The Mechanism of Histone Ubiquitylation by the ASB9-CUL5 Ubiquitin Ligase
Source: Mol Cell Proteomics. 2025 Nov 17;25(2):101471. doi: 10.1016/j.mcpro.2025.101471 (PMC12914668; doi:10.1016/j.mcpro.2025.101471)
Supplement: Supplementary Figures [file mmc4.pdf]

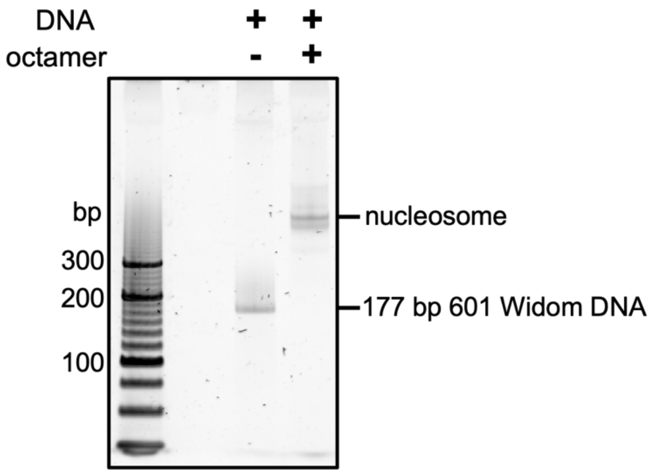

Supplementary Figure S1 –Nucleosomes were reconstituted by mixing a 177 bp dsDNA containing the 145 bp Widom sequence and 11 bp linkers along with histone octamers which were dialyzed overnight following previously established protocols (30).

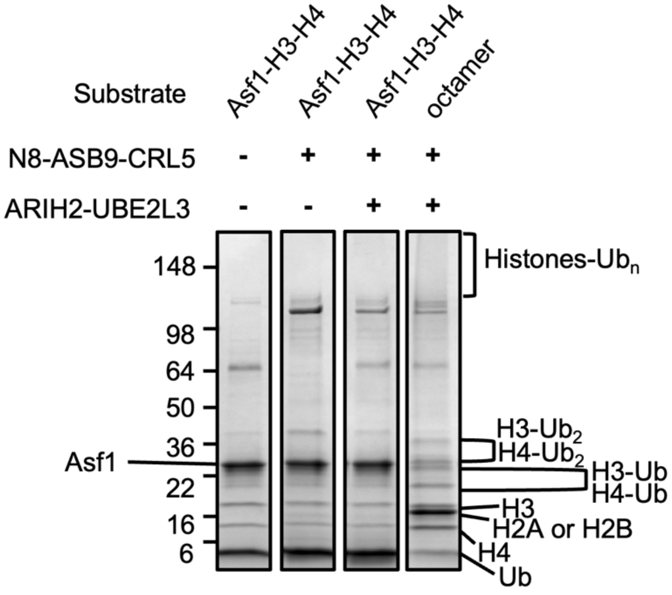

Supplementary Figure S2 – Ubiquitylation reactions with H3-H4-Asf1. Extranucleosomal histones H3 and H4 in complex with chaperone Asf1 were not ubiquitylated by N8-ASB9-CRL5, while inclusion of ARIH2-UBE2L3 similarly had no effect in contrast with octamer ubiquitylation. Gel Image is representative of n=2 biological replicates.

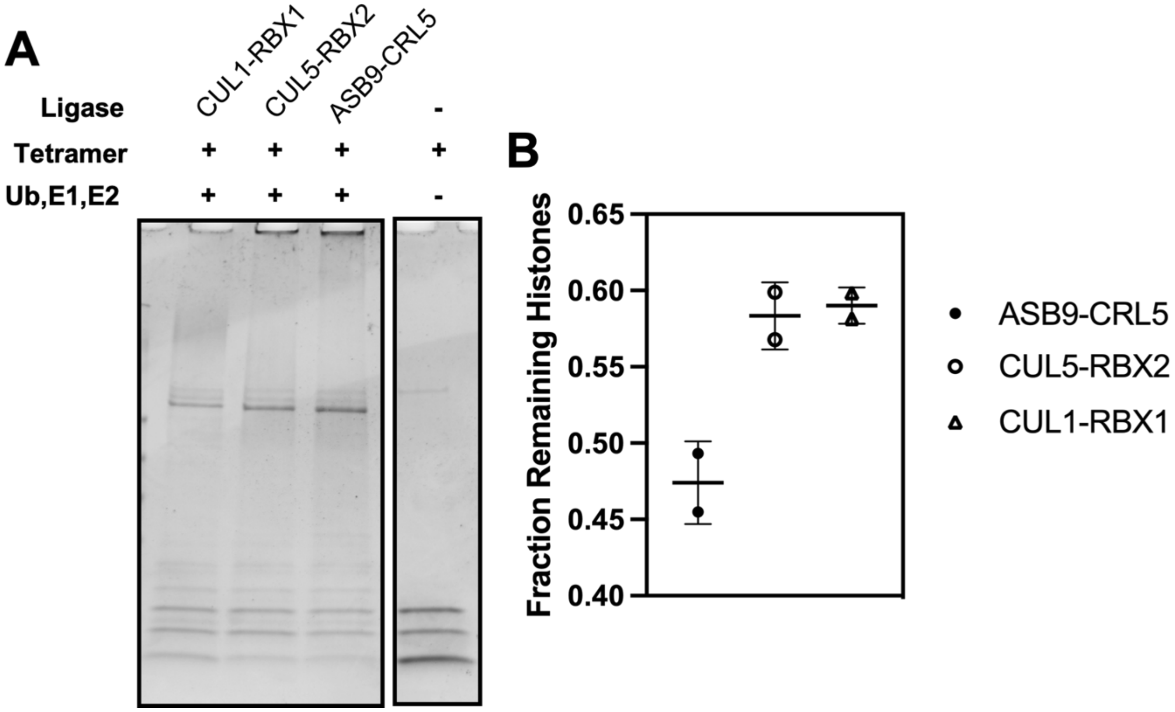

Supplementary Figure S3 – Ubiquitylation of H3 and H4 with various Cullin ligases. (**A**) Cul5-Rbx2 and Cul1-Rbx1 yields minimal ubiquitylation of H3 and H4, while inclusion of ASB9-EloB/C enhances ubiquitylation. (**B**) Histone ubiquitylation quantified against a control reaction (rightmost lane in **Figure S2A**) containing no ubiquitin machinery. Data is representative of at least two independent, biological replicates.

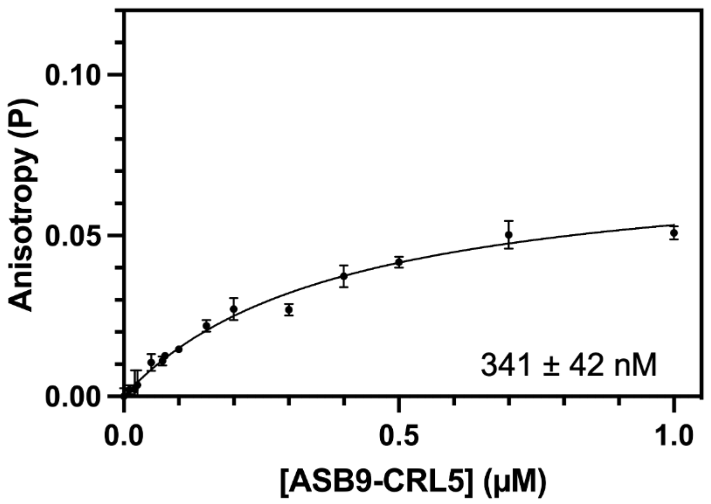

Supplementary Figure S4 – Fluorescence anisotropy with Oregon Green 488 labeled histone H2A-K119C binding to ASB9-CRL5 at 10 mM NaCl. The binding affinity was K_D = 341 ± 42 nM. Data represent the mean and SEM of n=2 biological replicates and the reported K_D values are the mean and SEM of K_D calculated from each individual experiment.
