# Supplementary material for: The Mechanism of Histone Ubiquitylation by the ASB9-CUL5 Ubiquitin Ligase
Source: Mol Cell Proteomics. 2025 Nov 17;25(2):101471. doi: 10.1016/j.mcpro.2025.101471 (PMC12914668; doi:10.1016/j.mcpro.2025.101471)
Supplement: SupplementaryMSspectra [file mmc5.pdf]

READ ME FILE FOR SUPPLEMENTARY MS SPECTRA

In this pdf file we include images of MS/MS spectra for histone H3, histone H4, and ubiquitin (showing the K48 and K63 linkages). The files are labeled with histone, which sample the data came from (sample 9=HMW 2 min, sample 10=HMW 5 min, sample 11=HMW 10 min). We did not repeat peptide spectra so if we already showed a spectrum in from sample 9, the 2 min data, we did not show it again from the 5 or 10 min data. The file names also are labeled either _s for spectrum or _d for the table of b and y ions that correspond to the spectrum.

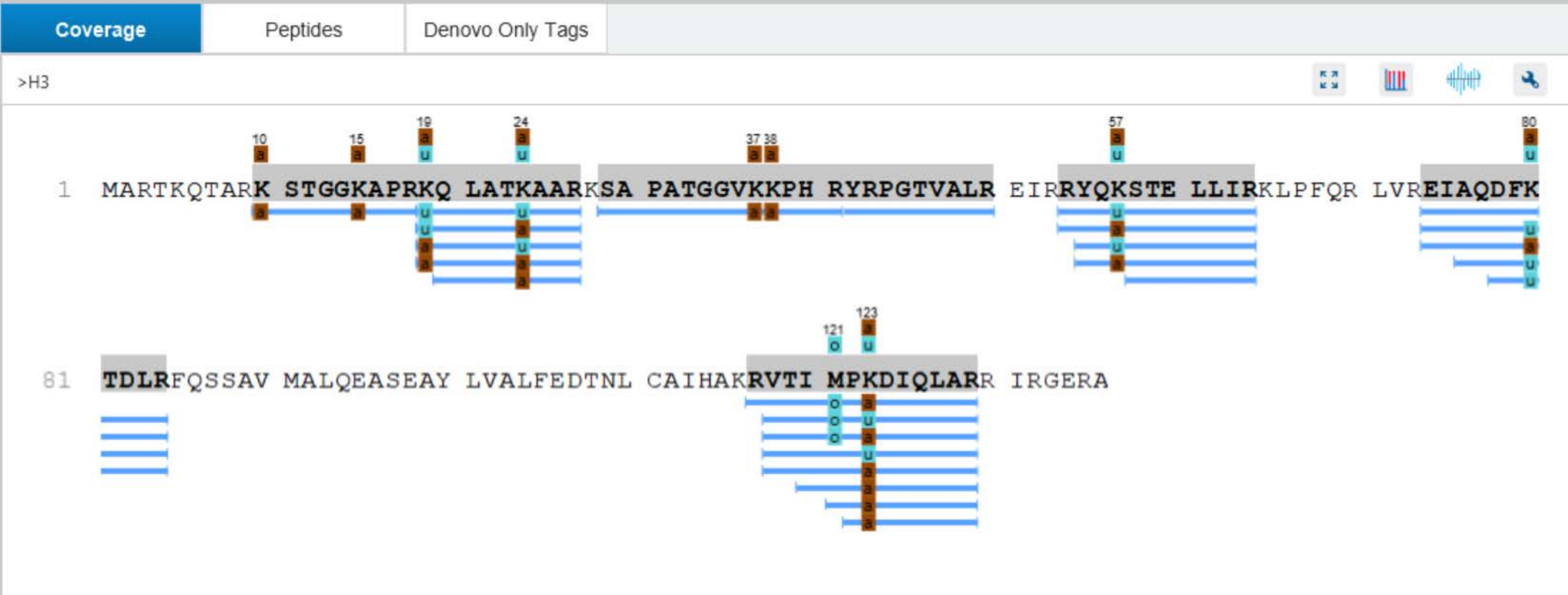
Coverage
Peptides
Denovo Only Tags
>H3
1 MARTKQTARK STGGKAPRKQ LATKAARKSA PATGGVKKPH RYRPGTVALR EIRRYQKSTE LLIRKLPFQR LVREIAQDFK
81 TDLRFQSSAV MALQEASEAY LVALFEDTNL CAIHAKRVTI MPKDIQLARR IRGERA

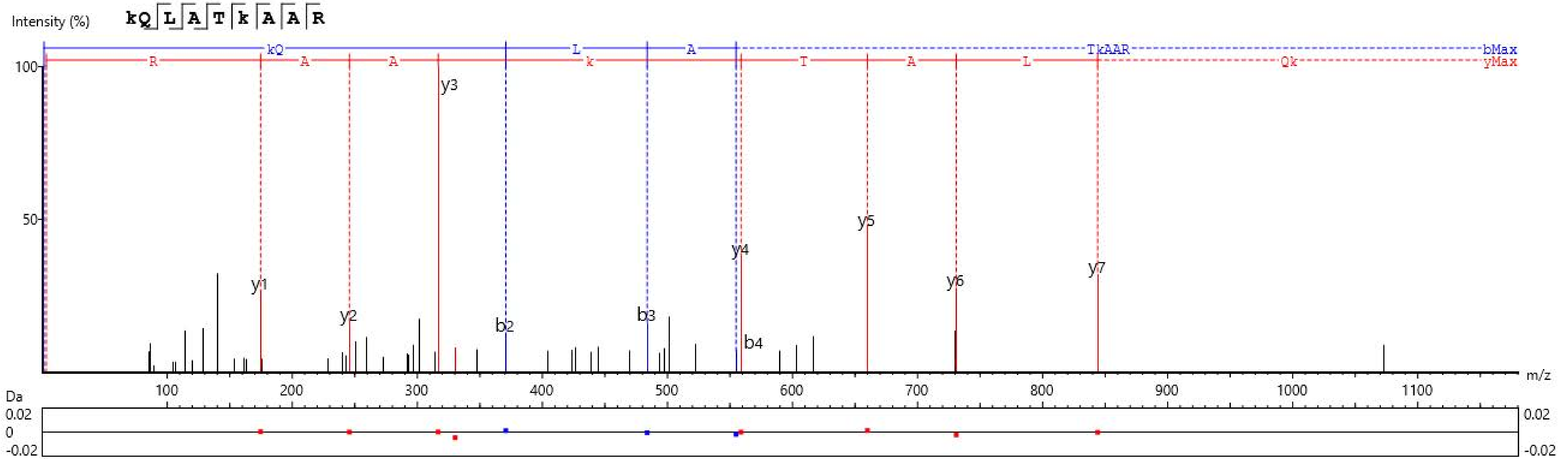

Intensity (%)
kQLATkAAR
kQ
L
A
TkAAR
bMax
R
A
A
k
T
A
L
Qk
yMax
100
50
y1
y2
y3
b2
b3
y4
b4
y5
y6
y7
m/z
100
200
300
400
500
600
700
800
900
1000
1100
Da
0.02
0
-0.02
0.02
-0.02

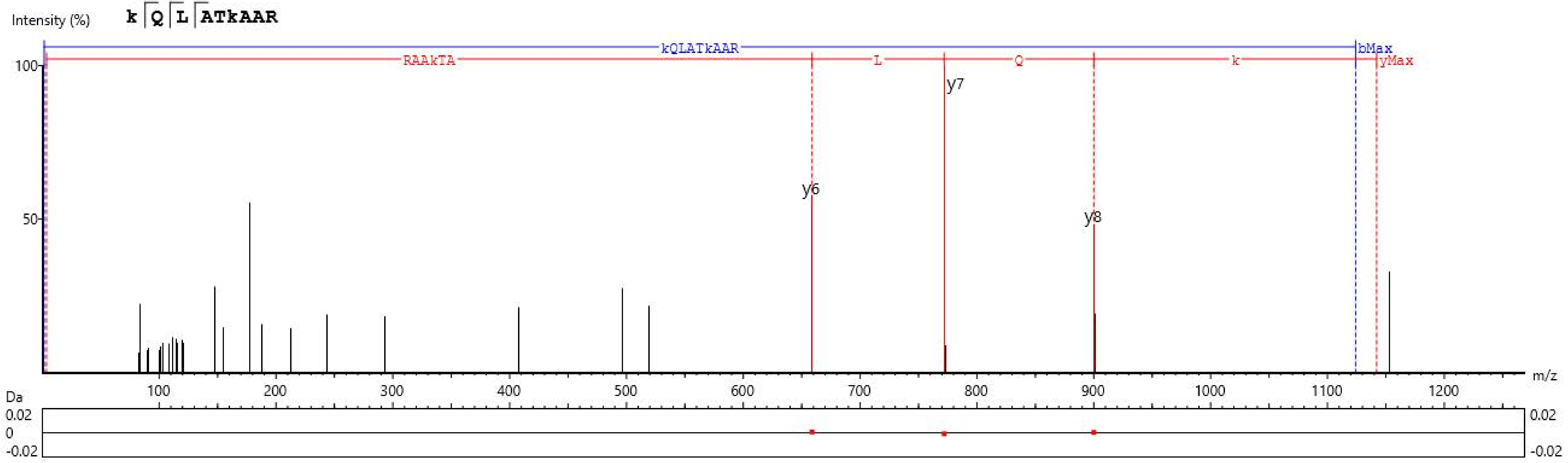

Intensity (%)
k Q L ATkAAR
kQLATkAAR
bMax
RAAkTA
L
Q
k
yMax
y7
y6
y8
m/z
Da

Sample 11 | CL_09.raw | Feature Id: 2804, m/z: 405.5703, RT: 10.84, CV:-60.0, z: 3, -10lgP: 35.38

Scan 1353, m/z=405.5703, z=3, RT=10.89, CV=-60.0, Length=9, -10lgP=35.38, ppm=0.1   1/1

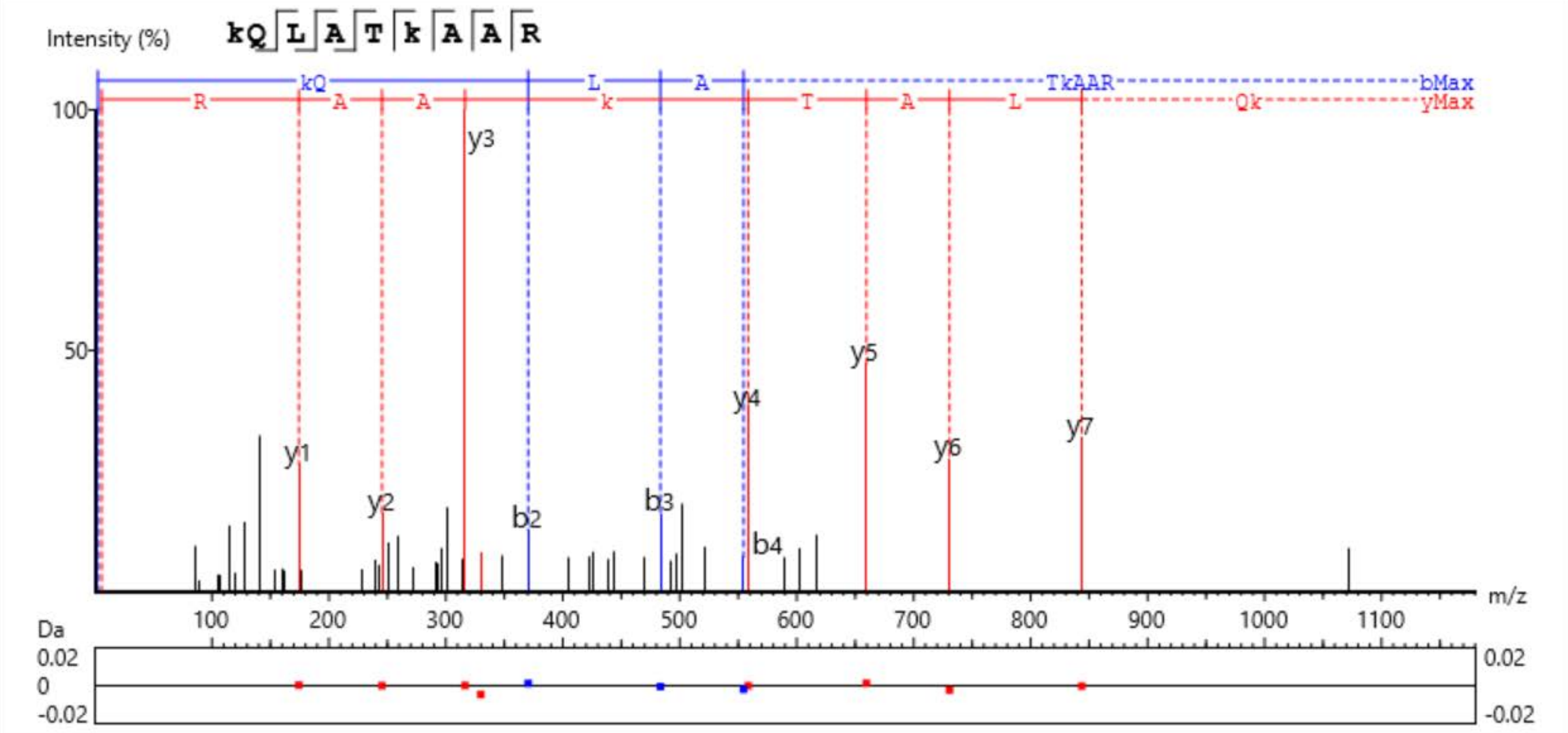

alignment   error map

Ion Match | Survey

#	b	b-H2O	b-NH3	b(2+)	Seq	y	y-H2O	y-NH3	y(2+)	#
1	243.15	225.14	226.12	122.07	K(+114.04)					9
2	371.20	353.19	354.18	186.10	Q	972.56	954.55	955.53	486.78	8
3	484.29	466.28	467.26	242.64	L	844.50	826.49	827.47	422.75	7
4	555.33	537.31	538.30	278.16	A	731.42	713.41	714.39	366.21	6
5	656.37	638.36	639.35	328.69	T	660.38	642.37	643.35	330.69	5
6	898.51	880.50	881.48	449.76	K(+114.04)	559.33	541.32	542.30	280.17	4
7	969.55	951.54	952.52	485.27	A	317.19	299.18	300.17	159.10	3
8	1040.59	1022.57	1023.56	520.79	A	246.16	228.15	229.13	123.58	2
9					R	175.12	157.11	158.09	88.06	1

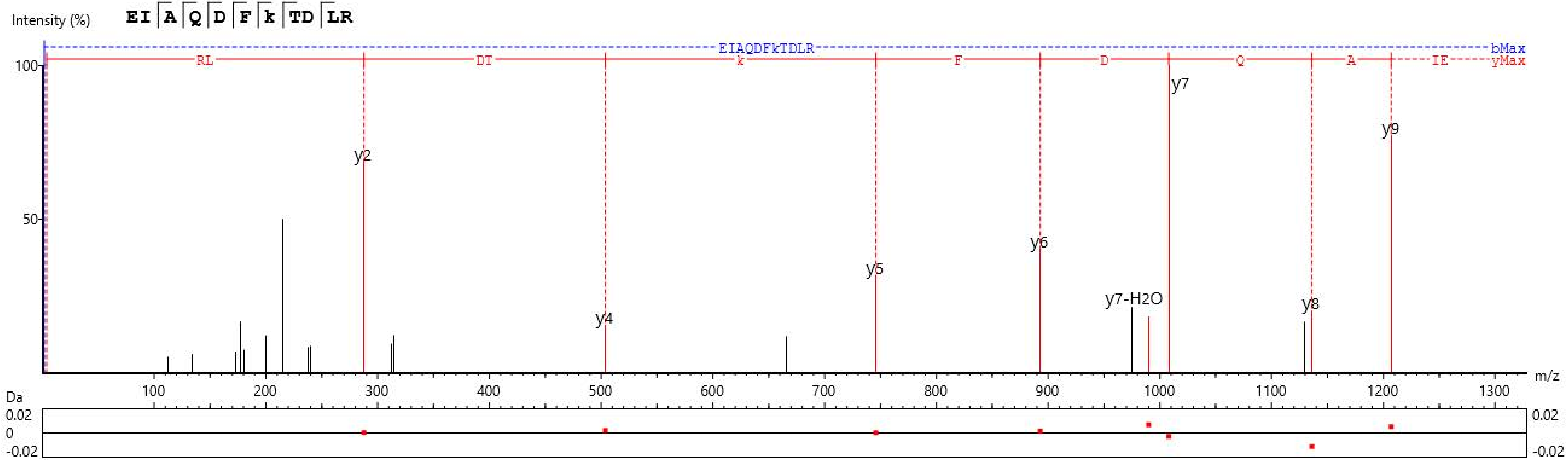

Intensity (%)
EI A Q D F k TD LR
EIAQDFkTDLR
bMax
RL
DT
k
F
D
Q
A
IE
yMax
y2
y4
y5
y6
y7-H2O
y7
y8
y9
m/z
Da

Sample 11 | CL_09.raw | Feature Id: 26464, m/z: 725.3706, RT: 40.74, CV:-40.0, z: 2, -10lgP: 53.34

Scan 15837, m/z=725.3705, z=2, RT=40.64, CV=-40.0, Length=11, -10lgP=53.34, ppm=0.5   1/6

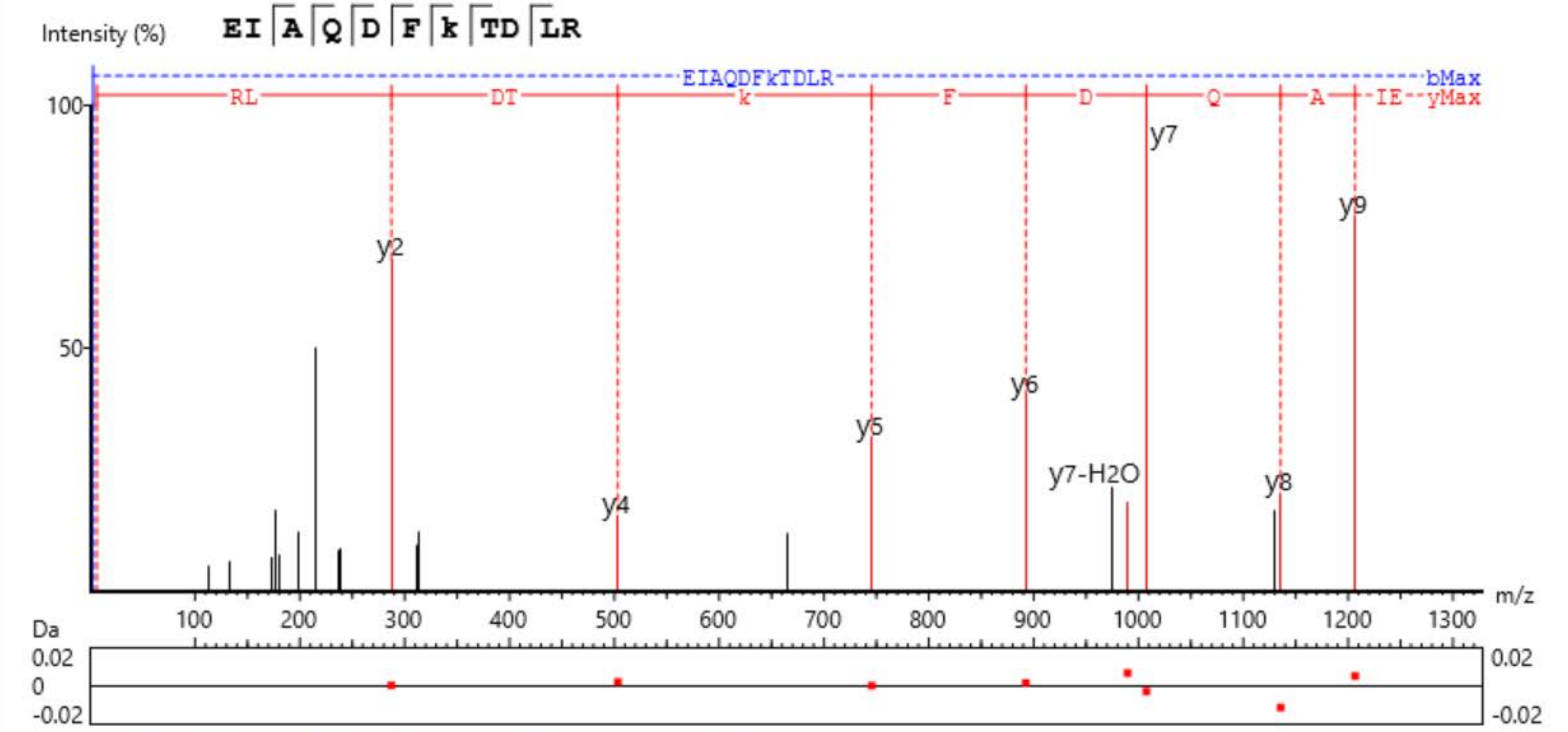

alignment   error map

Ion Match | Survey

#	b	b-H2O	b-NH3	b(2+)	Seq	y	y-H2O	y-NH3	y(2+)	#
1	130.05	112.04	113.02	65.53	E					11
2	243.13	225.12	226.11	122.07	I	1320.69	1302.68	1303.66	660.85	10
3	314.17	296.16	297.14	157.59	A	1207.60	1189.60	1190.58	604.30	9
4	442.23	424.22	425.20	221.62	Q	1136.58	1118.56	1119.54	568.78	8
5	557.26	539.25	540.23	279.13	D	1008.51	990.49	991.48	504.76	7
6	704.33	686.31	687.30	352.66	F	893.48	875.47	876.46	447.24	6
7	946.46	928.45	929.44	473.73	K(+114.04)	746.42	728.40	729.39	373.71	5
8	1047.51	1029.50	1030.48	524.26	T	504.28	486.27	487.25	252.64	4
9	1162.54	1144.53	1145.51	581.77	D	403.23	385.22	386.20	202.11	3
10	1275.62	1257.61	1258.60	638.31	L	288.20	270.19	271.18	144.60	2
11					R	175.12	157.11	158.09	88.06	1

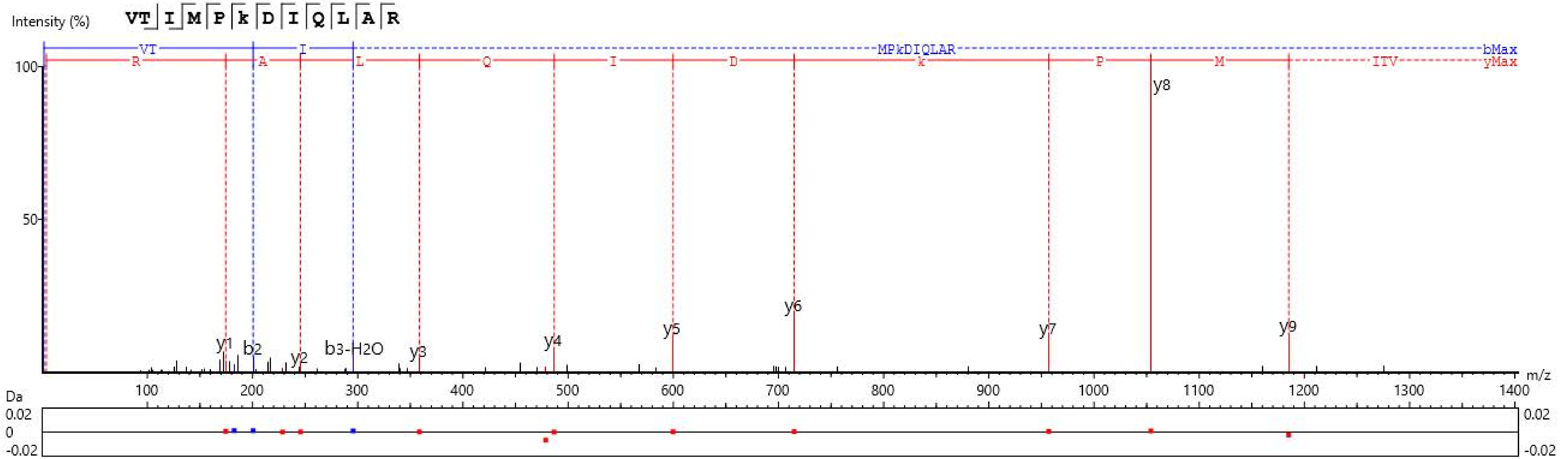
Intensity (%)
VT I M P k D I Q L A R
VT
I
MPkDIQLAR
bMax
R
A
L
Q
I
D
k
P
M
ITV
yMax
100
50
y8
y6
y5
y7
y9
y4
y1
b2
y2
b3-H2O
y3
m/z
100
200
300
400
500
600
700
800
900
1000
1100
1200
1300
1400
Da
0.02
0
-0.02
0.02
-0.02

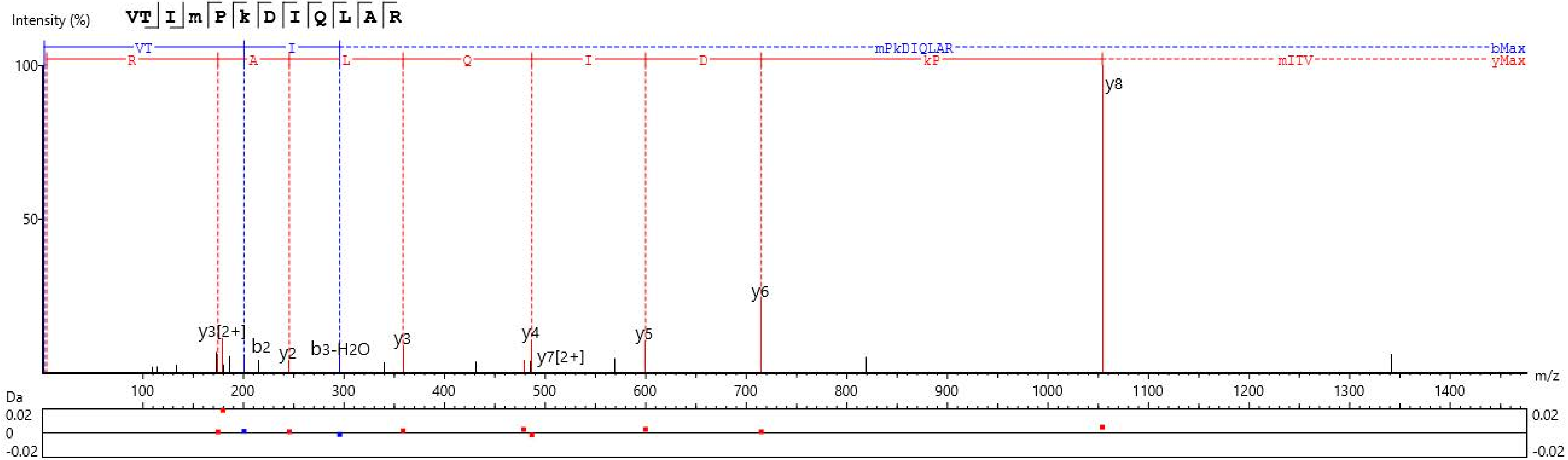

Intensity (%)
VT I m P k D I Q L A R
VT
I
mPkDIQLAR
bMax
R
A
L
Q
I
D
kP
mITV
yMax
100
50
y8
y6
y3[2+]
b2
y2
b3-H2O
y3
y4
y7[2+]
y5
m/z
100
200
300
400
500
600
700
800
900
1000
1100
1200
1300
1400
Da
0.02
0
-0.02

Sample 11 | CL_09.raw | Feature Id: 5695, m/z: 500.2855, RT: 49.24, CV:-60.0, z: 3, -10lgP: 52.82

Scan 21309, m/z=500.2852, z=3, RT=49.15, CV=-60.0, Length=12, -10lgP=52.82, ppm=0.6 1/2

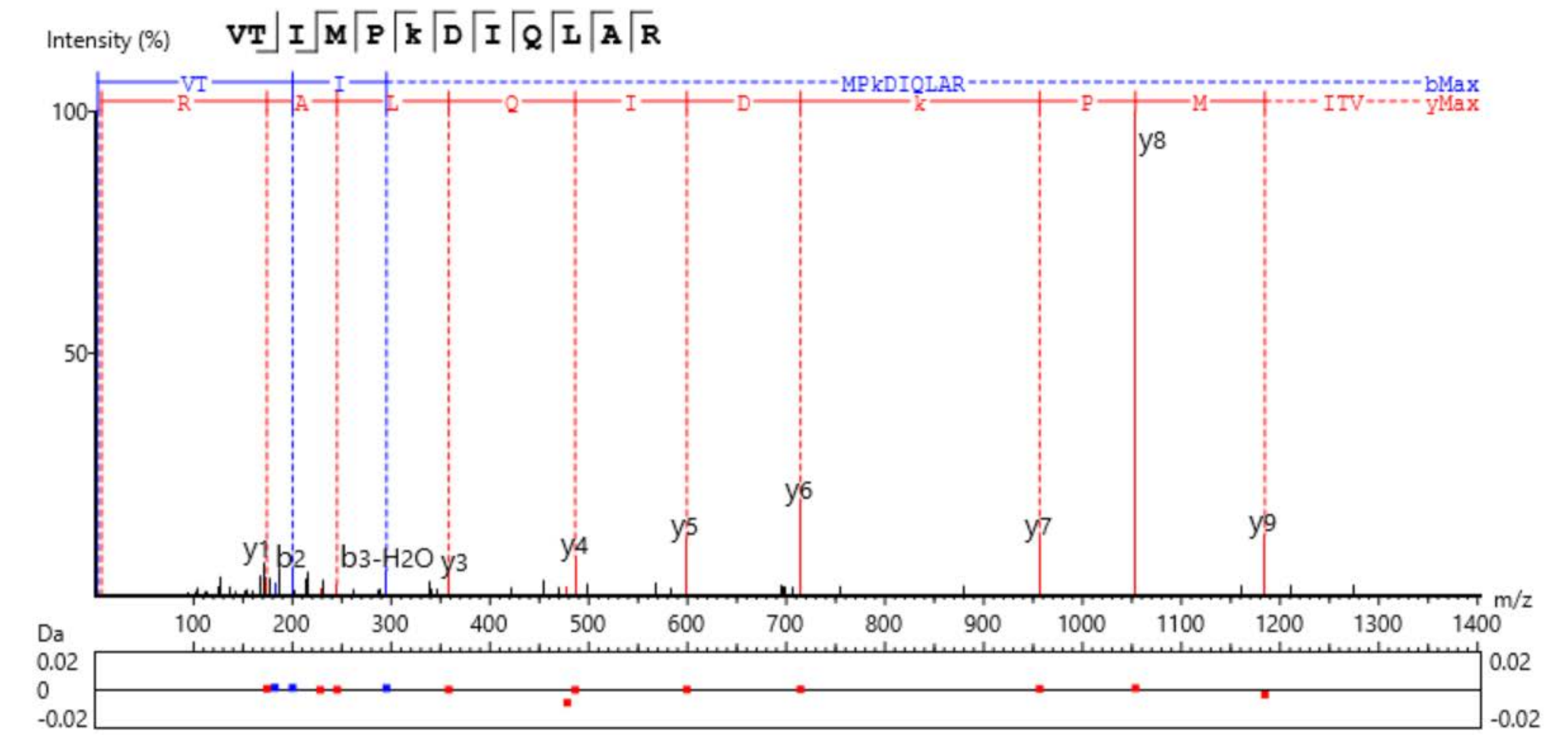

alignment error map

Ion Match | Survey

#	b	b-H2O	b-NH3	b(2+)	Seq	y	y-H2O	y-NH3	y(2+)	#
1	100.08	82.07	83.05	50.54	V					12
2	201.12	183.11	184.10	101.06	T	1399.77	1381.76	1382.75	700.39	11
3	314.21	296.20	297.18	157.60	I	1298.72	1280.71	1281.70	649.86	10
4	445.25	427.24	428.22	223.12	M	1185.64	1167.63	1168.61	593.32	9
5	542.30	524.29	525.27	271.65	P	1054.60	1036.59	1037.57	527.80	8
6	784.44	766.43	767.41	392.72	K(+114.04)	957.55	939.54	940.52	479.28	7
7	899.47	881.46	882.44	450.23	D	715.41	697.40	698.38	358.20	6
8	1012.55	994.54	995.52	506.78	I	600.38	582.37	583.36	300.69	5
9	1140.61	1122.60	1123.58	570.80	Q	487.30	469.29	470.27	244.15	4
10	1253.69	1235.68	1236.67	627.35	L	359.24	341.23	342.21	180.12	3
11	1324.73	1306.72	1307.70	662.86	A	246.16	228.15	229.13	123.58	2
12					R	175.12	157.11	158.09	88.06	1

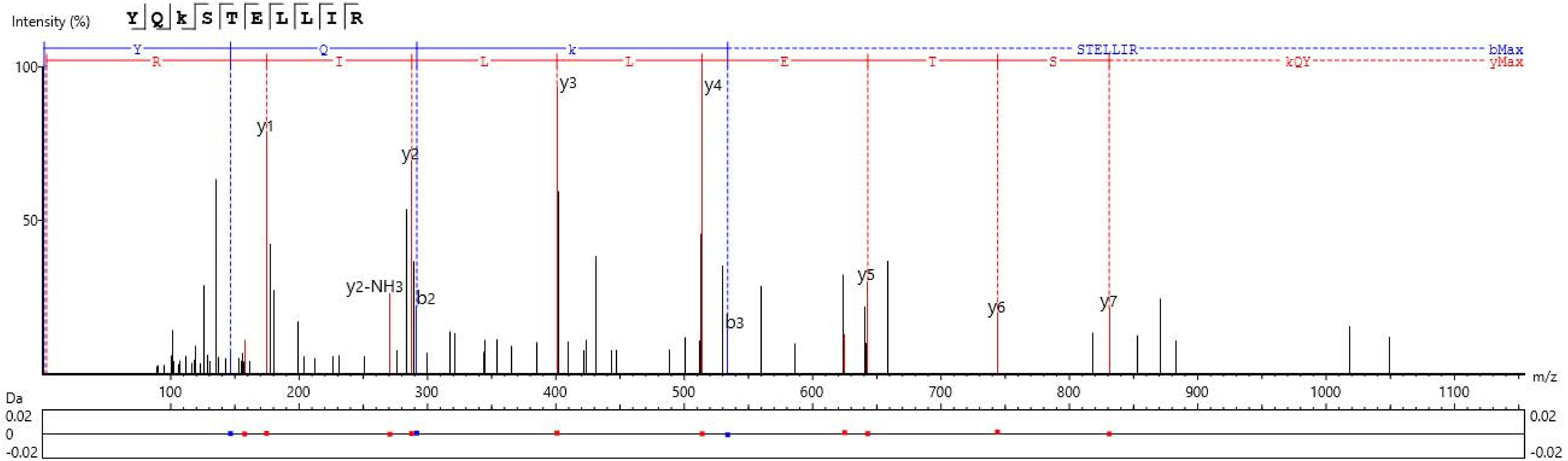

Intensity (%)
Y Q k S T E L L I R
Y
Q
k
STELLIR
bMax
R
I
L
L
E
T
S
kQY
yMax
100
50
y1
y2
y3
y4
y2-NH3
b2
b3
y5
y6
y7
100
200
300
400
500
600
700
800
900
1000
1100
m/z
Da
0.02
0
-0.02

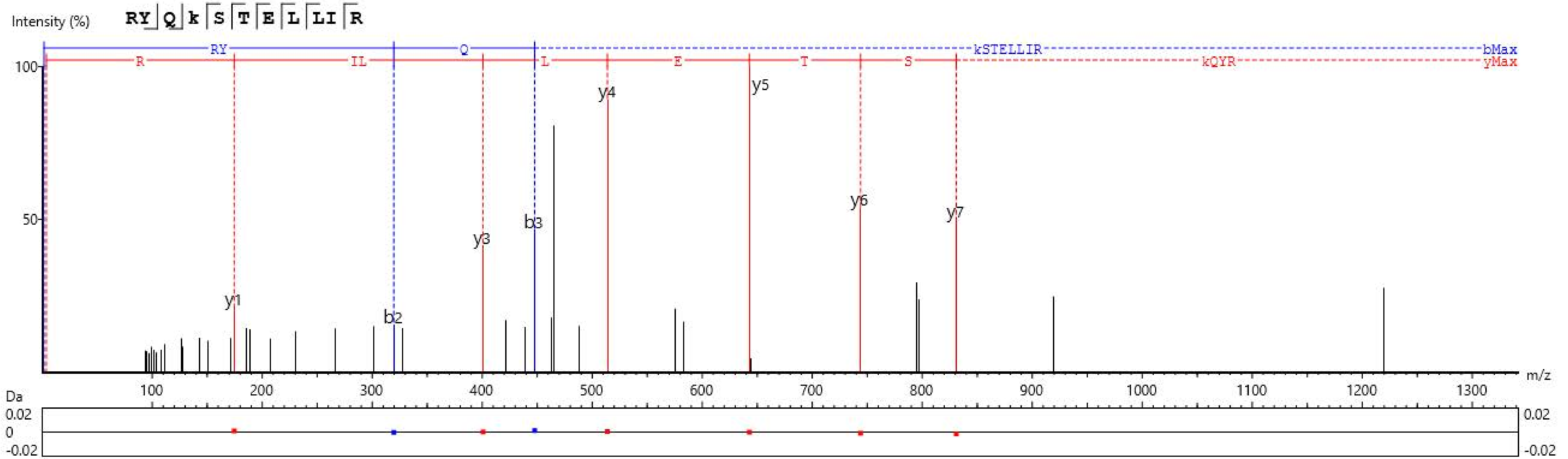

Intensity (%)
RY Q k S T E L LI R
RY
Q
kSTELLIR
bMax
R
IL
L
E
T
S
kQYR
yMax
100
50
y1
b2
y3
b3
y4
y5
y6
y7
m/z
100
200
300
400
500
600
700
800
900
1000
1100
1200
1300
Da
0.02
0
-0.02
0.02
-0.02

Sample 11 | CL_09.raw | Feature Id: 8653, m/z: 571.8357, RT: 14.68, CV:-60.0, z: 2, -10lgP: 22.72

Scan 2489, m/z=571.8358, z=2, RT=14.78, CV=-60.0, Length=9, -10lgP=19.39, ppm=0.1   3/3

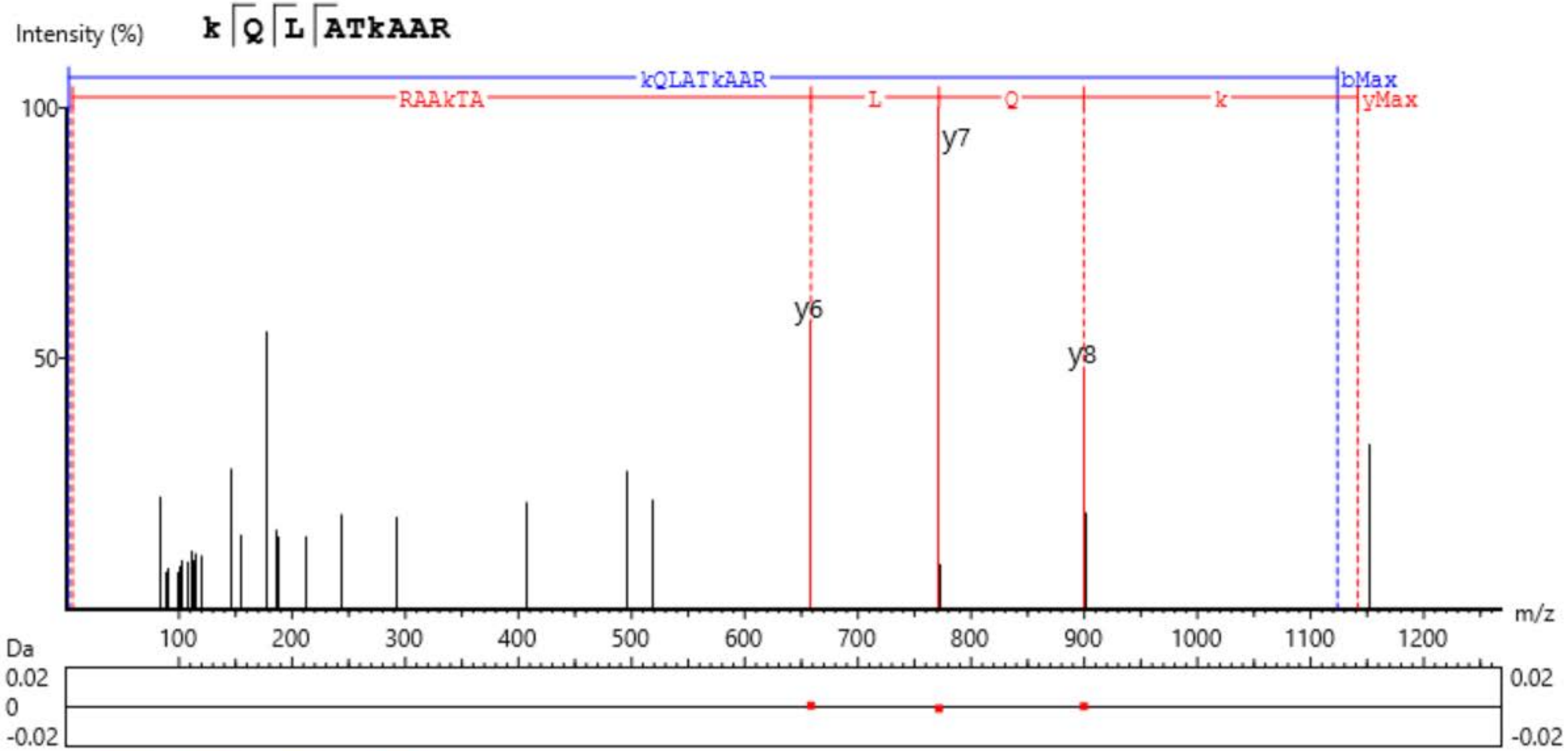

alignment   error map

Ion Match | Survey

#	b	b-H2O	b-NH3	b(2+)	Seq	y	y-H2O	y-NH3	y(2+)	#
1	243.15	225.14	226.12	122.07	K(+114.04)					9
2	371.20	353.19	354.18	186.10	Q	900.53	882.52	883.50	450.76	8
3	484.29	466.28	467.26	242.64	L	772.47	754.46	755.44	386.73	7
4	555.33	537.31	538.30	278.16	A	659.38	641.37	642.36	330.19	6
5	656.37	638.36	639.35	328.69	T	588.35	570.34	571.32	294.67	5
6	826.48	808.47	809.45	413.74	K(+42.01)	487.30	469.29	470.27	244.15	4
7	897.52	879.51	880.49	449.26	A	317.19	299.18	300.17	159.10	3
8	968.55	950.54	951.53	484.78	A	246.16	228.15	229.13	123.58	2
9					R	175.12	157.11	158.09	88.06	1

Sample 11 | CL_09.raw | Feature Id: 5969, m/z: 507.6230, RT: 34.50, CV:-60.0, z: 3, -10lgP: 57.63

Scan 12264, m/z=507.6238, z=3, RT=34.60, CV=-60.0, Length=11, -10lgP=57.63, ppm=0.0   2/5

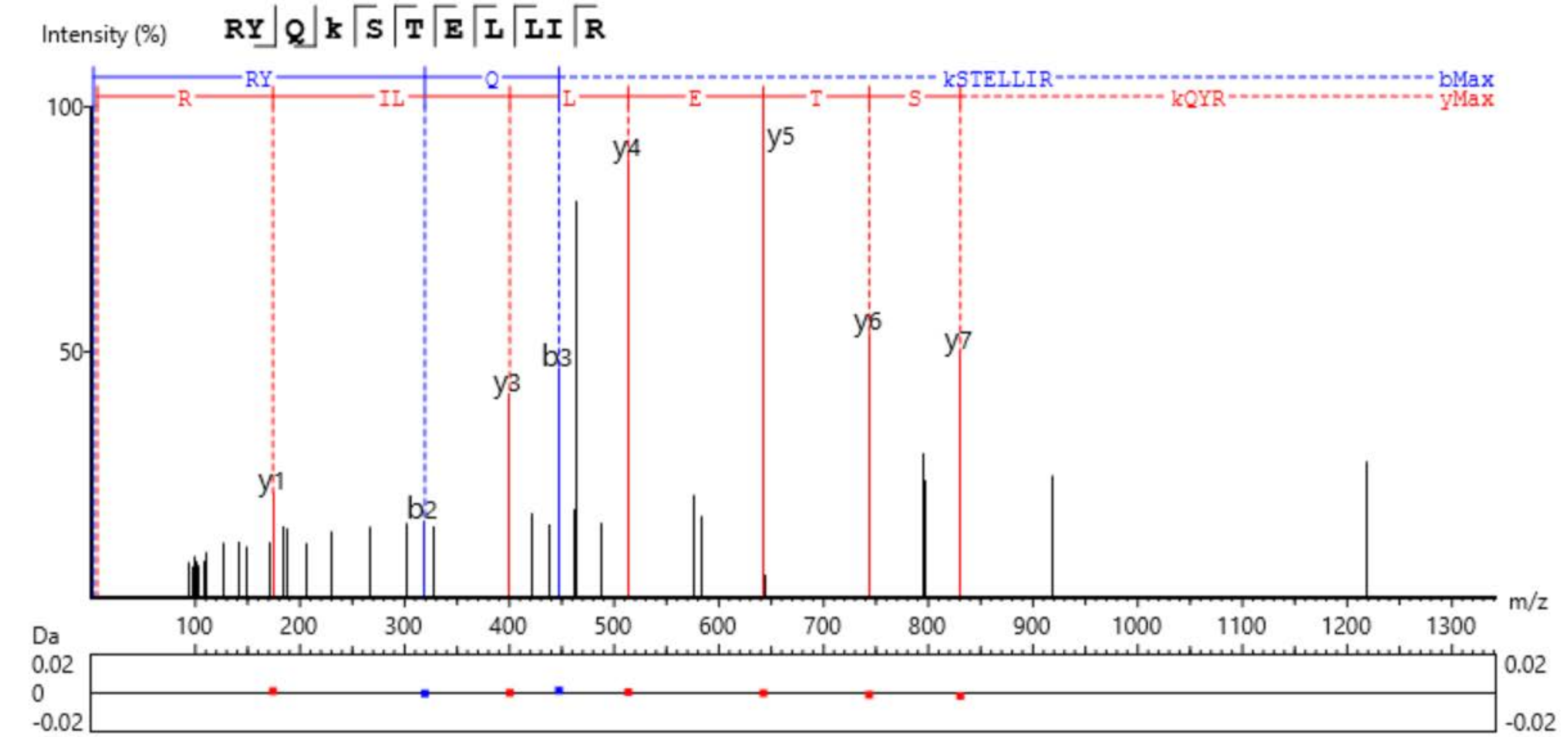

alignment   error map

Ion Match | Survey

#	b	b-H2O	b-NH3	b(2+)	Seq	y	y-H2O	y-NH3	y(2+)	#
1	157.11	139.10	140.08	79.05	R					11
2	320.17	302.16	303.15	160.59	Y	1364.75	1346.74	1347.73	682.88	10
3	448.23	430.22	431.20	224.62	Q	1201.69	1183.68	1184.66	601.34	9
4	690.37	672.36	673.34	345.68	K(+114.04)	1073.63	1055.62	1056.60	537.32	8
5	777.40	759.39	760.37	389.20	S	831.50	813.48	814.47	416.25	7
6	878.45	860.44	861.42	439.72	T	744.46	726.45	727.43	372.73	6
7	1007.49	989.48	990.46	504.25	E	643.41	625.40	626.39	322.21	5
8	1120.58	1102.56	1103.55	560.79	L	514.37	496.36	497.34	257.69	4
9	1233.66	1215.65	1216.63	617.33	L	401.29	383.28	384.26	201.14	3
10	1346.74	1328.73	1329.72	673.87	I	288.20	270.19	271.18	144.60	2
11					R	175.12	157.11	158.09	88.06	1

Sample 11 | CL_09.raw | Feature Id: 4031, m/z: 455.5894, RT: 37.58, CV:-60.0, z: 3, -10lgP: 36.77

Scan 14054, m/z=455.5898, z=3, RT=37.53, CV=-60.0, Length=10, -10lgP=36.77, ppm=0.4   1/2

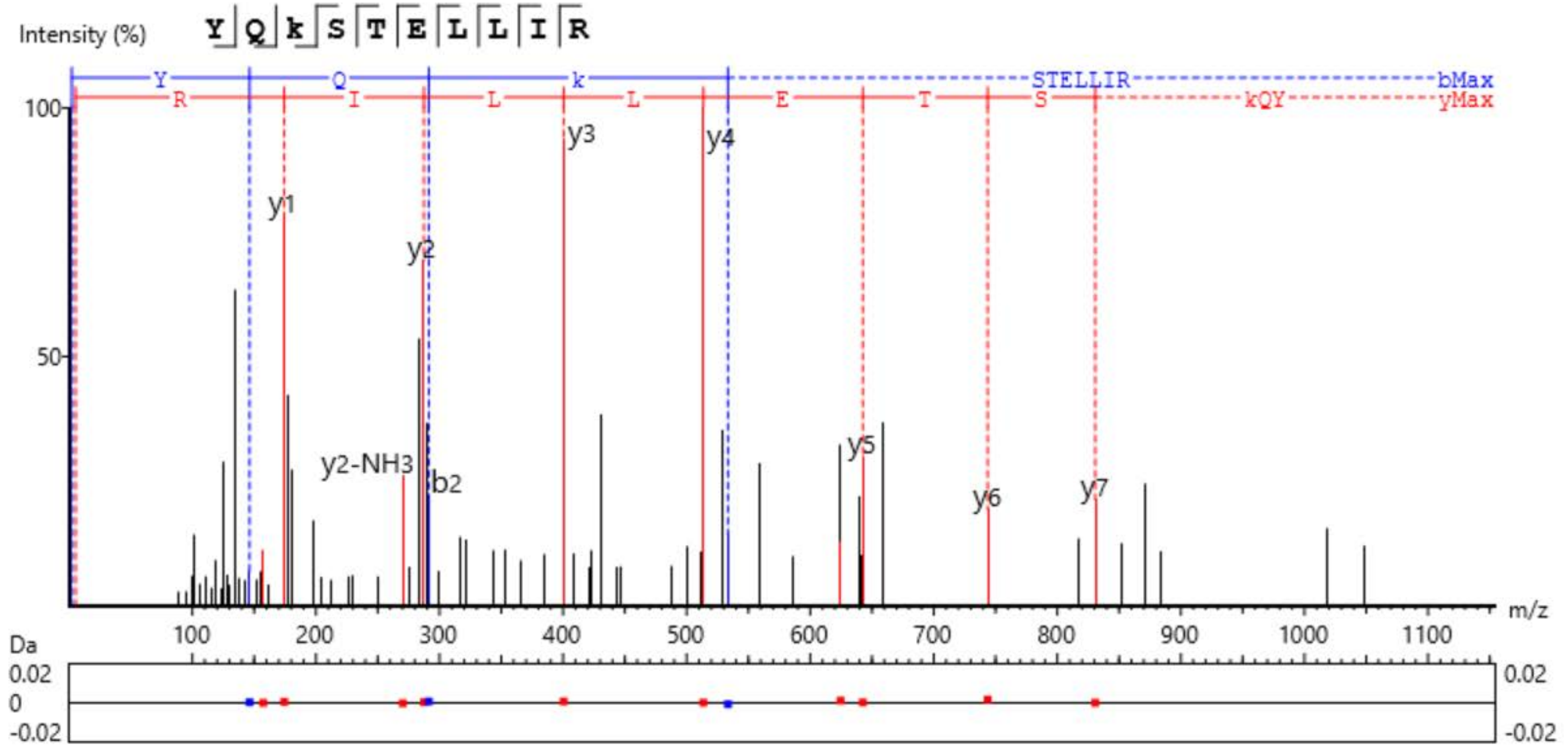

alignment   error map

Ion Match | Survey

#	b	b-H2O	b-NH3	b(2+)	Seq	y	y-H2O	y-NH3	y(2+)	#
1	164.07	146.06	147.04	82.54	Y					10
2	292.13	274.12	275.10	146.56	Q	1201.69	1183.68	1184.66	601.34	9
3	534.27	516.26	517.24	267.63	K(+114.04)	1073.63	1055.62	1056.60	537.32	8
4	621.30	603.29	604.27	311.15	S	831.49	813.48	814.47	416.25	7
5	722.35	704.34	705.32	361.67	T	744.46	726.45	727.43	372.73	6
6	851.39	833.38	834.36	426.19	E	643.41	625.40	626.39	322.21	5
7	964.47	946.46	947.45	482.74	L	514.37	496.36	497.34	257.69	4
8	1077.56	1059.55	1060.53	539.28	L	401.29	383.28	384.26	201.14	3
9	1190.64	1172.63	1173.62	595.82	I	288.20	270.19	271.18	144.60	2
10					R	175.12	157.11	158.09	88.06	1

Sample 11 | CL_09.raw | Feature Id: 19356, m/z: 505.6172, RT: 42.46, CV:-40.0, z: 3, -10lgP: 48.91

Scan 16992, m/z=505.6175, z=3, RT=42.39, CV=-40.0, Length=12, -10lgP=48.91, ppm=0.7 1/3

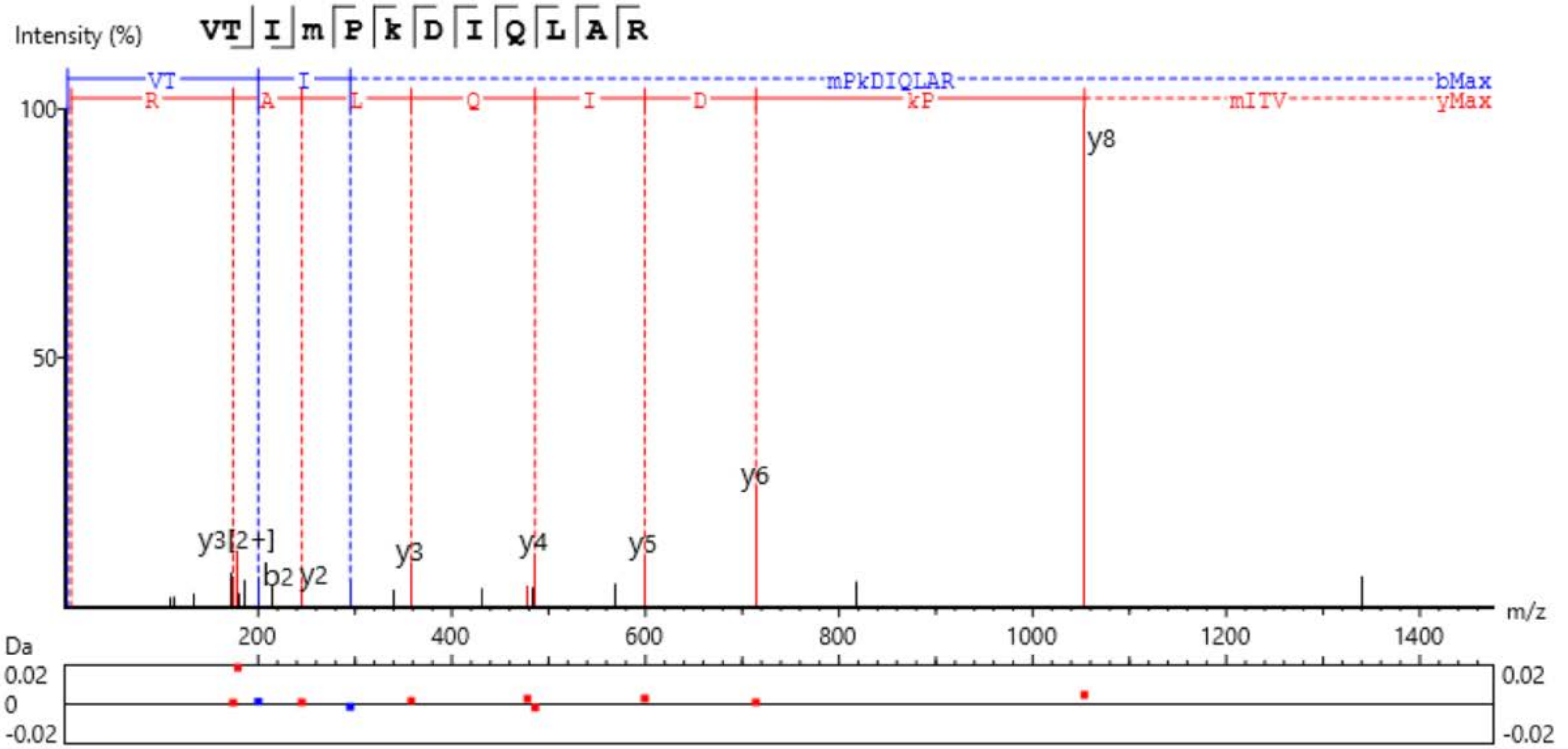

alignment error map

Ion Match | Survey

#	b	b-H2O	b-NH3	b(2+)	Seq	y	y-H2O	y-NH3	y(2+)	#
1	100.08	82.07	83.05	50.54	V					12
2	201.12	183.11	184.10	101.06	T	1415.77	1397.76	1398.74	708.38	11
3	314.21	296.20	297.18	157.60	I	1314.72	1296.71	1297.69	657.86	10
4	461.24	443.23	444.22	231.12	M(+15.99)	1201.64	1183.63	1184.61	601.32	9
5	558.30	540.29	541.27	279.65	P	1054.60	1036.59	1037.57	527.80	8
6	800.43	782.42	783.41	400.72	K(+114.04)	957.55	939.54	940.52	479.27	7
7	915.46	897.45	898.43	458.23	D	715.41	697.40	698.38	358.20	6
8	1028.55	1010.53	1011.52	514.77	I	600.38	582.37	583.36	300.69	5
9	1156.60	1138.59	1139.58	578.80	Q	487.30	469.29	470.27	244.15	4
10	1269.69	1251.68	1252.66	635.34	L	359.24	341.23	342.21	180.10	3
11	1340.72	1322.71	1323.70	670.86	A	246.16	228.15	229.13	123.58	2
12					R	175.12	157.11	158.09	88.06	1

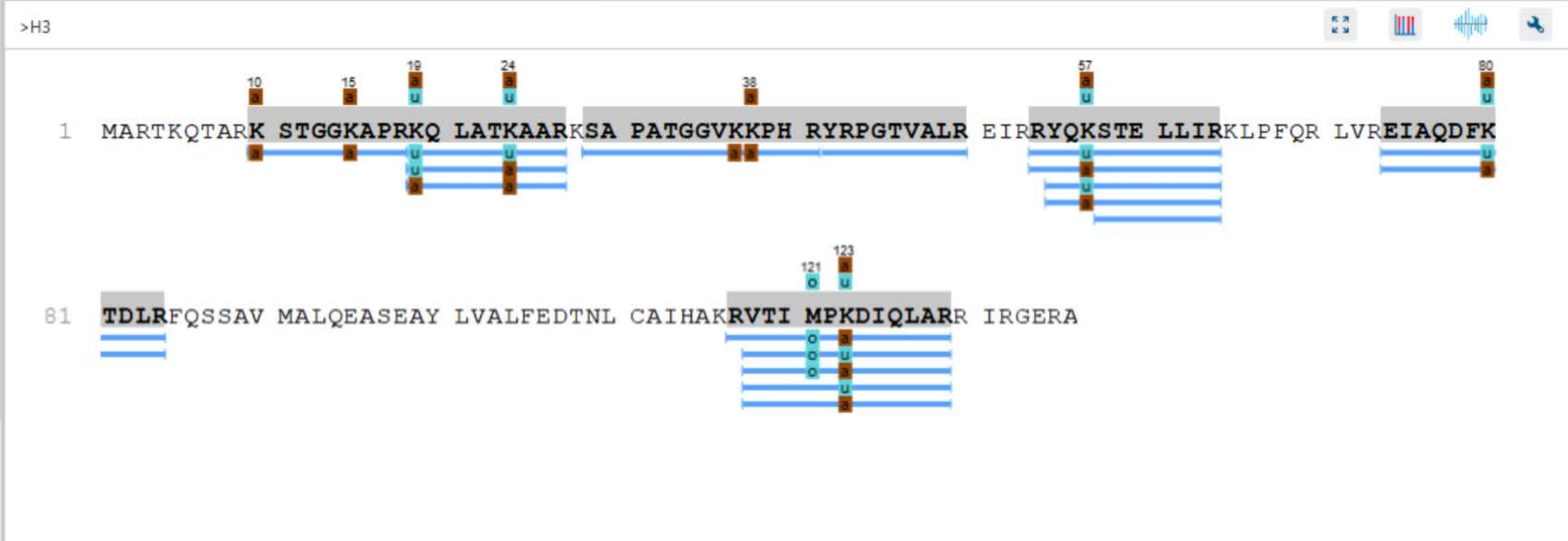
>H3
10
15
19
24
38
57
80
1 MARTKQTARK STGGKAPRKQ LATKAARKSA PATGGVKKPH RYRPGTVALR EIRRYQKSTE LLIRKLPFQR LVREIAQDFK
121
123
81 TDLRFQSSAV MALQEASEAY LVALFEDTNL CAIHAKRVTI MPKDIQLARR IRGERA

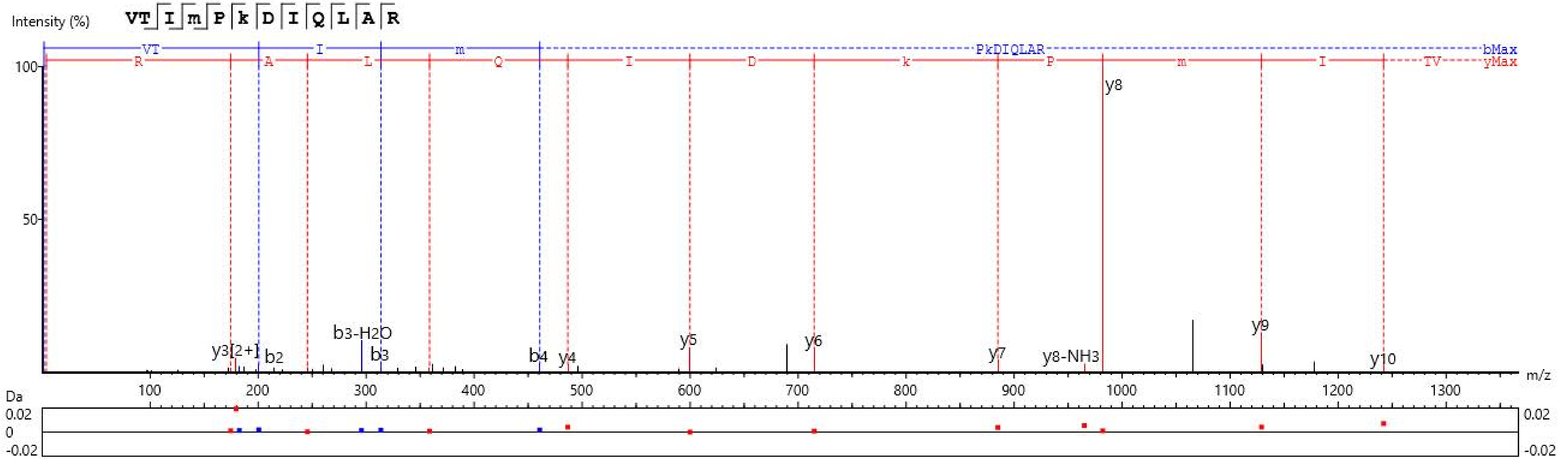

Intensity (%)
VTImPkDIQLAR
bMax
yMax
y8
y9
y3[2+]
b2
b3-H2O
b3
b4
y4
y5
y6
y7
y8-NH3
y10
m/z
Da

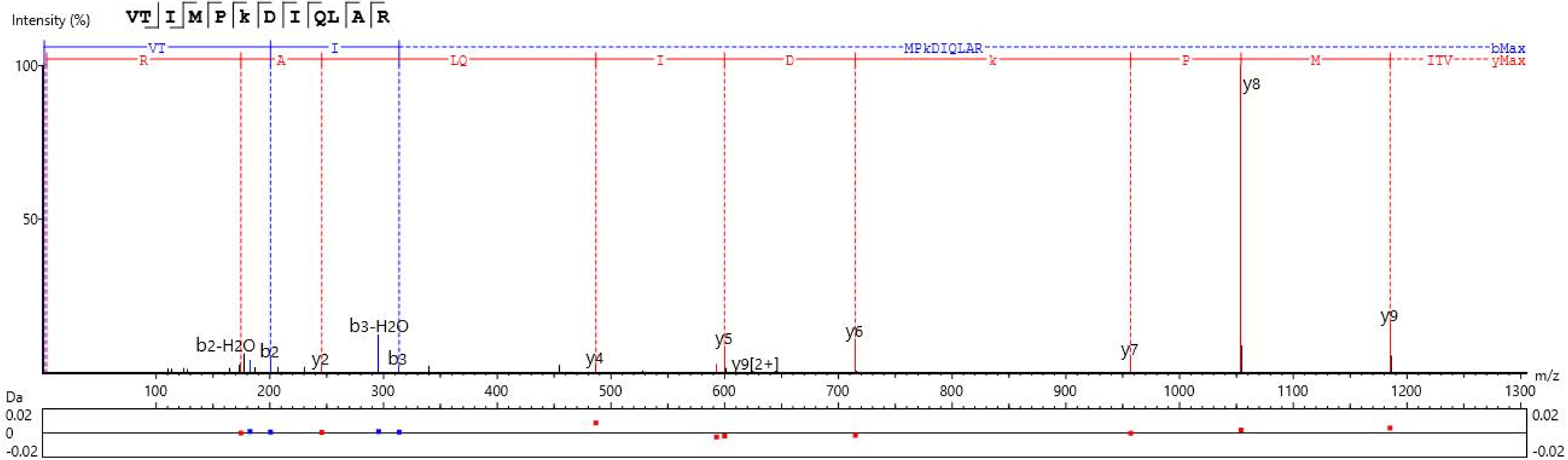

Intensity (%)
VT I M P k D I QL A R
VT
I
MPkDIQLAR
bMax
R
A
LQ
I
D
k
P
M
ITV
yMax
100
50
y8
y9
b3-H2O
b2-H2O
b2
y2
b3
y4
y5
y9[2+]
y6
y7
m/z
100
200
300
400
500
600
700
800
900
1000
1100
1200
1300
Da
0.02
0
-0.02

Sample 2 | CL_010.raw | Feature Id: 3339, m/z: 405.5706, RT: 10.79, CV:-60.0, z: 3, -10lgP: 37.77

Scan 1251, m/z=405.5703, z=3, RT=10.97, CV=-60.0, Length=9, -10lgP=37.77, ppm=0.8   4/5

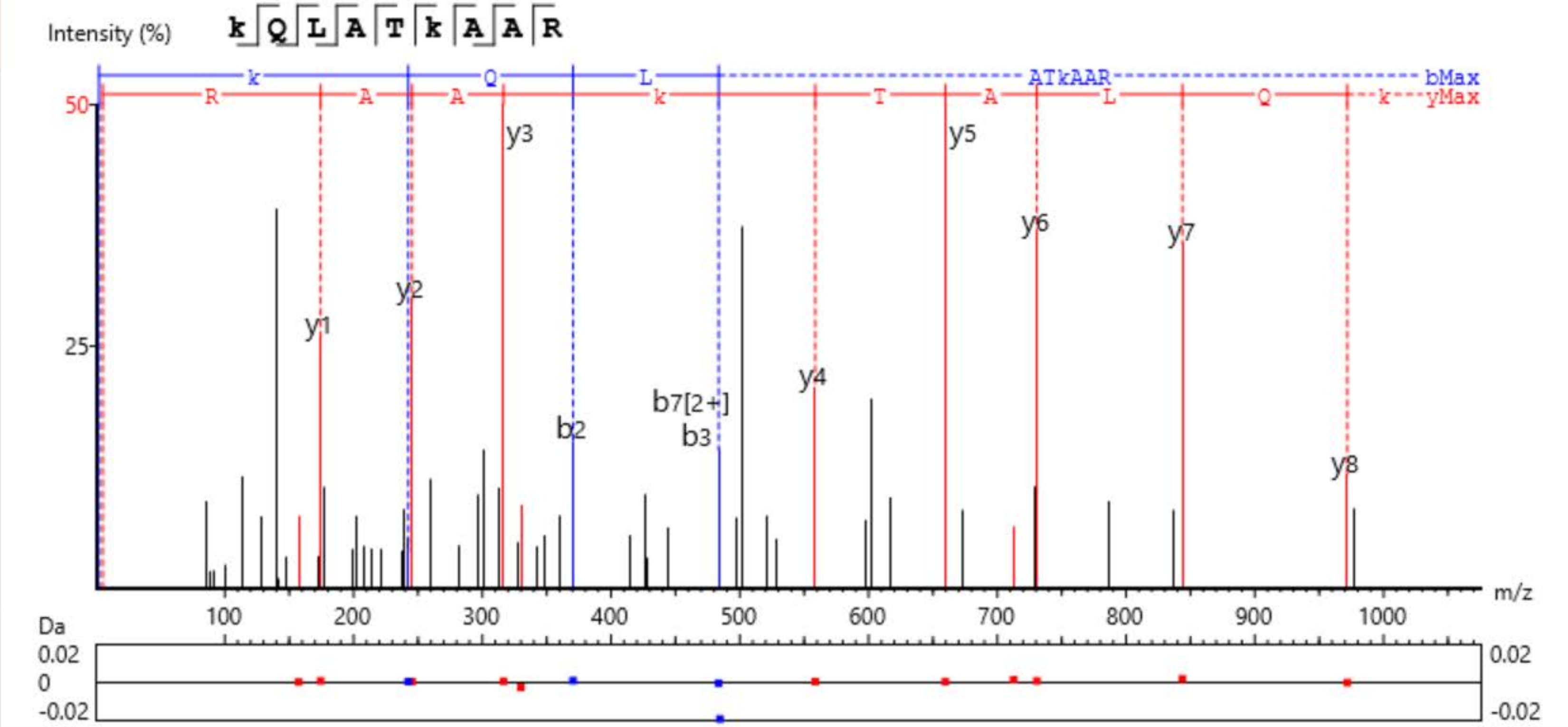

1:1 2X 2Y alignment error map

Ion Match | Survey

#	b	b-H2O	b-NH3	b(2+)	Seq	y	y-H2O	y-NH3	y(2+)	#
1	243.15	225.14	226.12	122.07	K(+114.04)					9
2	371.20	353.19	354.18	186.10	Q	972.56	954.55	955.53	486.78	8
3	484.29	466.28	467.26	242.64	L	844.50	826.49	827.47	422.75	7
4	555.33	537.31	538.30	278.16	A	731.42	713.40	714.39	366.21	6
5	656.37	638.36	639.35	328.69	T	660.38	642.37	643.35	330.69	5
6	898.51	880.50	881.48	449.76	K(+114.04)	559.33	541.32	542.30	280.17	4
7	969.55	951.54	952.52	485.29	A	317.19	299.18	300.17	159.10	3
8	1040.59	1022.57	1023.56	520.79	A	246.16	228.15	229.13	123.58	2
9					R	175.12	157.11	158.09	88.06	1

Sample 2 | CL_010.raw | Feature Id: 26398, m/z: 721.9053, RT: 49.49, CV:-40.0, z: 2, -10lgP: 65.77

Scan 23615, m/z=721.9072, z=2, RT=50.16, CV=-40.0, Length=12, -10lgP=65.77, ppm=-0.2   9/34

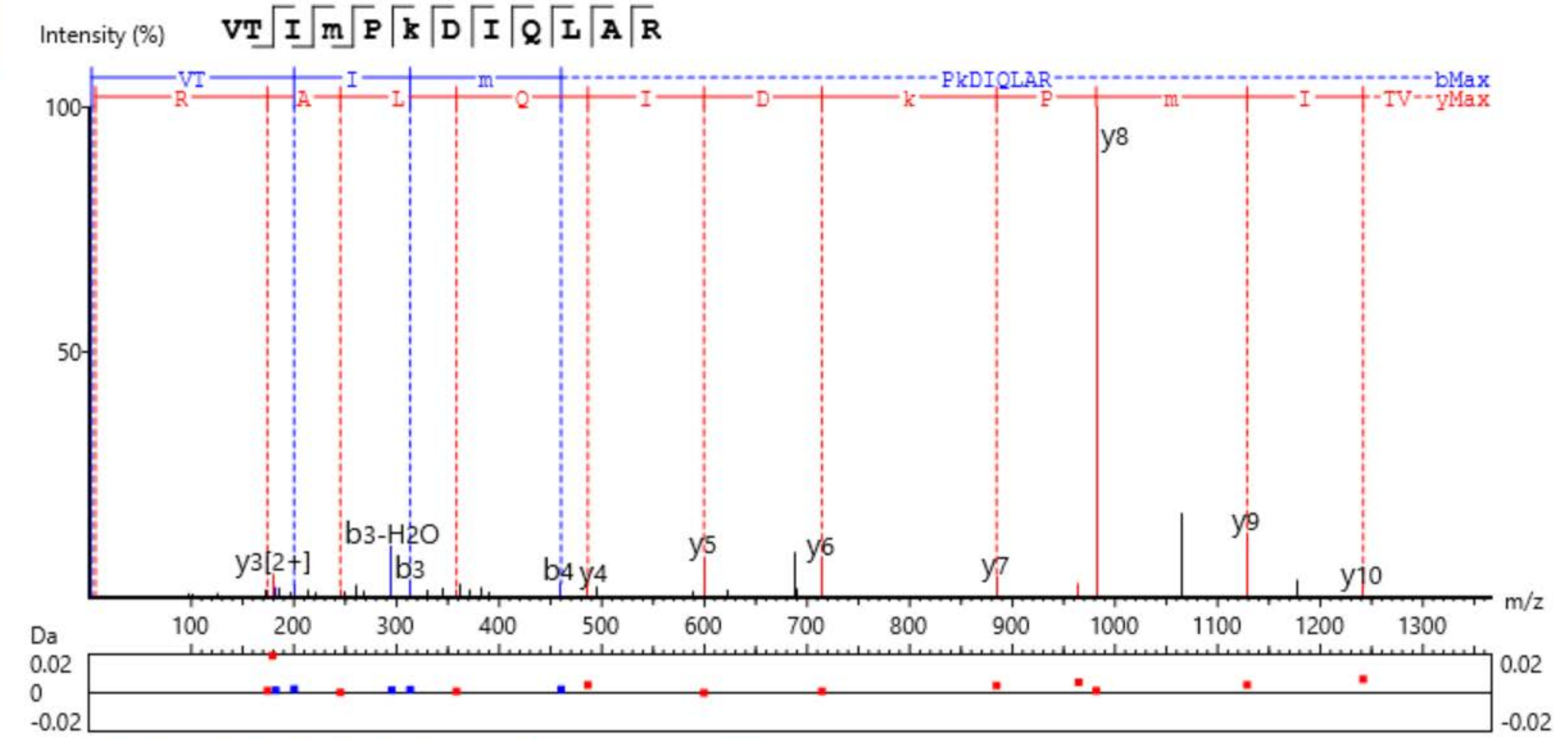

1:1 2X 2Y ✓ alignment ✓ error map

Ion Match | Survey

#	b	b-H2O	b-NH3	b(2+)	Seq	y	y-H2O	y-NH3	y(2+)	#
1	100.08	82.07	83.05	50.54	V					12
2	201.12	183.11	184.10	101.06	T	1343.74	1325.72	1326.71	672.37	11
3	314.21	296.20	297.18	157.60	I	1242.68	1224.68	1225.66	621.84	10
4	461.24	443.23	444.22	231.12	M(+15.99)	1129.60	1111.59	1112.58	565.30	9
5	558.30	540.29	541.27	279.65	P	982.57	964.56	965.54	491.78	8
6	728.40	710.39	711.37	364.70	K(+42.01)	885.51	867.50	868.49	443.26	7
7	843.43	825.42	826.40	422.21	D	715.41	697.40	698.38	358.20	6
8	956.51	938.50	939.49	478.76	I	600.38	582.37	583.36	300.69	5
9	1084.57	1066.56	1067.54	542.79	Q	487.30	469.29	470.27	244.15	4
10	1197.66	1179.64	1180.63	599.33	L	359.24	341.23	342.21	180.10	3
11	1268.69	1250.68	1251.67	634.85	A	246.16	228.15	229.13	123.58	2
12					R	175.12	157.11	158.09	88.06	1

Sample 2 | CL_010.raw | Feature Id: 26517, m/z: 725.3719, RT: 40.25, CV:-40.0, z: 2, -10lgP: 58.78

Scan 16881, m/z=725.3717, z=2, RT=40.30, CV=-40.0, Length=11, -10lgP=58.78, ppm=2.4 3/5

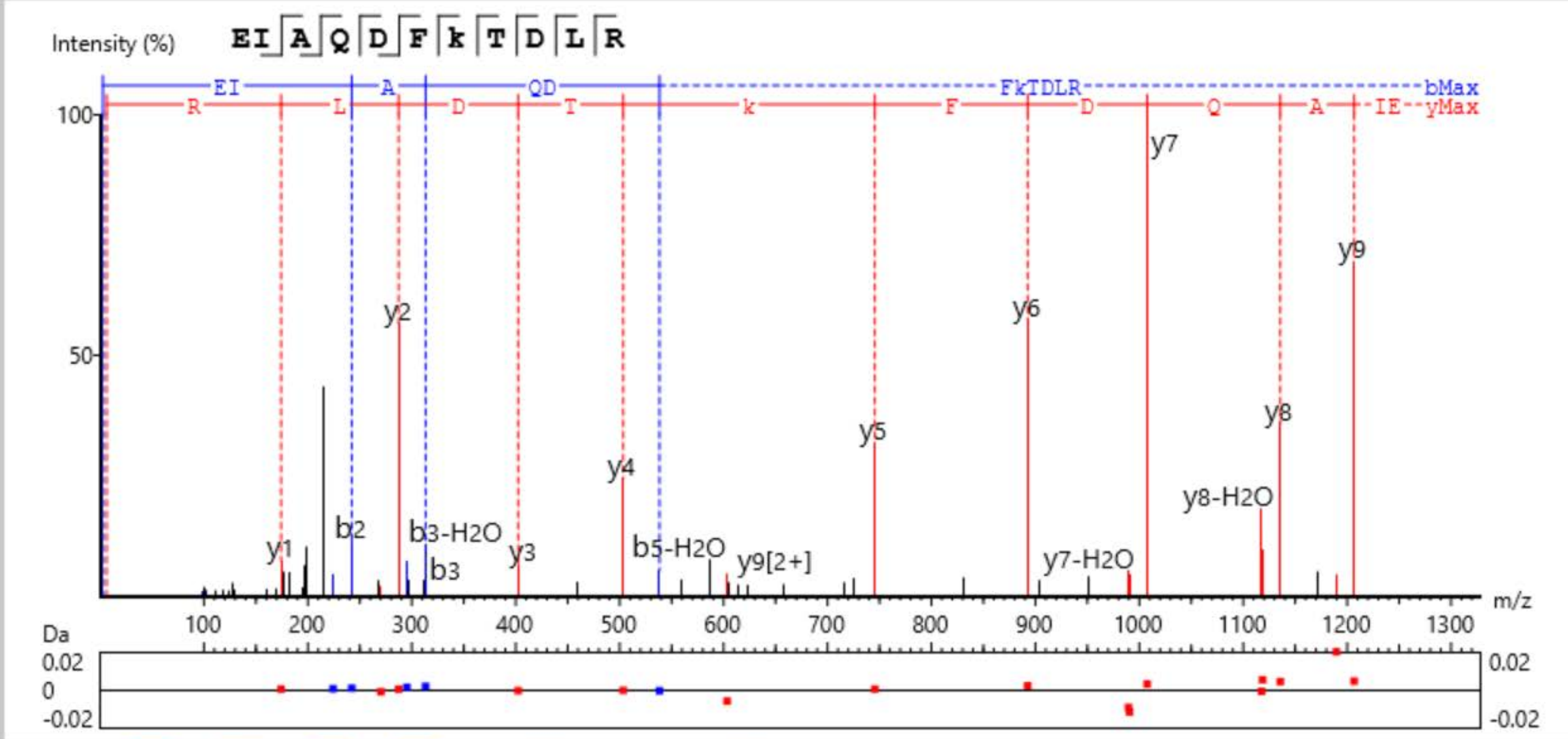

alignment error map

Ion Match | Survey

#	b	b-H2O	b-NH3	b(2+)	Seq	y	y-H2O	y-NH3	y(2+)	#
1	130.05	112.04	113.02	65.53	E					11
2	243.13	225.12	226.11	122.07	I	1320.69	1302.68	1303.66	660.85	10
3	314.17	296.16	297.14	157.59	A	1207.60	1189.60	1190.56	604.31	9
4	442.23	424.22	425.20	221.62	Q	1136.57	1118.56	1119.54	568.78	8
5	557.26	539.25	540.23	279.13	D	1008.51	990.51	991.50	504.76	7
6	704.33	686.31	687.30	352.66	F	893.48	875.47	876.46	447.24	6
7	946.46	928.45	929.44	473.73	K(+114.04)	746.42	728.40	729.39	373.71	5
8	1047.51	1029.50	1030.48	524.26	T	504.28	486.27	487.25	252.64	4
9	1162.54	1144.53	1145.51	581.77	D	403.23	385.22	386.20	202.11	3
10	1275.62	1257.61	1258.60	638.31	L	288.20	270.19	271.18	144.60	2
11					R	175.12	157.11	158.09	88.06	1

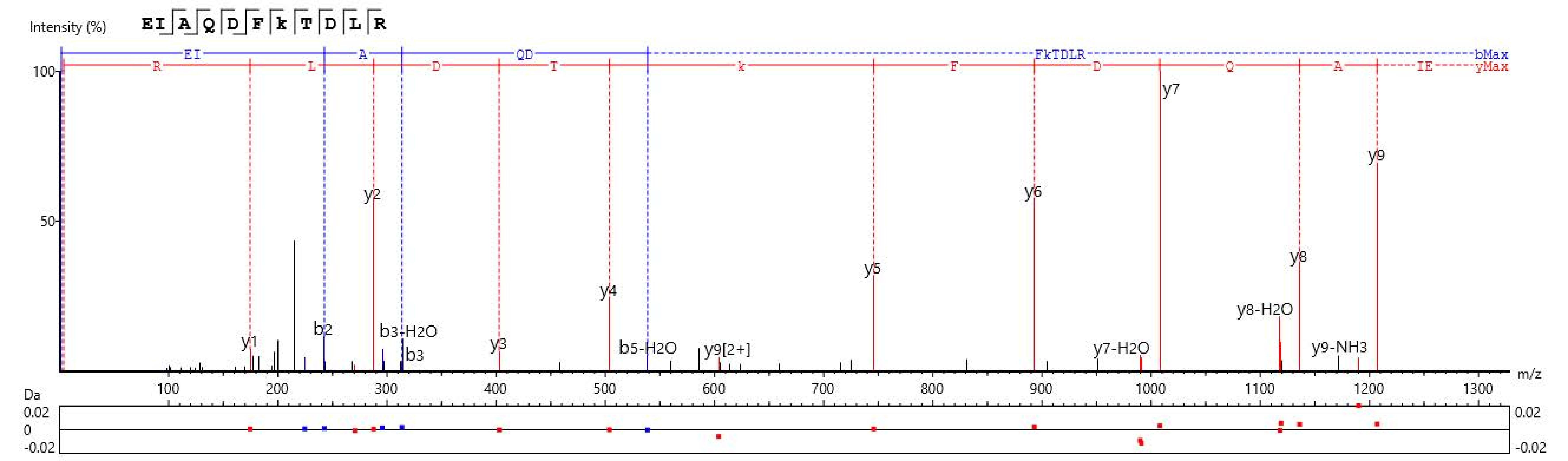

Intensity (%)
EIAQDFkTDLR
EI A QD FkTDLR bMax
R L D T k F D Q A IE yMax
100
50
y1 b2 y2 b3-H2O b3 y3 y4 b5-H2O y9[2+] y5 y6 y7-H2O y7 y8-H2O y8 y9-NH3 y9
100 200 300 400 500 600 700 800 900 1000 1100 1200 1300
m/z
Da
0.02
0
-0.02

Sample 2 | CL_010.raw | Feature Id: 9153, m/z: 571.8361, RT: 14.62, CV:-60.0, z: 2, -10lgP: 22.98

Scan 2289, m/z=571.8357, z=2, RT=14.46, CV=-60.0, Length=9, -10lgP=22.98, ppm=0.7 | 2/3

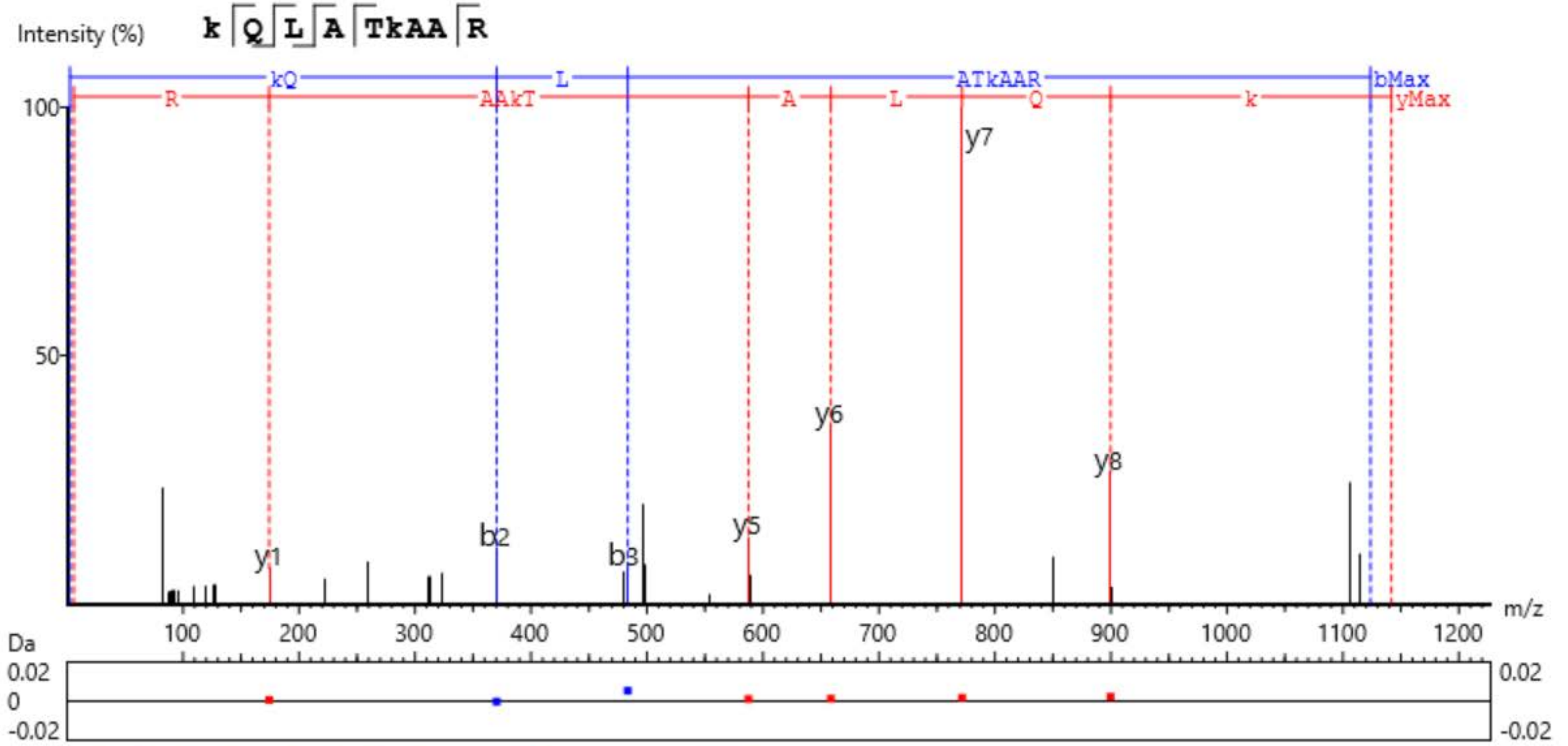

alignment | error map

Ion Match | Survey

#	b	b-H2O	b-NH3	b(2+)	Seq	y	y-H2O	y-NH3	y(2+)	#
1	243.15	225.14	226.12	122.07	K(+114.04)					9
2	371.21	353.19	354.18	186.10	Q	900.52	882.52	883.50	450.76	8
3	484.28	466.28	467.26	242.64	L	772.47	754.46	755.44	386.73	7
4	555.33	537.31	538.30	278.16	A	659.38	641.37	642.36	330.19	6
5	656.37	638.36	639.35	328.69	T	588.35	570.34	571.32	294.67	5
6	826.48	808.47	809.45	413.74	K(+42.01)	487.30	469.29	470.27	244.15	4
7	897.52	879.51	880.49	449.26	A	317.19	299.18	300.17	159.10	3
8	968.55	950.54	951.53	484.78	A	246.16	228.15	229.13	123.58	2
9					R	175.12	157.11	158.09	88.06	1

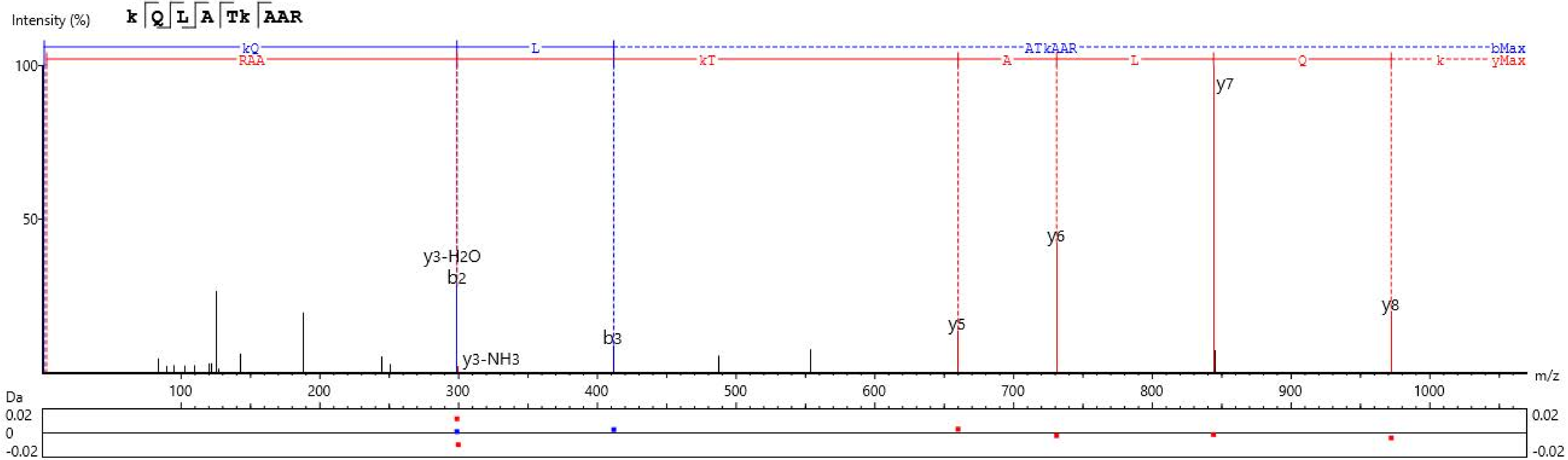
Intensity (%)
k Q L A Tk AAR
kQ
L
ATkAAR
bMax
RAA
kT
A
L
Q
k
yMax
y7
y6
y3-H2O
b2
y8
y5
b3
y3-NH3
m/z
Da
0.02
0
-0.02

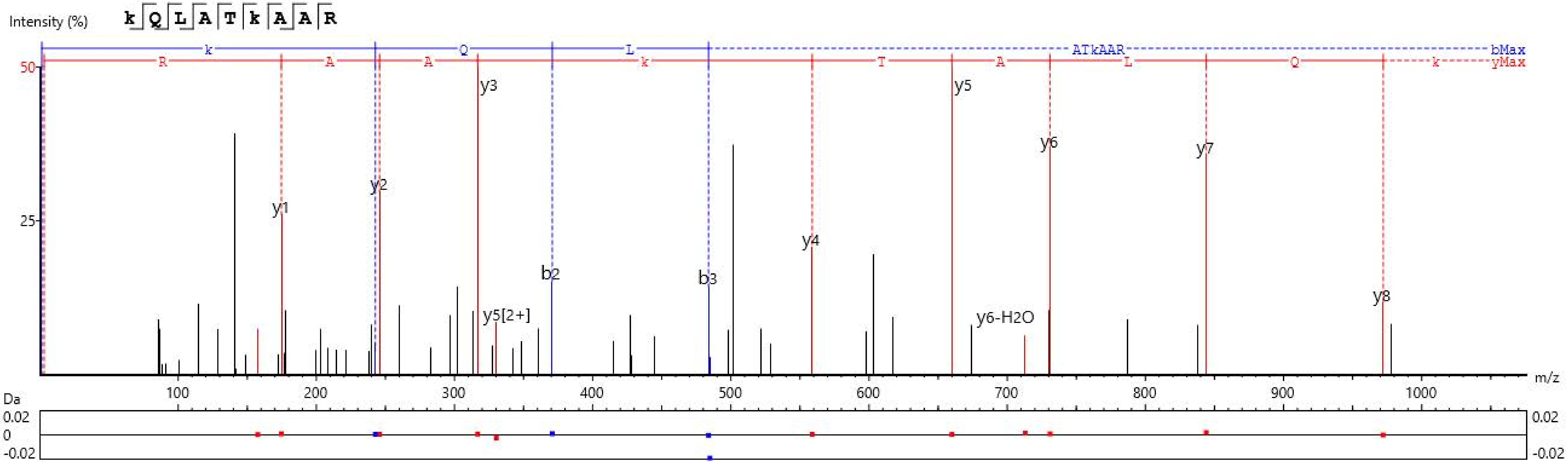

Intensity (%)
kQLATkAAR
bMax
yMax
y1
y2
y3
y4
y5
y6
y7
y8
b2
b3
y5[2+]
y6-H2O
m/z
Da

Sample 2 | CL_010.raw | Feature Id: 27721, m/z: 749.9250, RT: 48.04, CV:-40.0, z: 2, -10lgP: 59.86

Scan 22116, m/z=749.9225, z=2, RT=48.02, CV=-40.0, Length=12, -10lgP=59.86, ppm=1.1   1/2

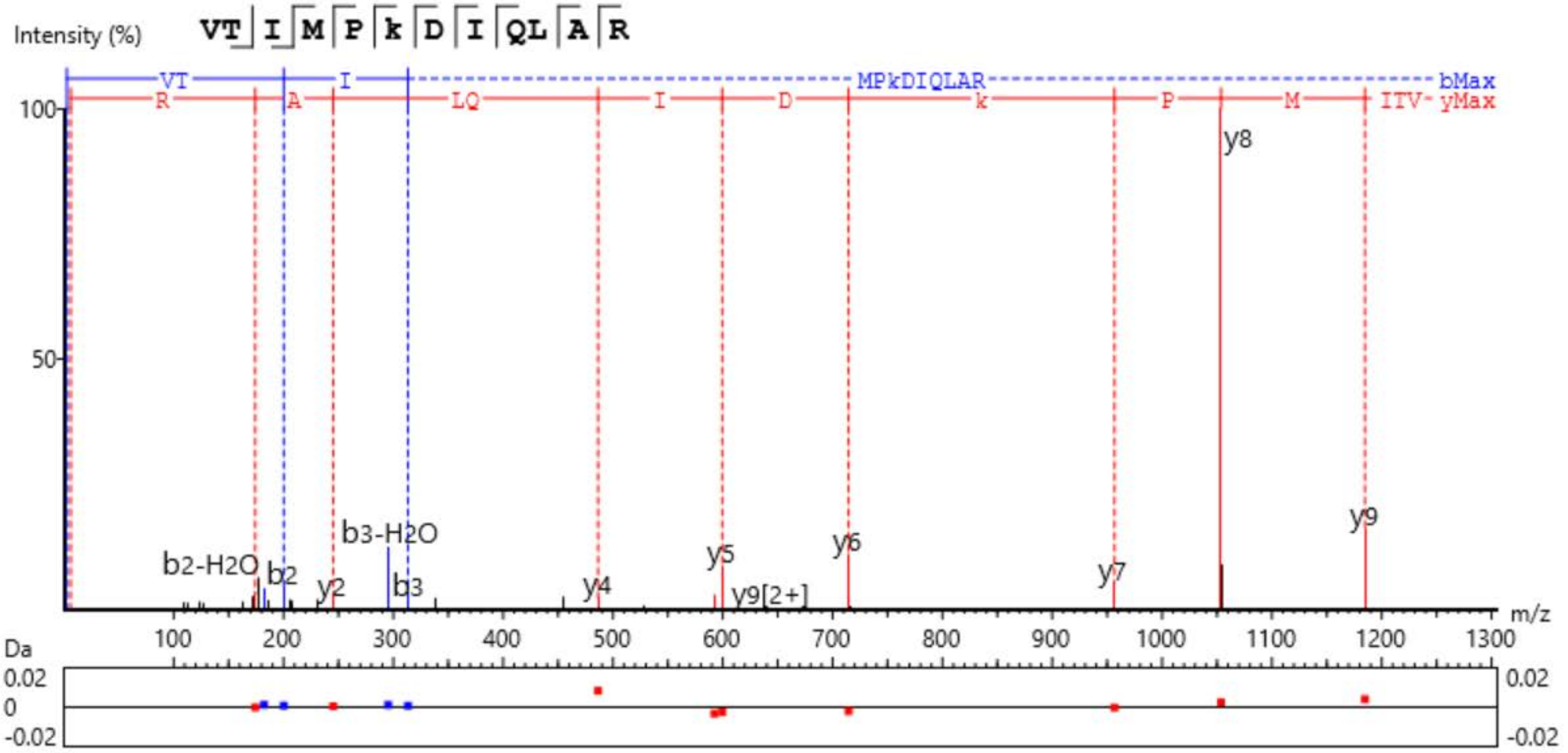

alignment   error map

Ion Match | Survey

#	b	b-H2O	b-NH3	b(2+)	Seq	y	y-H2O	y-NH3	y(2+)	#
1	100.08	82.07	83.05	50.54	V					12
2	201.12	183.11	184.10	101.06	T	1399.77	1381.76	1382.75	700.39	11
3	314.21	296.20	297.18	157.60	I	1298.72	1280.71	1281.70	649.86	10
4	445.25	427.24	428.22	223.12	M	1185.64	1167.63	1168.61	593.32	9
5	542.30	524.29	525.27	271.65	P	1054.60	1036.59	1037.57	527.80	8
6	784.44	766.43	767.41	392.72	K(+114.04)	957.55	939.54	940.52	479.27	7
7	899.47	881.46	882.44	450.23	D	715.41	697.40	698.38	358.20	6
8	1012.55	994.54	995.52	506.78	I	600.39	582.37	583.36	300.69	5
9	1140.61	1122.60	1123.58	570.80	Q	487.29	469.29	470.27	244.15	4
10	1253.69	1235.68	1236.67	627.35	L	359.24	341.23	342.21	180.12	3
11	1324.73	1306.72	1307.70	662.86	A	246.16	228.15	229.13	123.58	2
12					R	175.12	157.11	158.09	88.06	1

Sample 2 〉CL_010.raw 〉Feature Id: 24848, m/z: 682.8803, RT: 37.29, CV:-40.0, z: 2, -10lgP: 52.53

Scan 15023, m/z=682.8805, z=2, RT=37.26, CV=-40.0, Length=10, -10lgP=52.53, ppm=0.1 3/4

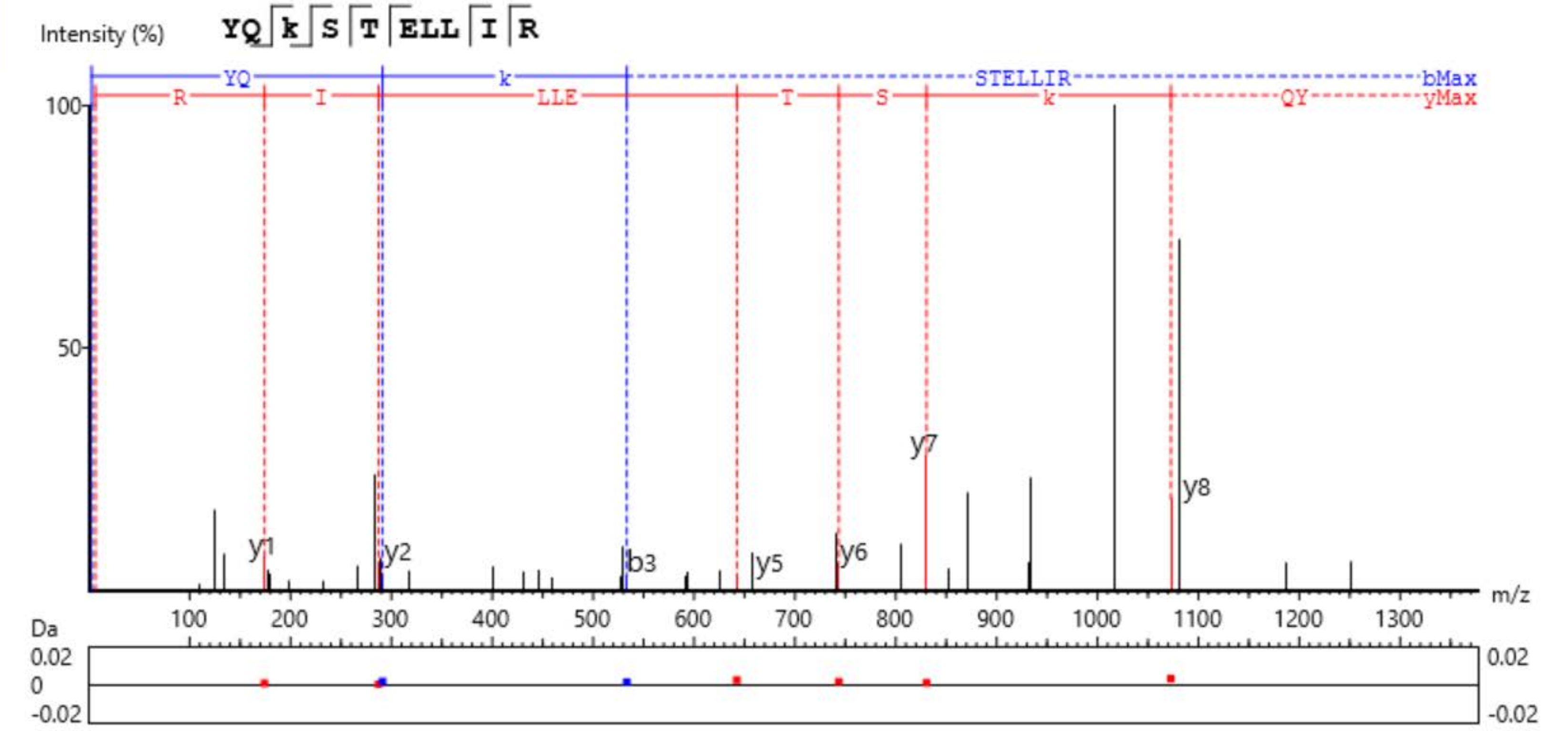

alignment error map

Ion Match | Survey

#	b	b-H2O	b-NH3	b(2+)	Seq	y	y-H2O	y-NH3	y(2+)	#
1	164.07	146.06	147.04	82.54	Y					10
2	292.13	274.12	275.10	146.56	Q	1201.69	1183.68	1184.66	601.34	9
3	534.27	516.26	517.24	267.63	K(+114.04)	1073.63	1055.62	1056.60	537.32	8
4	621.30	603.29	604.27	311.15	S	831.49	813.48	814.47	416.25	7
5	722.35	704.34	705.32	361.67	T	744.46	726.45	727.43	372.73	6
6	851.39	833.38	834.36	426.19	E	643.41	625.40	626.39	322.21	5
7	964.47	946.46	947.45	482.74	L	514.37	496.36	497.34	257.69	4
8	1077.56	1059.55	1060.53	539.28	L	401.29	383.28	384.26	201.14	3
9	1190.64	1172.63	1173.62	595.82	I	288.20	270.19	271.18	144.60	2
10					R	175.12	157.11	158.09	88.06	1

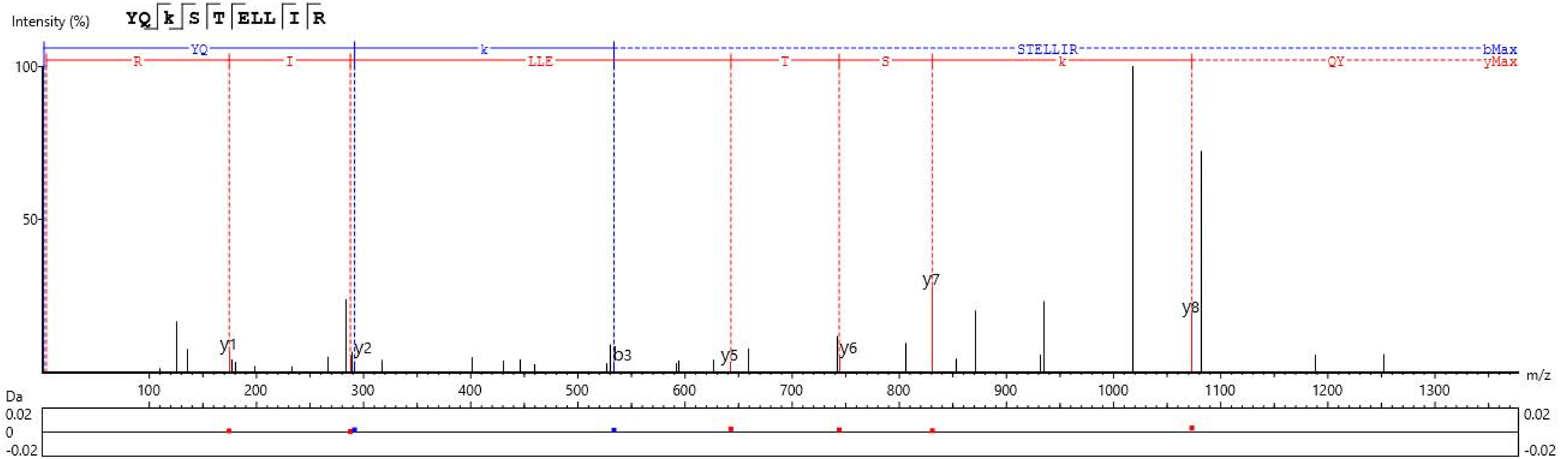

Intensity (%)
YQ k S T ELL I R
YQ
k
STELLIR
bMax
R
I
LLE
T
S
k
QY
yMax
100
50
y1
y2
b3
y5
y6
y7
y8
100
200
300
400
500
600
700
800
900
1000
1100
1200
1300
m/z
Da
0.02
0
-0.02
0.02
-0.02

Sample 2 | CL_010.raw | Feature Id: 21571, m/z: 571.8362, RT: 14.82, CV:-40.0, z: 2, -10lgP: 30.11

Scan 2501, m/z=571.8359, z=2, RT=15.01, CV=-40.0, Length=9, -10lgP=30.11, ppm=0.9 2/2

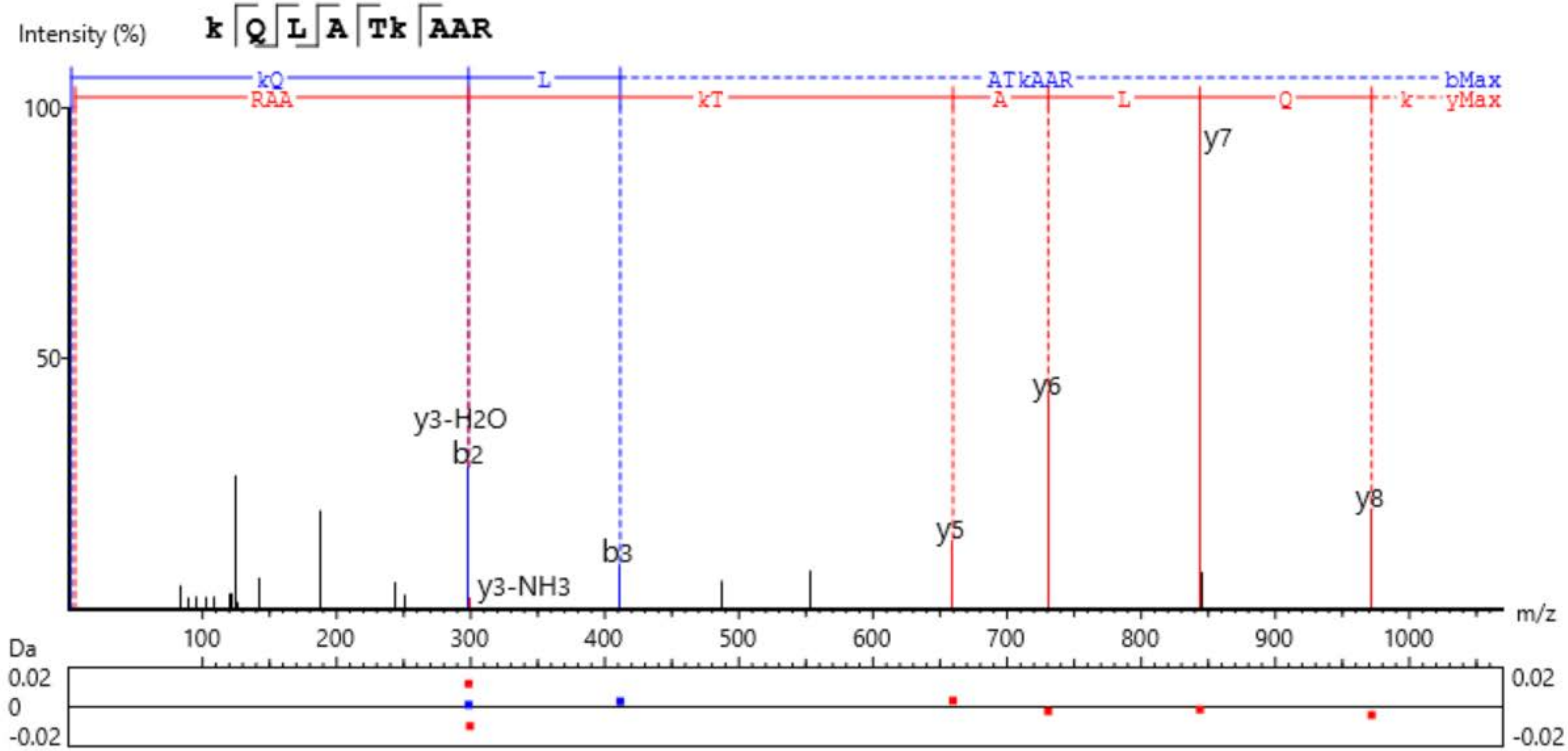

alignment error map

Ion Match Survey

#	b	b-H2O	b-NH3	b(2+)	Seq	y	y-H2O	y-NH3	y(2+)	#
1	171.11	153.10	154.09	86.06	K(+42.01)					9
2	299.17	281.16	282.14	150.09	Q	972.56	954.55	955.53	486.78	8
3	412.25	394.25	395.23	206.63	L	844.50	826.49	827.47	422.75	7
4	483.29	465.28	466.27	242.15	A	731.42	713.41	714.39	366.21	6
5	584.34	566.33	567.31	292.67	T	660.38	642.37	643.35	330.69	5
6	826.48	808.47	809.45	413.74	K(+114.04)	559.33	541.32	542.30	280.17	4
7	897.52	879.51	880.49	449.26	A	317.19	299.17	300.18	159.10	3
8	968.55	950.54	951.53	484.78	A	246.16	228.15	229.13	123.58	2
9					R	175.12	157.11	158.09	88.06	1

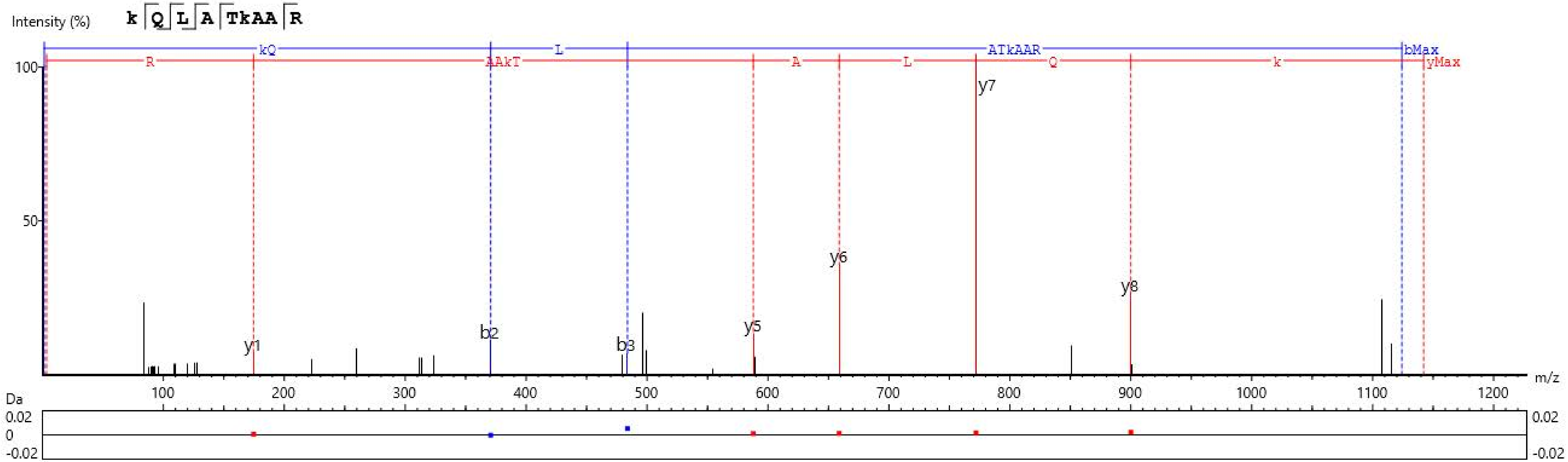

Intensity (%)
k Q L A TkAA R
kQ
L
ATkAAR
bMax
R
AAkT
A
L
Q
k
yMax
100
50
y7
y6
y8
y5
b2
b3
y1
m/z
100
200
300
400
500
600
700
800
900
1000
1100
1200
Da
0.02
0
-0.02

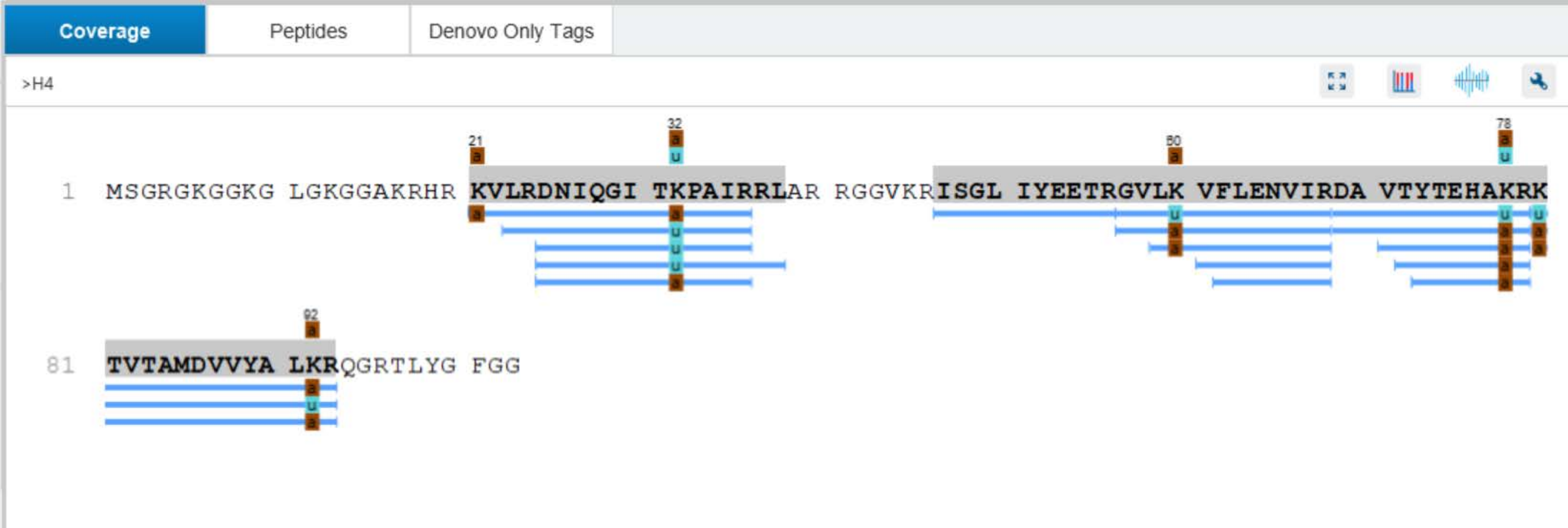
Coverage
Peptides
Denovo Only Tags
>H4
21
32
50
78
1 MSGRGKGGKG LGKGGAKRHR KVLRDNIQGI TKPAIRRLAR RGGVKRISGL IYEETRGVLK VFLENVIRDA VTYTEHAKRK
92
81 TVTAMDVVYA LKRQGRTLYG FGG

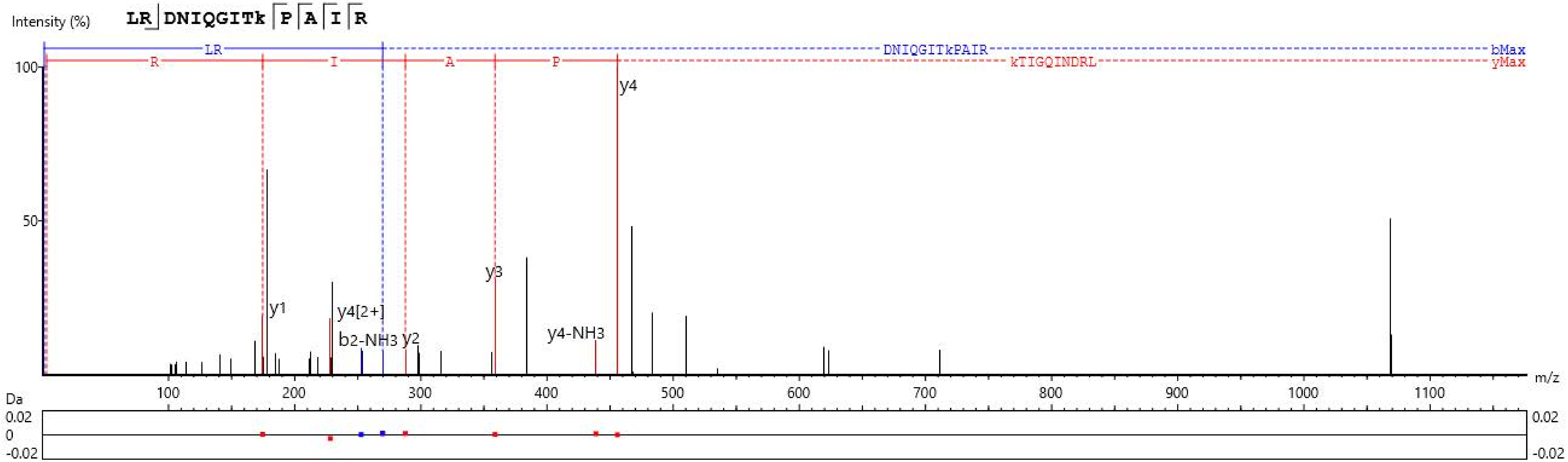

Intensity (%)
LR DNIQGITk P A I R
LR
DNIQGITkPAIR
bMax
R
I
A
P
kTIGQINDRL
yMax
y4
y3
y1
y4[2+]
b2-NH3
y2
y4-NH3
100
50
100
200
300
400
500
600
700
800
900
1000
1100
m/z
Da
0.02
0
-0.02

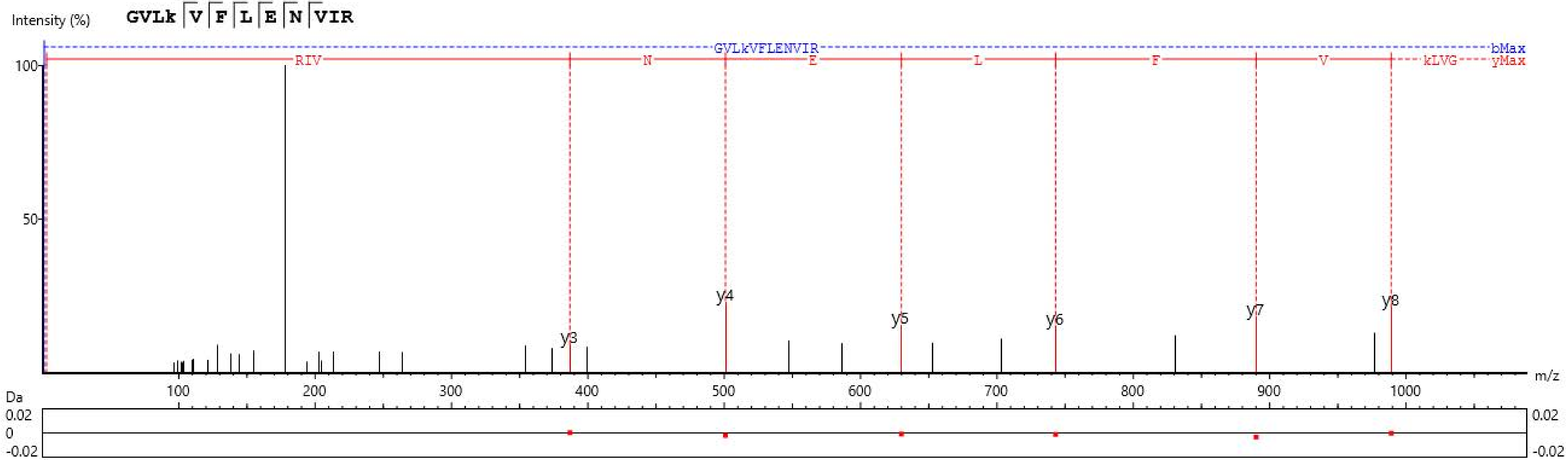

Intensity (%)
GVLk V F L E N VIR
GVLkVFLENVIR
bMax
RIV
N
E
L
F
V
kLVG
yMax
100
50
y3
y4
y5
y6
y7
y8
100
200
300
400
500
600
700
800
900
1000
m/z
Da
0.02
0
-0.02
0.02
-0.02

Sample 2 › CL_010.raw › Feature Id: 22025, m/z: 584.3227, RT: 69.49, CV:-40.0, z: 3, -10lgP: 57.94

Scan 36553, m/z=584.3224, z=3, RT=69.65, CV=-40.0, Length=14, -10lgP=57.94, ppm=0.9   2/3

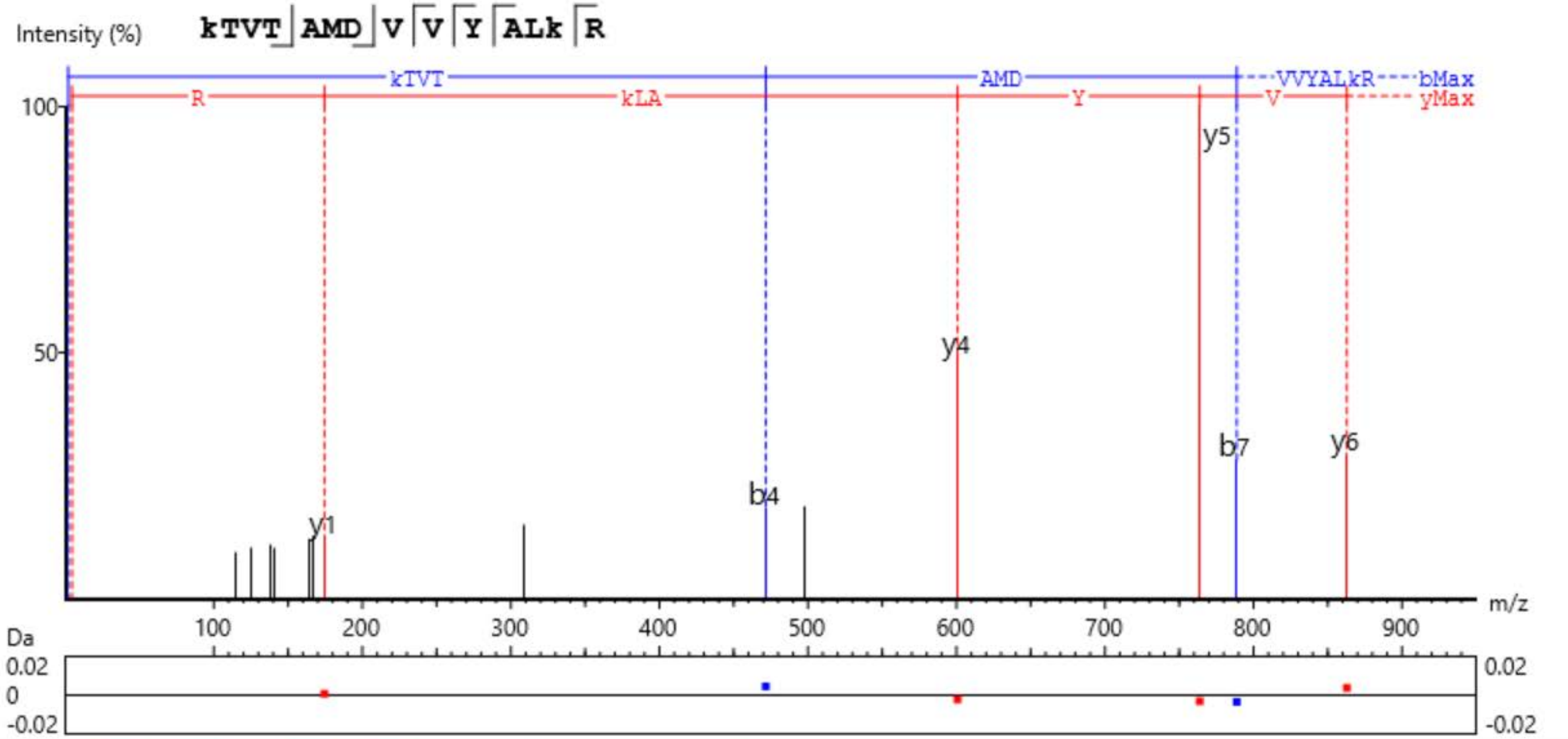

alignment   error map

Ion Match   Survey

#	b	b-H2O	b-NH3	b(2+)	Seq	y	y-H2O	y-NH3	y(2+)	#
1	171.11	153.10	154.09	86.06	K(+42.01)					14
2	272.16	254.15	255.13	136.58	T	1580.85	1562.84	1563.82	790.92	13
3	371.23	353.22	354.20	186.11	V	1479.80	1461.79	1462.77	740.40	12
4	472.27	454.27	455.25	236.64	T	1380.73	1362.72	1363.70	690.87	11
5	543.31	525.30	526.29	272.16	A	1279.68	1261.67	1262.66	640.34	10
6	674.35	656.34	657.33	337.68	M	1208.65	1190.64	1191.62	604.82	9
7	789.39	771.37	772.35	395.19	D	1077.61	1059.59	1060.58	539.30	8
8	888.45	870.44	871.42	444.73	V	962.58	944.57	945.55	481.79	7
9	987.52	969.51	970.49	494.26	V	863.51	845.50	846.48	432.25	6
10	1150.58	1132.57	1133.55	575.79	Y	764.44	746.43	747.41	382.72	5
11	1221.62	1203.61	1204.59	611.31	A	601.38	583.37	584.35	301.19	4
12	1334.70	1316.69	1317.68	667.85	L	530.34	512.33	513.31	265.67	3
13	1576.84	1558.83	1559.81	788.92	K(+114.04)	417.26	399.25	400.23	209.13	2
14					R	175.12	157.11	158.09	88.06	1

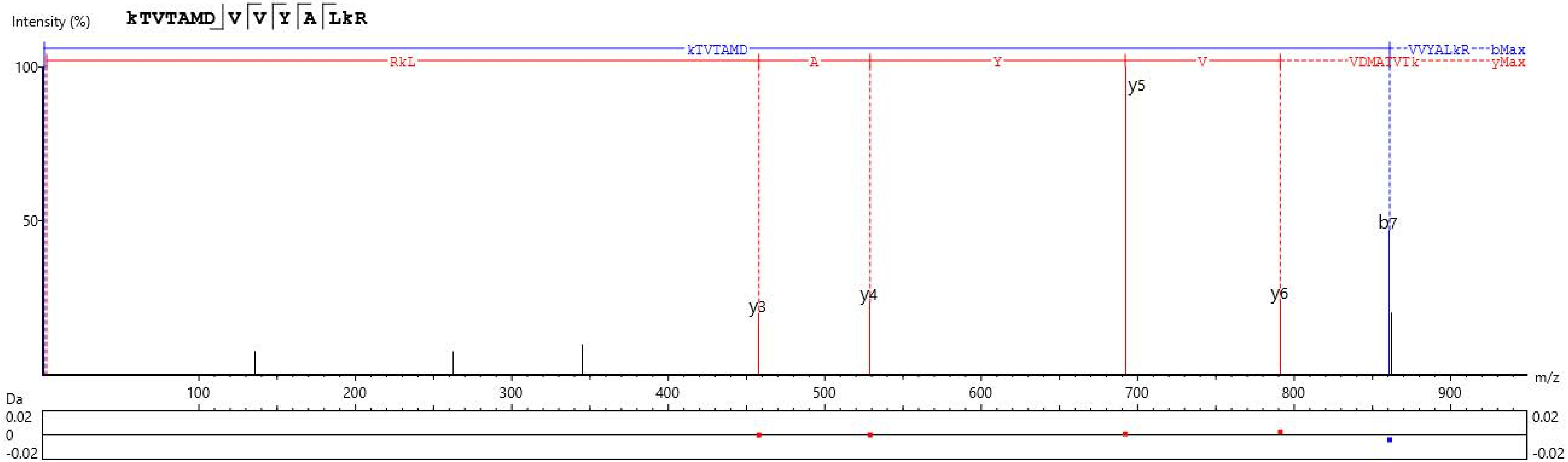

Intensity (%)
kTVTAMD V V Y A LkR
kTVTAMD
VVYALkR
bMax
RkL
A
Y
V
VDMATVTk
yMax
100
50
y5
b7
y3
y4
y6
m/z
100
200
300
400
500
600
700
800
900
Da
0.02
0
-0.02

Sample 2 | CL_010.raw | Feature Id: 6149, m/z: 500.9684, RT: 88.66, CV:-60.0, z: 3, -10lgP: 42.34

Scan 48664, m/z=500.9683, z=3, RT=88.60, CV=-60.0, Length=12, -10lgP=42.34, ppm=0.6   1/1

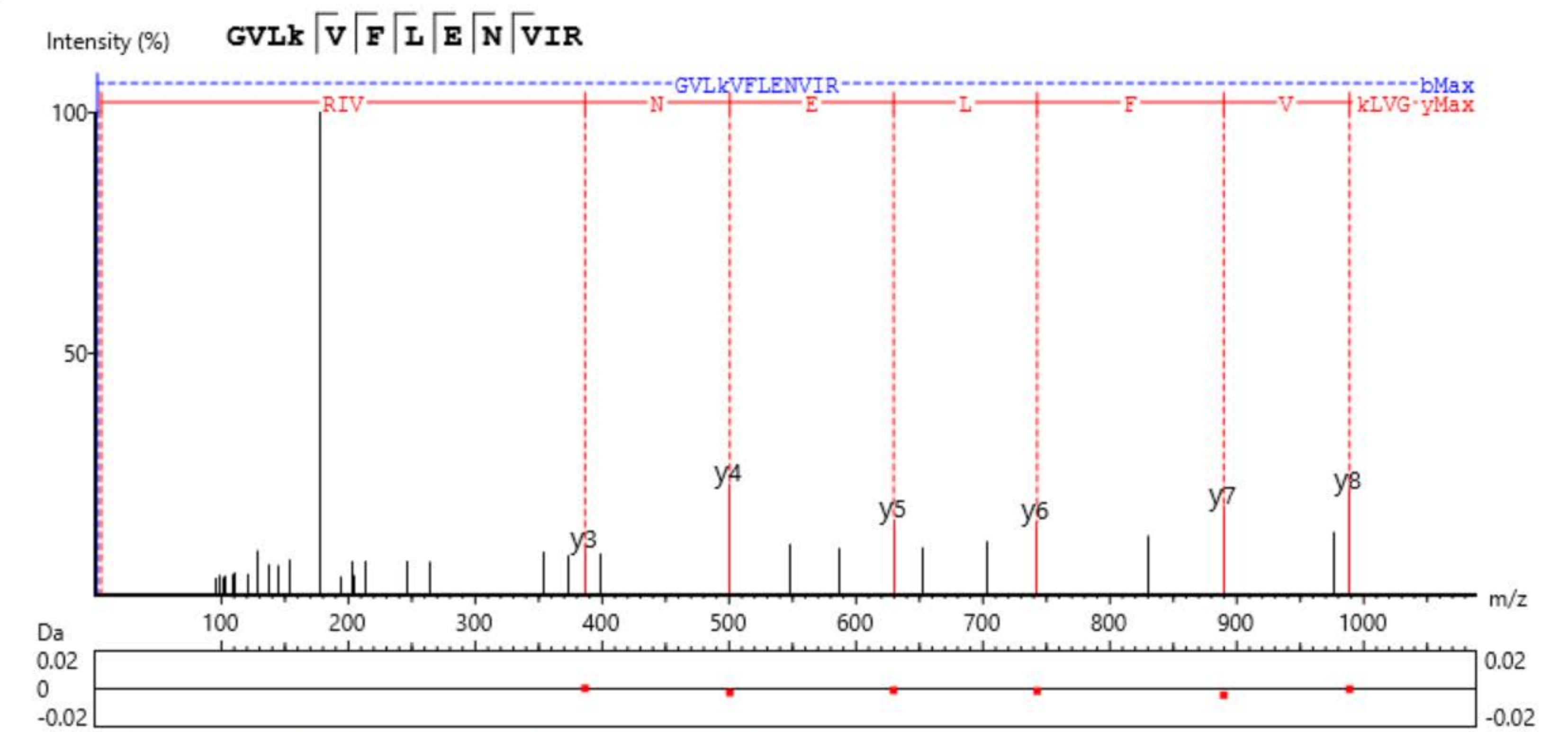

alignment   error map

Ion Match   Survey

#	b	b-H2O	b-NH3	b(2+)	Seq	y	y-H2O	y-NH3	y(2+)	#
1	58.03	40.02	41.00	29.51	G					12
2	157.10	139.09	140.07	79.05	V	1443.87	1425.86	1426.84	722.43	11
3	270.18	252.17	253.15	135.59	L	1344.80	1326.79	1327.77	672.90	10
4	512.32	494.31	495.29	256.66	K(+114.04)	1231.72	1213.71	1214.69	616.36	9
5	611.39	593.38	594.36	306.19	V	989.58	971.57	972.55	495.29	8
6	758.46	740.45	741.43	379.73	F	890.51	872.50	873.48	445.75	7
7	871.54	853.53	854.51	436.27	L	743.44	725.43	726.41	372.22	6
8	1000.58	982.57	983.56	500.79	E	630.36	612.35	613.33	315.68	5
9	1114.63	1096.62	1097.60	557.81	N	501.32	483.30	484.29	251.16	4
10	1213.69	1195.68	1196.67	607.35	V	387.27	369.26	370.24	194.14	3
11	1326.78	1308.77	1309.75	663.89	I	288.20	270.19	271.18	144.60	2
12					R	175.12	157.11	158.09	88.06	1

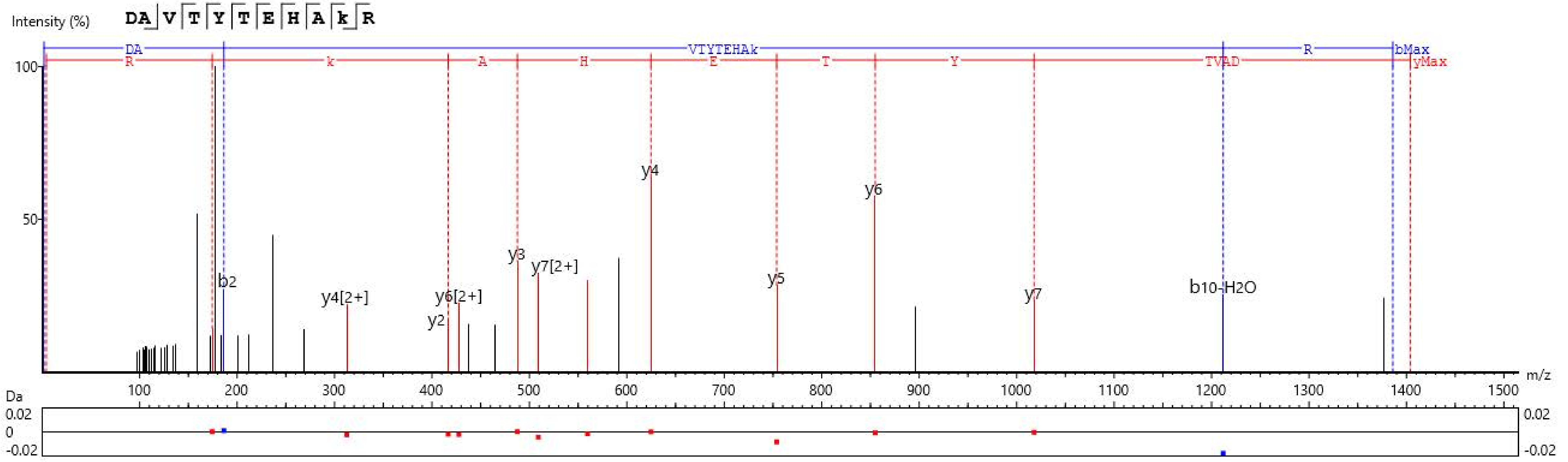

Intensity (%)
DA V T Y T E H A k R
DA
VTYTEHAk
R
bMax
R
k
A
H
E
T
Y
TVAD
yMax
100
50
b2
y4[2+]
y2
y6[2+]
y3
y7[2+]
y4
y5
y6
y7
b10-H2O
m/z
100
200
300
400
500
600
700
800
900
1000
1100
1200
1300
1400
1500
Da
0.02
0
-0.02

Sample 2 | CL_010.raw | Feature Id: 22024, m/z: 584.3226, RT: 72.83, CV:-40.0, z: 3, -10lgP: 61.31

Scan 38573, m/z=584.3224, z=3, RT=72.78, CV=-40.0, Length=14, -10lgP=61.31, ppm=0.7   1/2

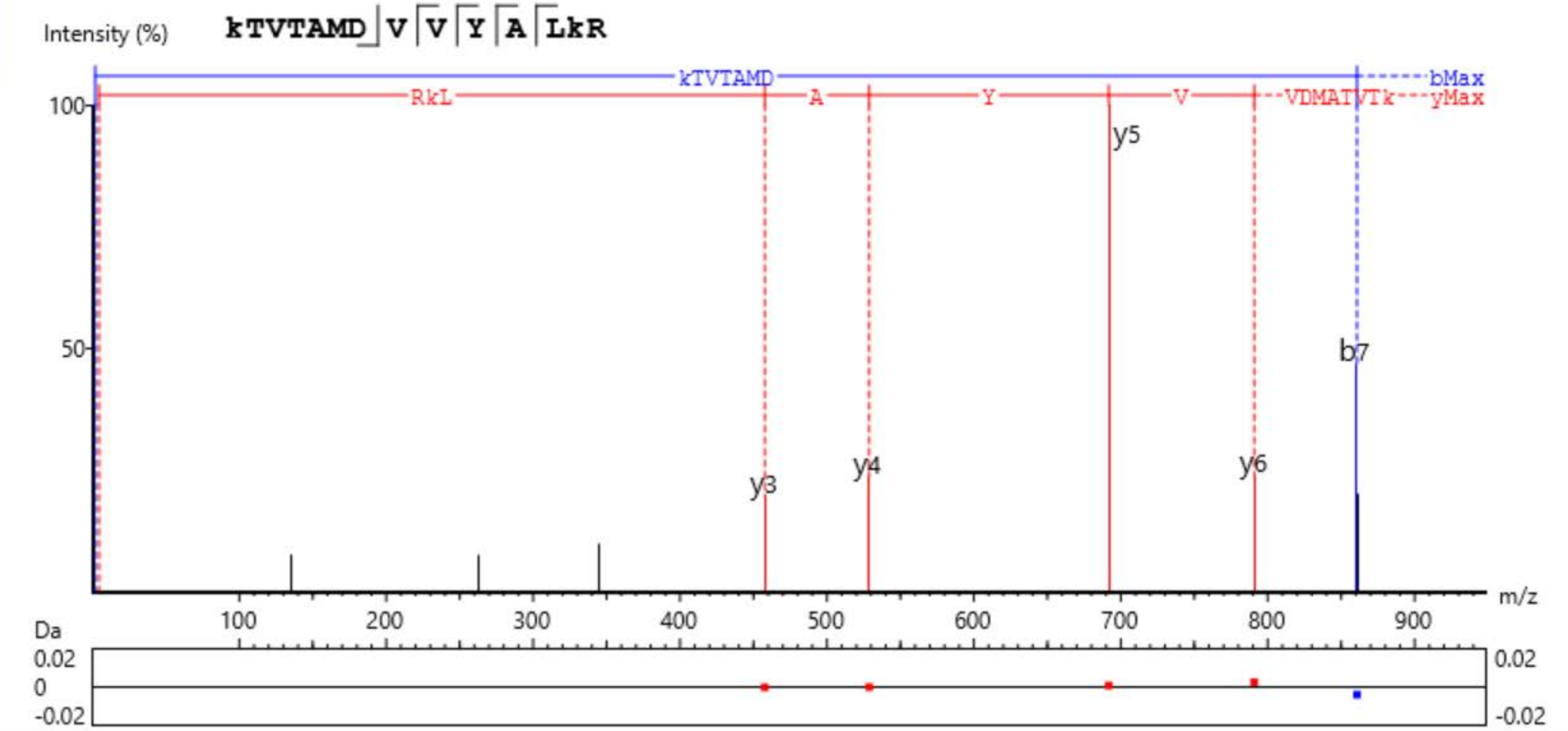

alignment   error map

Ion Match | Survey

#	b	b-H2O	b-NH3	b(2+)	Seq	y	y-H2O	y-NH3	y(2+)	#
1	243.15	225.14	226.12	122.07	K(+114.04)					14
2	344.19	326.18	327.17	172.60	T	1508.81	1490.80	1491.79	754.91	13
3	443.26	425.25	426.23	222.13	V	1407.77	1389.76	1390.74	704.38	12
4	544.31	526.30	527.28	272.65	T	1308.70	1290.69	1291.67	654.85	11
5	615.35	597.34	598.32	308.17	A	1207.65	1189.64	1190.62	604.33	10
6	746.39	728.38	729.36	373.69	M	1136.61	1118.60	1119.59	568.81	9
7	861.42	843.40	844.39	431.21	D	1005.57	987.56	988.55	503.29	8
8	960.48	942.47	943.46	480.74	V	890.55	872.54	873.52	445.77	7
9	1059.55	1041.54	1042.52	530.28	V	791.48	773.47	774.45	396.24	6
10	1222.61	1204.60	1205.59	611.81	Y	692.41	674.40	675.38	346.70	5
11	1293.65	1275.64	1276.62	647.33	A	529.35	511.34	512.32	265.17	4
12	1406.74	1388.72	1389.71	703.87	L	458.31	440.30	441.28	229.65	3
13	1576.84	1558.83	1559.81	788.92	K(+42.01)	345.22	327.21	328.20	173.11	2
14					R	175.12	157.11	158.09	88.06	1

Sample 11 › CL_09.raw › Feature Id: 4567, m/z: 468.9005, RT: 17.41, CV:-60.0, z: 3, -10lgP: 56.55

Scan 3398, m/z=468.9005, z=3, RT=17.51, CV=-60.0, Length=11, -10lgP=56.55, ppm=0.3   2/3

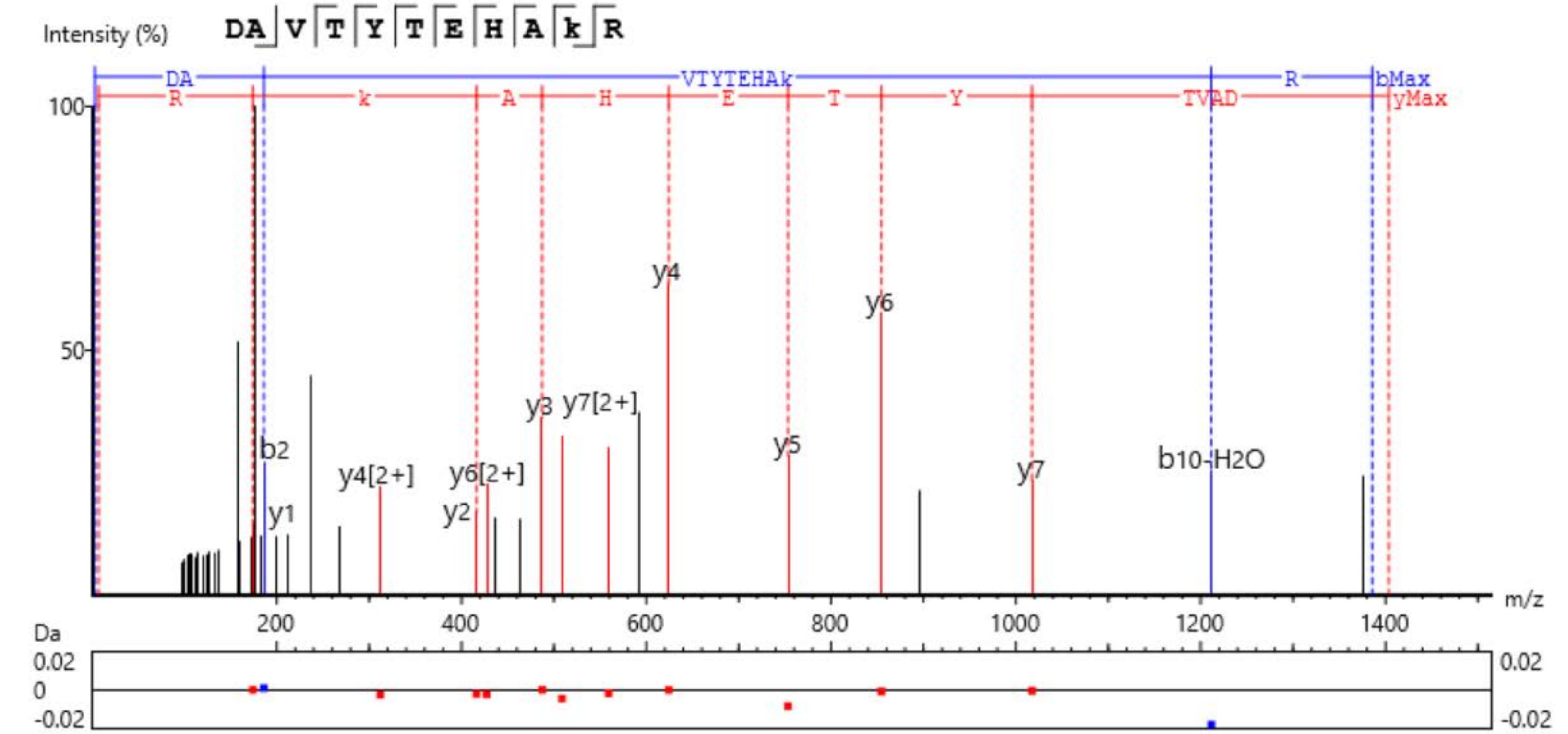

alignment   error map

Ion Match | Survey

#	b	b-H2O	b-NH3	b(2+)	Seq	y	y-H2O	y-NH3	y(2+)	#
1	116.03	98.02	99.01	58.52	D					11
2	187.07	169.06	170.04	94.04	A	1289.66	1271.65	1272.63	645.33	10
3	286.14	268.13	269.11	143.57	V	1218.62	1200.61	1201.60	609.81	9
4	387.19	369.18	370.16	194.09	T	1119.55	1101.54	1102.53	560.28	8
5	550.25	532.24	533.22	275.63	Y	1018.51	1000.50	1001.48	509.76	7
6	651.30	633.29	634.27	326.15	T	855.44	837.43	838.42	428.22	6
7	780.34	762.33	763.31	390.67	E	754.40	736.38	737.37	377.70	5
8	917.40	899.39	900.37	459.20	H	625.35	607.34	608.33	313.18	4
9	988.44	970.43	971.41	494.72	A	488.29	470.28	471.27	244.65	3
10	1230.58	1212.58	1213.55	615.79	K(+114.04)	417.26	399.25	400.23	209.13	2
11					R	175.12	157.11	158.09	88.06	1

Sample 2 > CL_010.raw > Feature Id: 26322, m/z: 720.4034, RT: 35.06, CV:-40.0, z: 2, -10lgP: 65.19

Scan 13628, m/z=720.4029, z=2, RT=35.11, CV=-40.0, Length=12, -10lgP=65.19, ppm=2.1   2/6

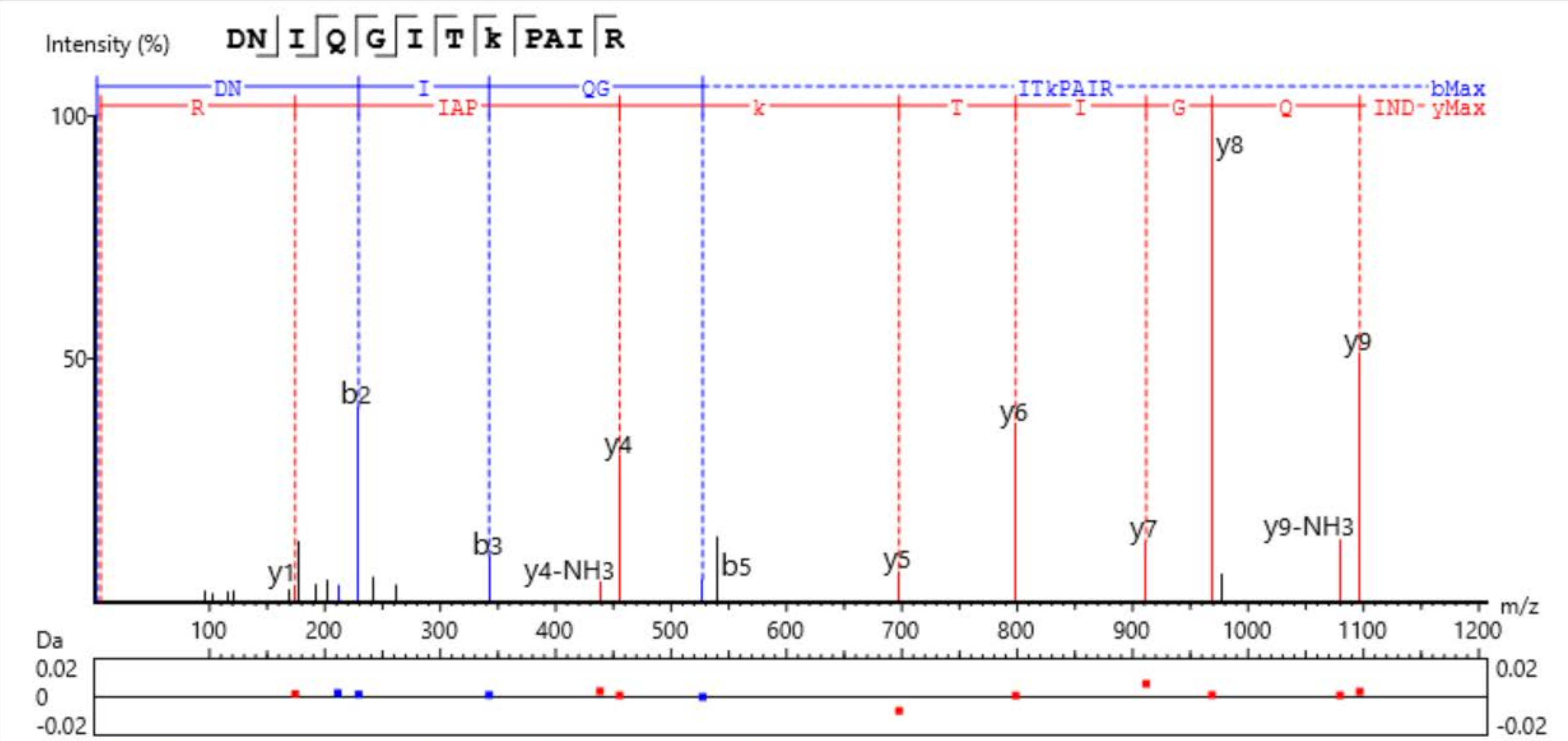

alignment   error map

Ion Match   Survey

#	b	b-H2O	b-NH3	b(2+)	Seq	y	y-H2O	y-NH3	y(2+)	#
1	116.03	98.02	99.01	58.52	D					12
2	230.08	212.07	213.05	115.54	N	1324.77	1306.76	1307.74	662.88	11
3	343.16	325.15	326.13	172.08	I	1210.73	1192.72	1193.70	605.86	10
4	471.22	453.21	454.19	236.11	Q	1097.64	1079.63	1080.62	549.32	9
5	528.24	510.23	511.21	264.62	G	969.58	951.57	952.56	485.29	8
6	641.33	623.32	624.30	321.16	I	912.56	894.55	895.54	456.78	7
7	742.37	724.36	725.35	371.69	T	799.48	781.47	782.45	400.24	6
8	984.51	966.50	967.48	492.76	K(+114.04)	698.44	680.42	681.40	349.72	5
9	1081.56	1063.55	1064.54	541.28	P	456.29	438.28	439.26	228.65	4
10	1152.60	1134.59	1135.57	576.80	A	359.24	341.23	342.21	180.12	3
11	1265.69	1247.67	1248.66	633.34	I	288.20	270.19	271.18	144.60	2
12					R	175.12	157.11	158.09	88.06	1

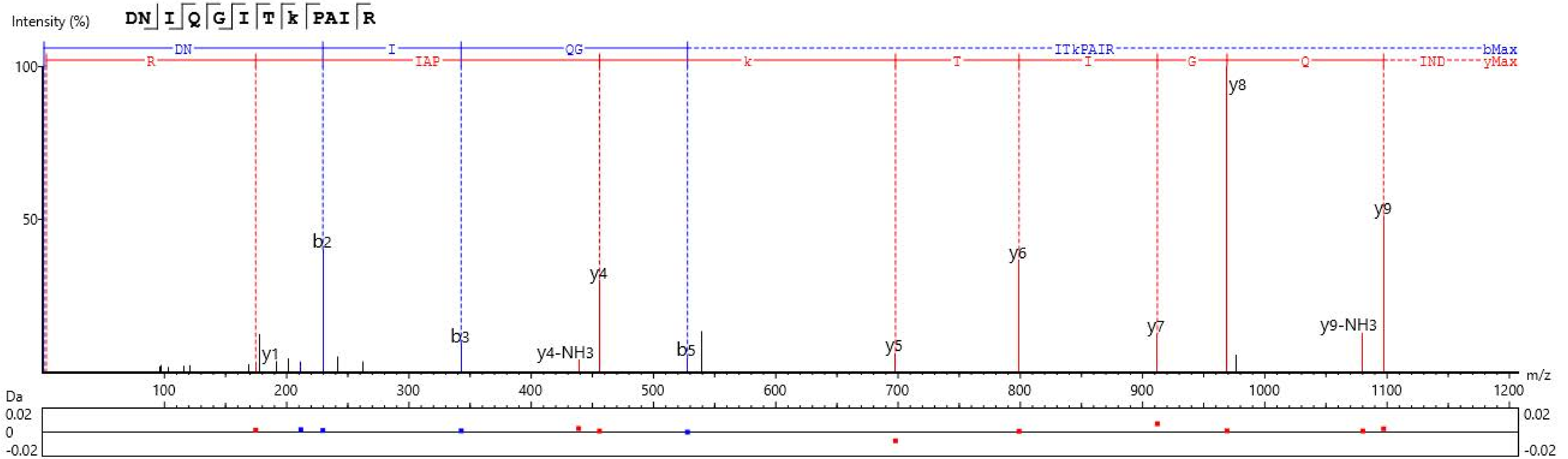

Intensity (%)
DN I Q G I T k PAI R
DN
I
QG
ITkPAIR
bMax
R
IAP
k
T
I
G
Q
IND
yMax
100
50
b2
b3
b5
y1
y4-NH3
y4
y5
y6
y7
y8
y9
y9-NH3
100
200
300
400
500
600
700
800
900
1000
1100
1200
m/z
Da
0.02
0
-0.02
0.02
-0.02

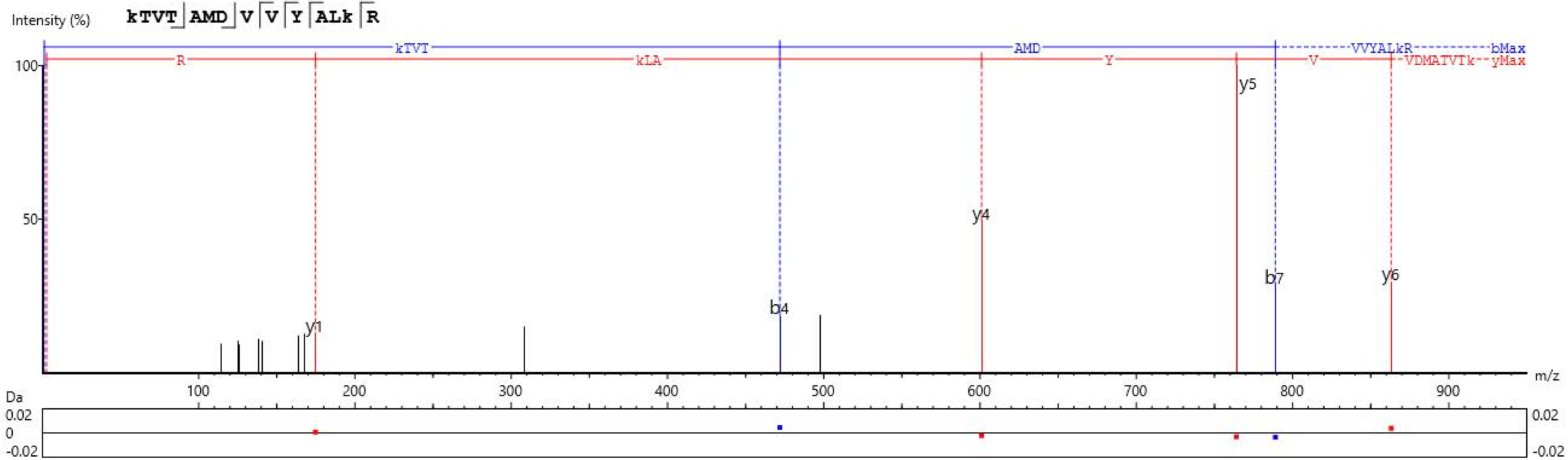
Intensity (%)
kTVT AMD V V Y ALk R
kTVT
AMD
VVYALkR
bMax
R
kLA
Y
V
VDMATVTk
yMax
100
50
y5
y4
b7
y6
b4
y1
m/z
100
200
300
400
500
600
700
800
900
Da
0.02
0
-0.02
0.02
-0.02

Sample 11 | CL_09.raw | Feature Id: 3221, m/z: 428.0008, RT: 37.43, CV:-60.0, z: 4, -10lgP: 18.78

Scan 14029, m/z=428.0000, z=4, RT=37.48, CV=-60.0, Length=14, -10lgP=18.78, ppm=-0.2   1/1

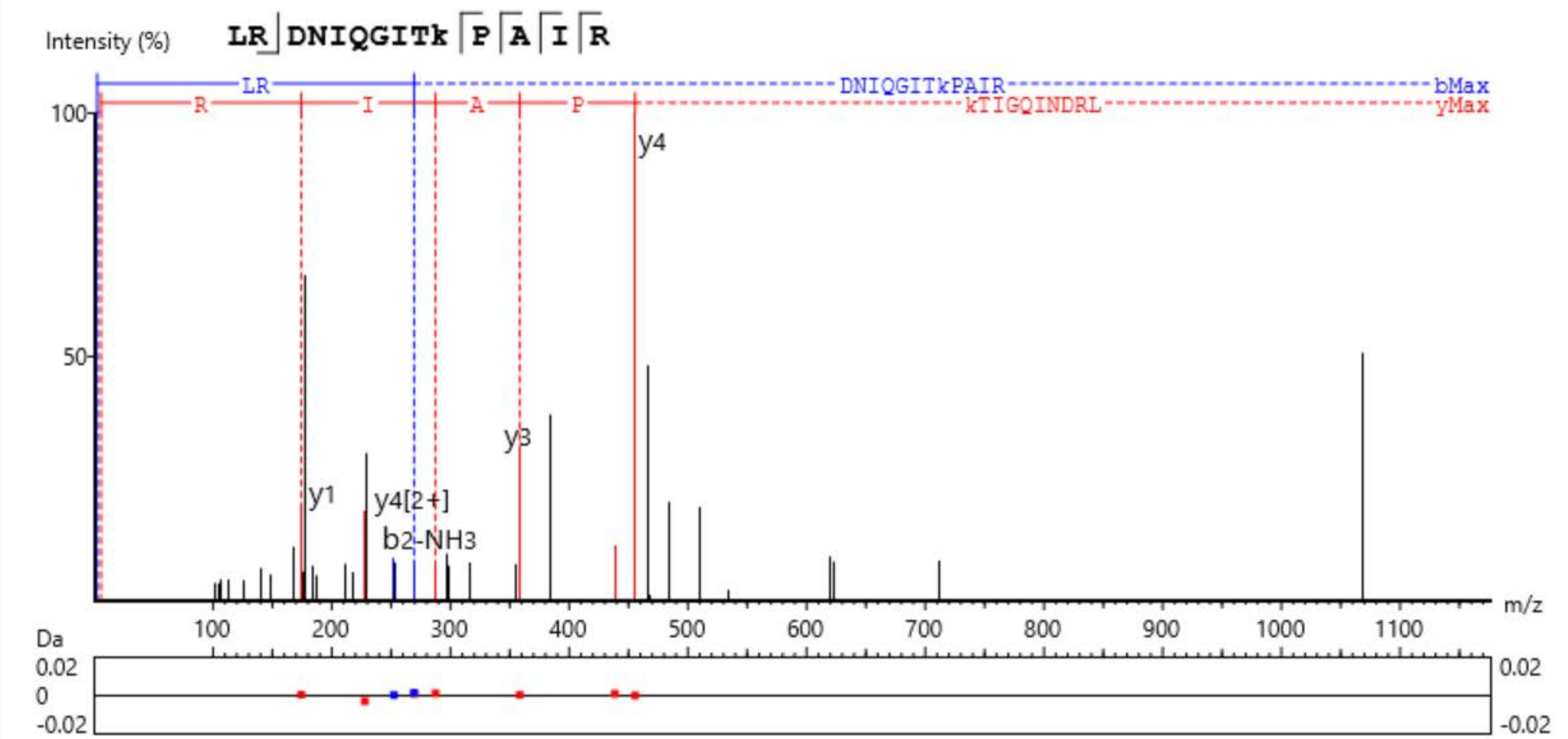

alignment   error map

Ion Match   Survey

#	b	b-H2O	b-NH3	b(2+)	Seq	y	y-H2O	y-NH3	y(2+)	#
1	114.09	96.08	97.06	57.55	L					14
2	270.19	252.18	253.17	135.60	R	1595.90	1577.89	1578.87	798.45	13
3	385.22	367.21	368.19	193.11	D	1439.80	1421.79	1422.77	720.40	12
4	499.26	481.25	482.24	250.13	N	1324.77	1306.76	1307.74	662.88	11
5	612.35	594.34	595.32	306.67	I	1210.73	1192.72	1193.70	605.86	10
6	740.41	722.39	723.38	370.70	Q	1097.64	1079.63	1080.62	549.32	9
7	797.43	779.42	780.40	399.21	G	969.58	951.57	952.56	485.29	8
8	910.51	892.50	893.48	455.76	I	912.56	894.55	895.54	456.78	7
9	1011.56	993.55	994.53	506.28	T	799.48	781.47	782.45	400.24	6
10	1253.70	1235.69	1236.67	627.35	K(+114.04)	698.43	680.42	681.40	349.72	5
11	1350.75	1332.74	1333.72	675.87	P	456.29	438.28	439.27	228.65	4
12	1421.79	1403.78	1404.76	711.39	A	359.24	341.23	342.21	180.12	3
13	1534.87	1516.86	1517.84	767.94	I	288.20	270.19	271.18	144.60	2
14					R	175.12	157.11	158.09	88.06	1

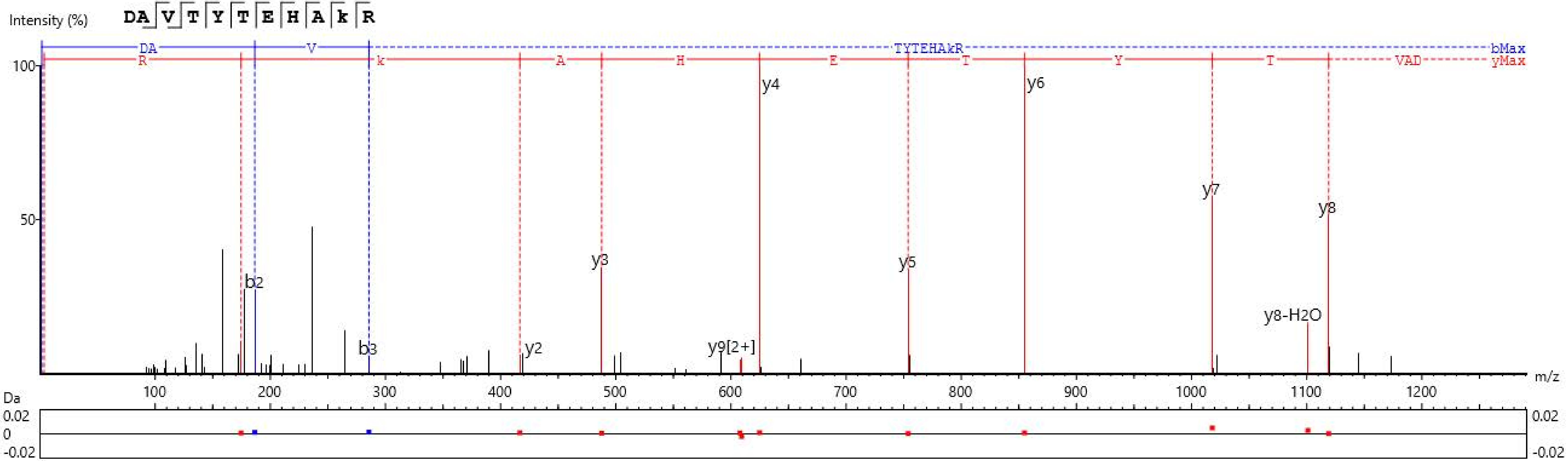

Intensity (%)
DAVTYTEHAkR
DA
V
TYTEHAkR
bMax
R
k
A
H
E
T
Y
T
VAD
yMax
100
50
b2
b3
y2
y3
y4
y5
y6
y7
y8
y9[2+]
y8-H2O
m/z
100
200
300
400
500
600
700
800
900
1000
1100
1200
Da
0.02
0
-0.02

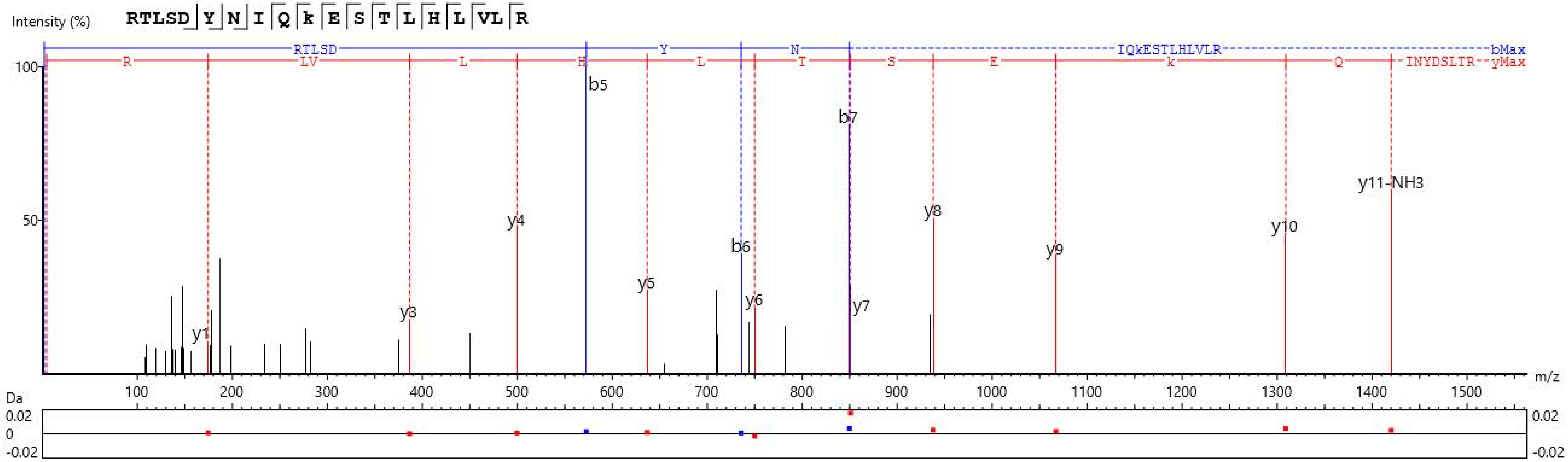

Intensity (%)
RTLSD Y N I Q k E S T L H L VL R
RTLSD
Y
N
IQkESTLHLVLR
bMax
R
LV
L
H
L
T
S
E
k
Q
INYDSLTR
yMax
b5
b7
y11-NH3
y4
y8
y10
b6
y9
y5
y6
y7
y3
y1
100
50
m/z
100
200
300
400
500
600
700
800
900
1000
1100
1200
1300
1400
1500
Da
0.02
0
-0.02

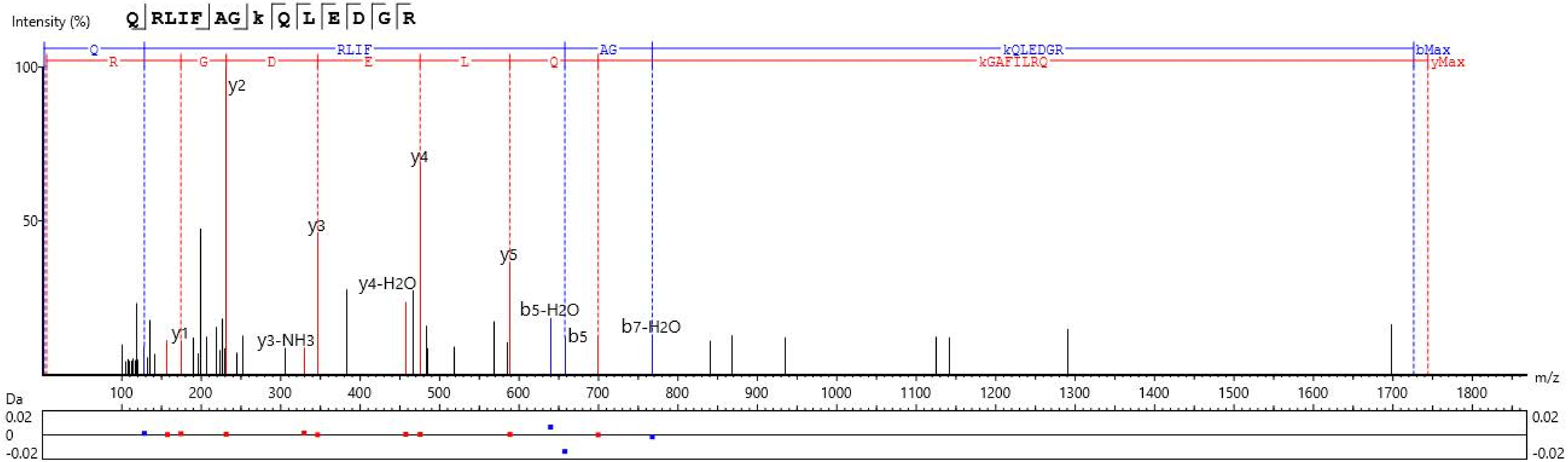

Intensity (%)
Q RLIF AG k Q L E D G R
Q
RLIF
AG
kQLEDGR
bMax
R
G
D
E
L
Q
kGAFILRQ
yMax
100
50
y2
y4
y3
y5
y4-H2O
b5-H2O
b5
b7-H2O
y1
y3-NH3
m/z
100
200
300
400
500
600
700
800
900
1000
1100
1200
1300
1400
1500
1600
1700
1800
Da
0.02
0
-0.02

Sample 11 | CL_09.raw | Feature Id: 742, m/z: 539.6334, RT: 77.31, CV:-80.0, z: 3, -10lgP: 43.38

Scan 37501, m/z=539.6346, z=3, RT=77.46, CV=-80.0, Length=13, -10lgP=43.38, ppm=-0.7   3/4

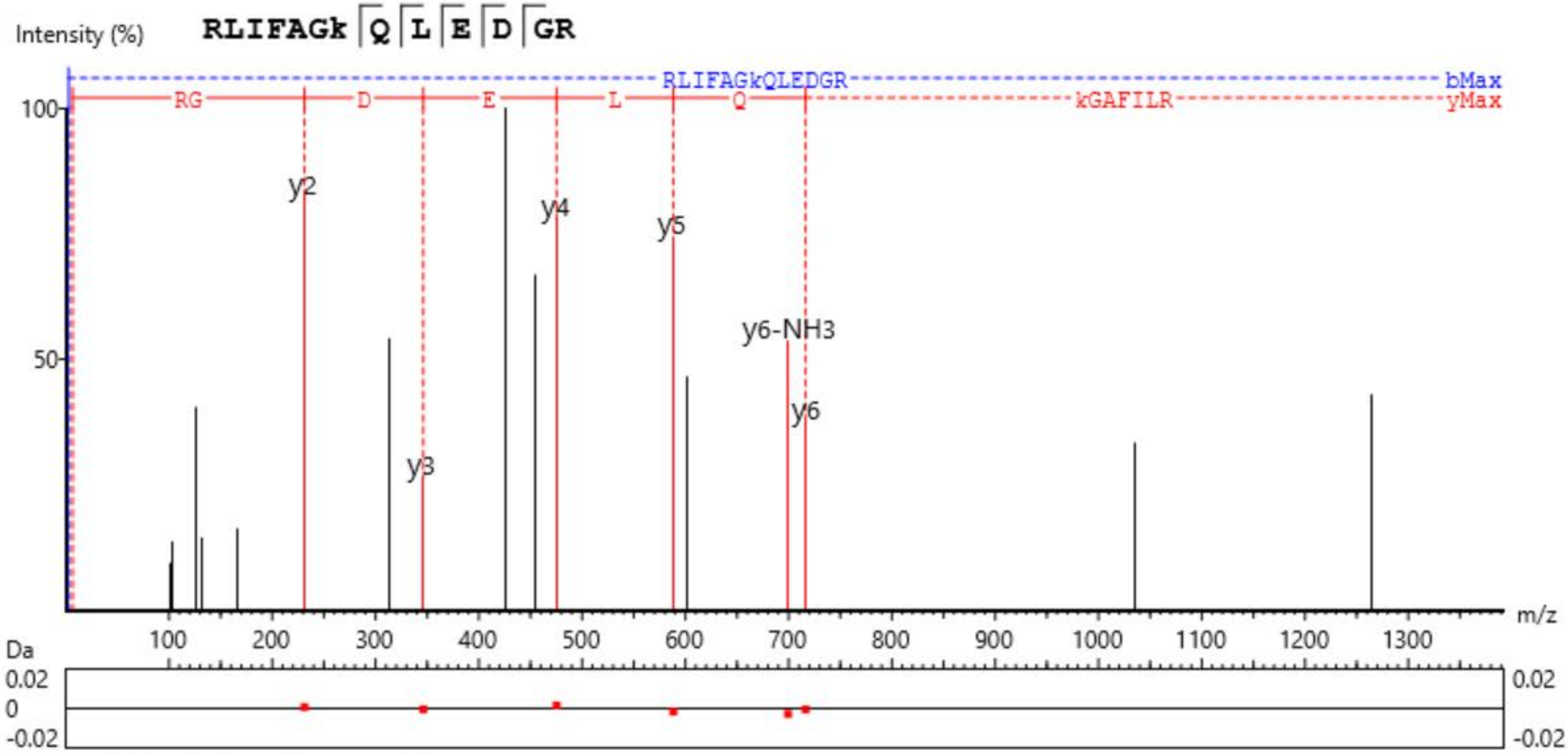

alignment   error map

Ion Match | Survey

#	b	b-H2O	b-NH3	b(2+)	Seq	y	y-H2O	y-NH3	y(2+)	#
1	157.11	139.10	140.08	79.05	R					13
2	270.19	252.18	253.17	135.60	L	1460.79	1442.78	1443.76	730.89	12
3	383.28	365.27	366.25	192.14	I	1347.70	1329.69	1330.67	674.35	11
4	530.35	512.33	513.32	265.67	F	1234.62	1216.61	1217.59	617.81	10
5	601.38	583.37	584.36	301.19	A	1087.55	1069.54	1070.52	544.27	9
6	658.40	640.39	641.38	329.70	G	1016.51	998.50	999.48	508.76	8
7	900.54	882.53	883.51	450.77	K(+114.04)	959.49	941.48	942.46	480.25	7
8	1028.60	1010.59	1011.57	514.80	Q	717.35	699.34	700.33	359.18	6
9	1141.68	1123.67	1124.66	571.34	L	589.30	571.28	572.27	295.15	5
10	1270.73	1252.72	1253.70	635.86	E	476.21	458.20	459.18	238.60	4
11	1385.75	1367.74	1368.73	693.38	D	347.17	329.16	330.14	174.08	3
12	1442.78	1424.77	1425.75	721.89	G	232.14	214.13	215.11	116.57	2
13					R	175.12	157.11	158.09	88.06	1

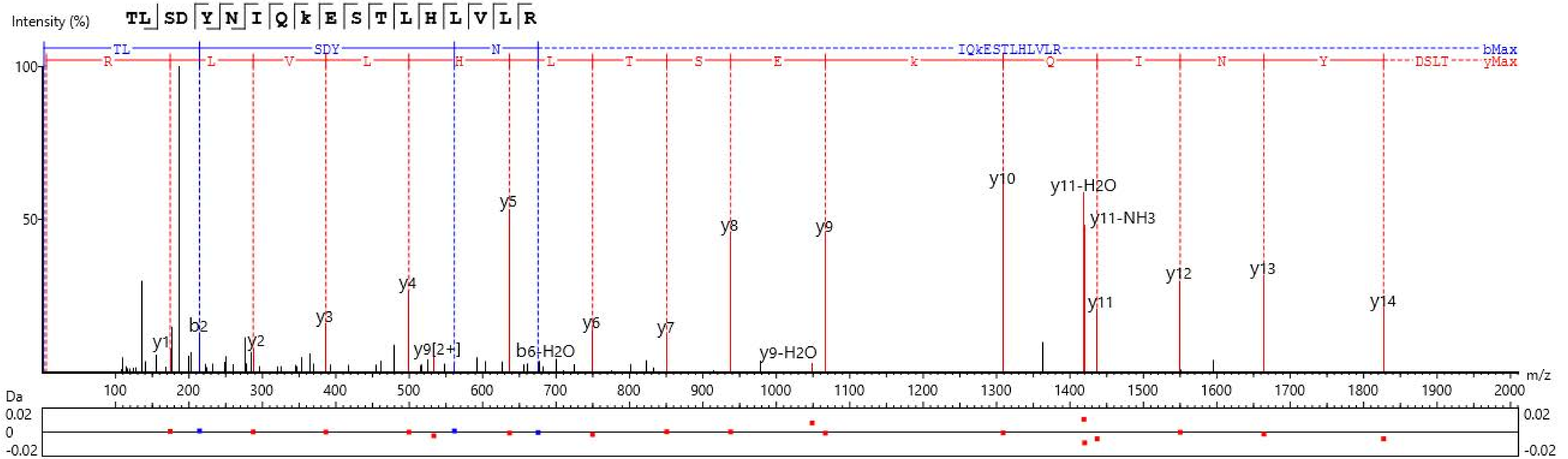
Intensity (%)
TL SD Y N I Q k E S T L H L V L R
TL SDY N IQkESTLHLVLR bMax
R L V L H L T S E k Q I N Y DSLT yMax
y1 b2 y2 y3 y4 y9[2+] y5 b6-H2O y6 y7 y8 y9-H2O y9 y10 y11-H2O y11-NH3 y11 y12 y13 y14
m/z
Da
0.02
0
-0.02

Sample 11 | CL_09.raw | Feature Id: 22362, m/z: 598.3025, RT: 30.73, CV:-40.0, z: 2, -10lgP: 39.47

Scan 9859, m/z=598.3020, z=2, RT=30.80, CV=-40.0, Length=9, -10lgP=39.47, ppm=2.0 2/2

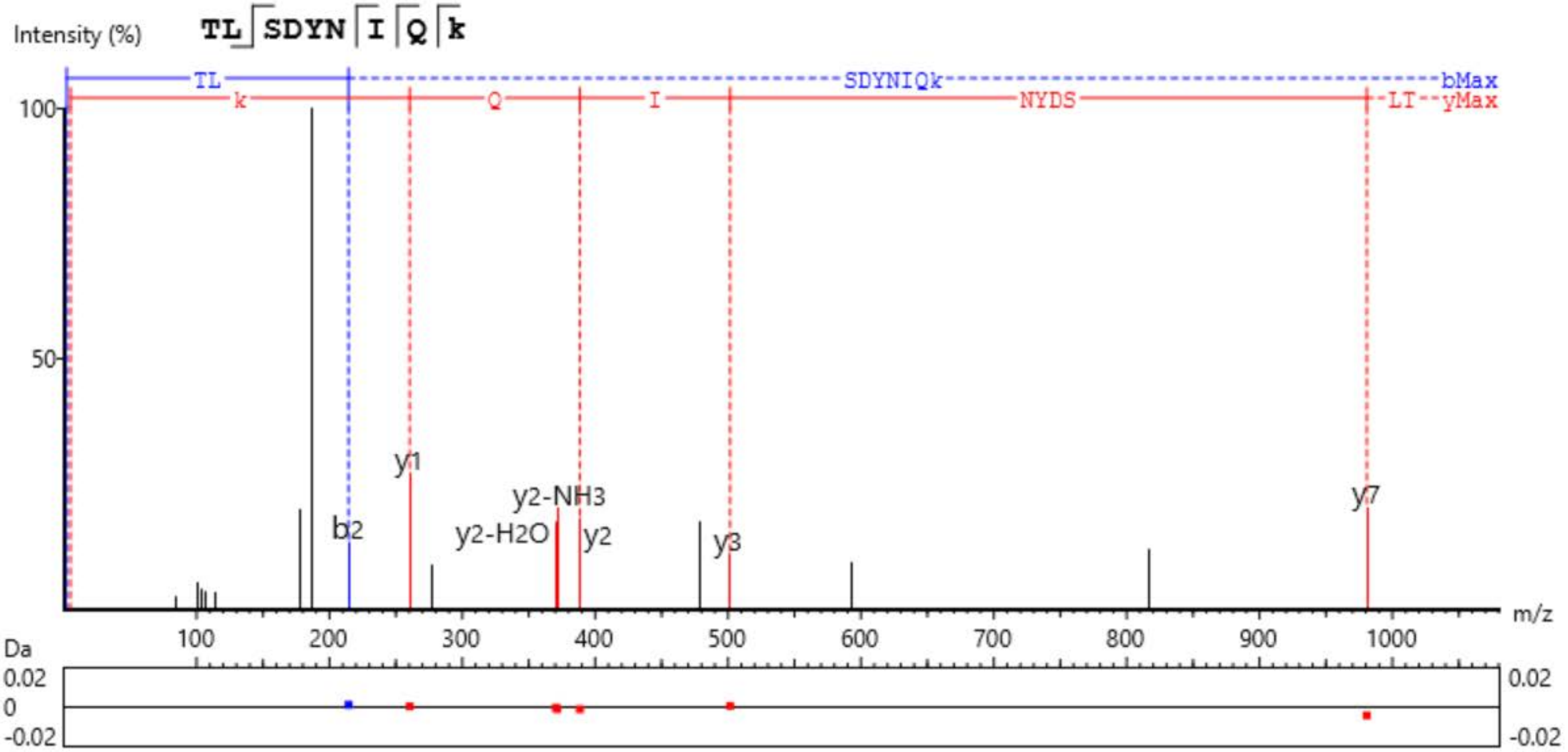

alignment error map

Ion Match | Survey

#	b	b-H2O	b-NH3	b(2+)	Seq	y	y-H2O	y-NH3	y(2+)	#
1	102.06	84.04	85.03	51.53	T					9
2	215.14	197.13	198.11	108.07	L	1094.55	1076.54	1077.52	547.77	8
3	302.17	284.16	285.14	151.59	S	981.47	963.45	964.44	491.23	7
4	417.20	399.19	400.17	209.10	D	894.43	876.42	877.40	447.72	6
5	580.26	562.25	563.23	290.63	Y	779.40	761.39	762.38	390.20	5
6	694.30	676.29	677.28	347.65	N	616.34	598.33	599.31	308.67	4
7	807.39	789.38	790.36	404.19	I	502.30	484.29	485.27	251.65	3
8	935.45	917.44	918.42	468.22	Q	389.22	371.20	372.19	195.11	2
9					K(+114.04)	261.16	243.15	244.13	131.08	1

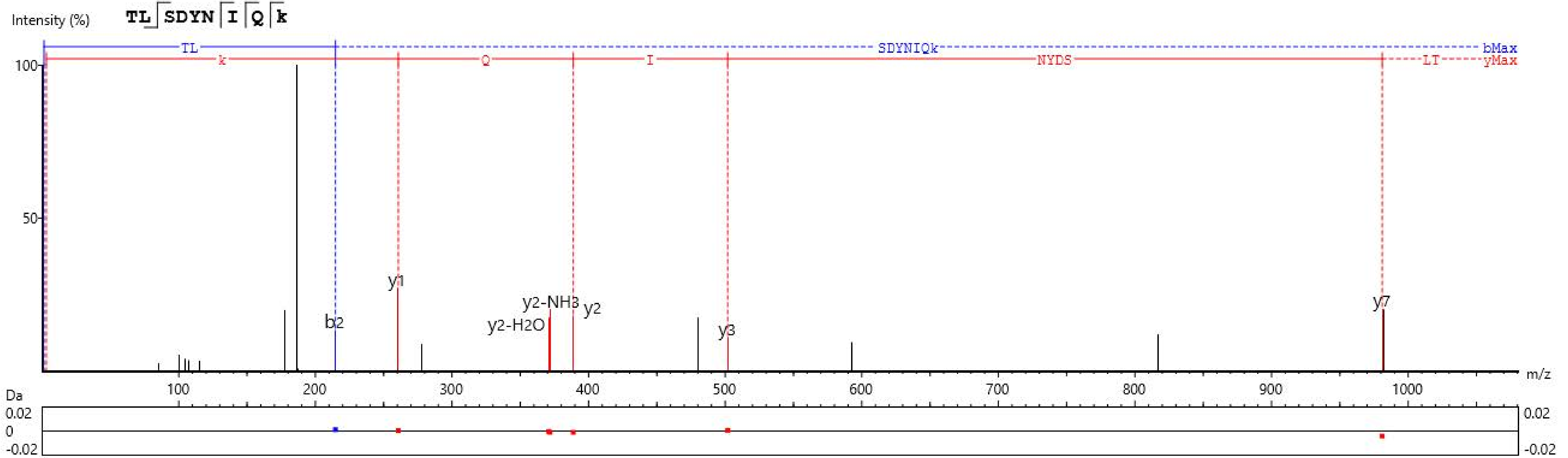
Intensity (%)
TL SDYN I Q k
TL
SDYNIQk
bMax
k
Q
I
NYDS
LT
yMax
100
50
y1
b2
y2-NH3
y2-H2O
y2
y3
y7
m/z
100
200
300
400
500
600
700
800
900
1000
Da
0.02
0
-0.02
0.02
-0.02

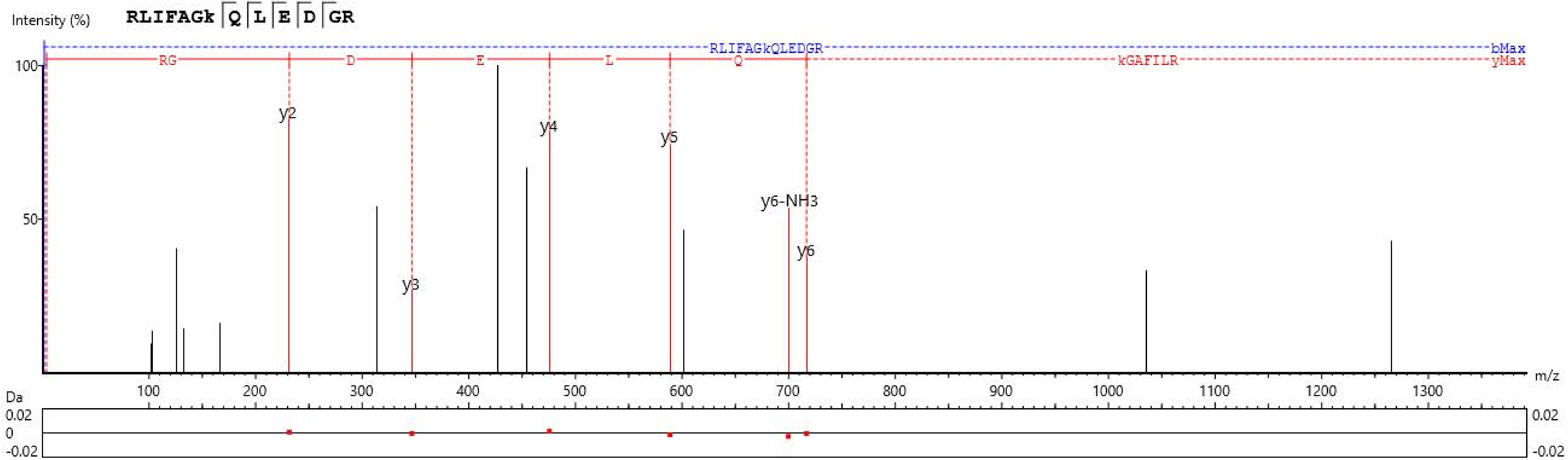

Intensity (%)
RLIFAGk Q L E D GR
RLIFAGkQLEDGR
bMax
RG
D
E
L
Q
kGAFILR
yMax
100
50
y2
y3
y4
y5
y6-NH3
y6
m/z
100
200
300
400
500
600
700
800
900
1000
1100
1200
1300
Da
0.02
0
-0.02
0.02
-0.02

Sample 2 | CL_010.raw | Feature Id: 0, m/z: 436.9938, RT: 43.17, CV:-60.0, z: 4, -10lgP: 44.06

Scan 18947, m/z=436.7429, z=4, RT=43.17, CV=-60.0, Length=14, -10lgP=44.06, ppm=4.7   1/1

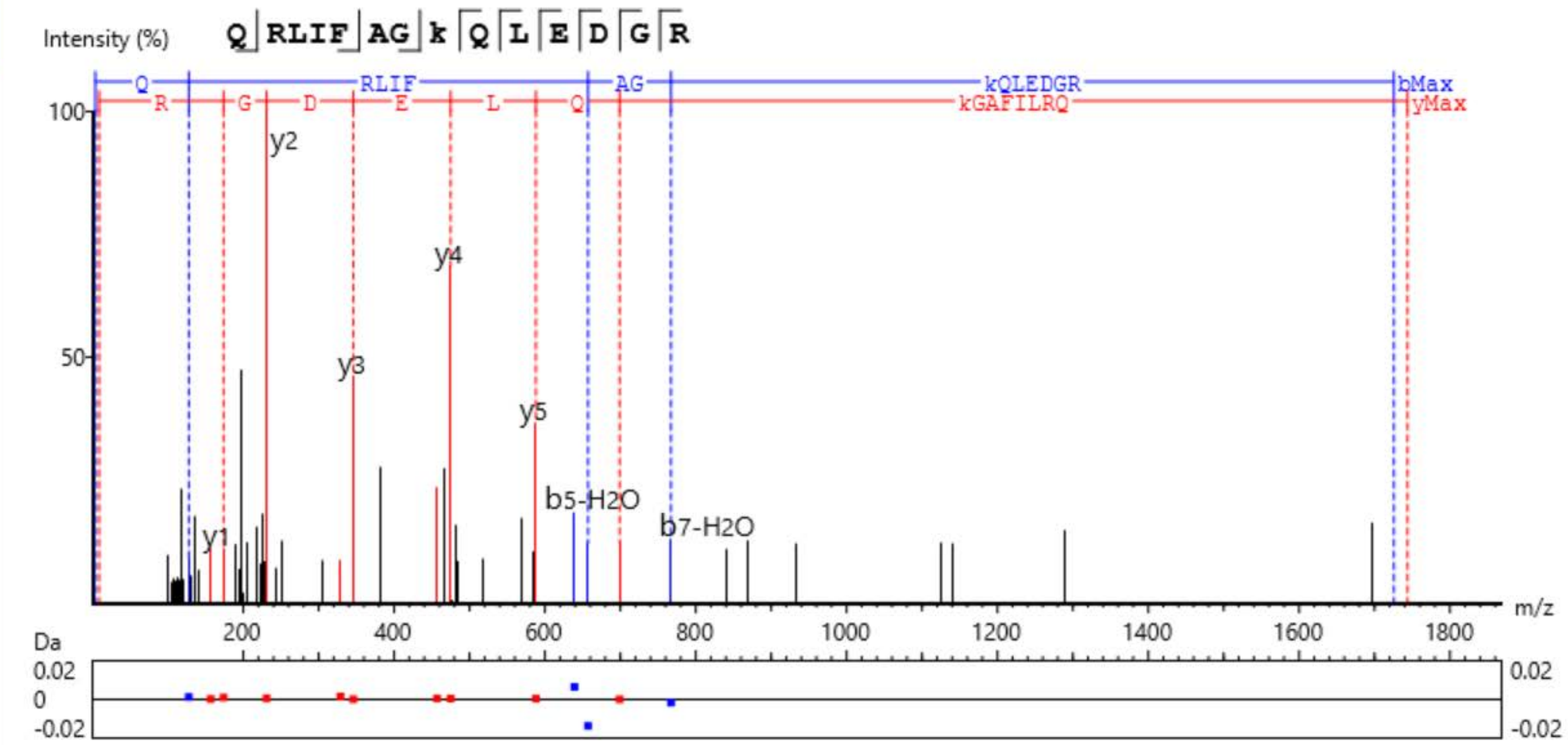

alignment   error map

Ion Match | Survey

#	b	b-H2O	b-NH3	b(2+)	Seq	y	y-H2O	y-NH3	y(2+)	#
1	129.07	111.06	112.04	65.03	Q					14
2	285.17	267.16	268.14	143.08	R	1616.89	1598.88	1599.86	808.94	13
3	398.25	380.24	381.22	199.63	L	1460.79	1442.78	1443.76	730.89	12
4	511.34	493.33	494.31	256.17	I	1347.70	1329.69	1330.67	674.35	11
5	658.42	640.39	641.38	329.70	F	1234.62	1216.61	1217.59	617.81	10
6	729.44	711.43	712.41	365.22	A	1087.55	1069.54	1070.52	544.27	9
7	786.46	768.45	769.44	393.73	G	1016.51	998.50	999.48	508.76	8
8	1028.60	1010.59	1011.57	514.80	K(+114.04)	959.49	941.48	942.46	480.25	7
9	1156.66	1138.65	1139.63	578.83	Q	717.35	699.34	700.33	359.18	6
10	1269.74	1251.73	1252.72	635.37	L	589.29	571.28	572.27	295.15	5
11	1398.79	1380.78	1381.76	699.89	E	476.21	458.20	459.18	238.60	4
12	1513.81	1495.80	1496.79	757.41	D	347.17	329.16	330.14	174.08	3
13	1570.83	1552.82	1553.81	785.92	G	232.14	214.13	215.11	116.57	2
14					R	175.12	157.11	158.09	88.06	1

Sample 2 › CL_010.raw › Feature Id: 10153, m/z: 600.8314, RT: 57.11, CV:-60.0, z: 4, -10lgP: 72.80

Scan 28133, m/z=600.8304, z=4, RT=57.03, CV=-60.0, Length=19, -10lgP=72.80, ppm=1.9 1/1

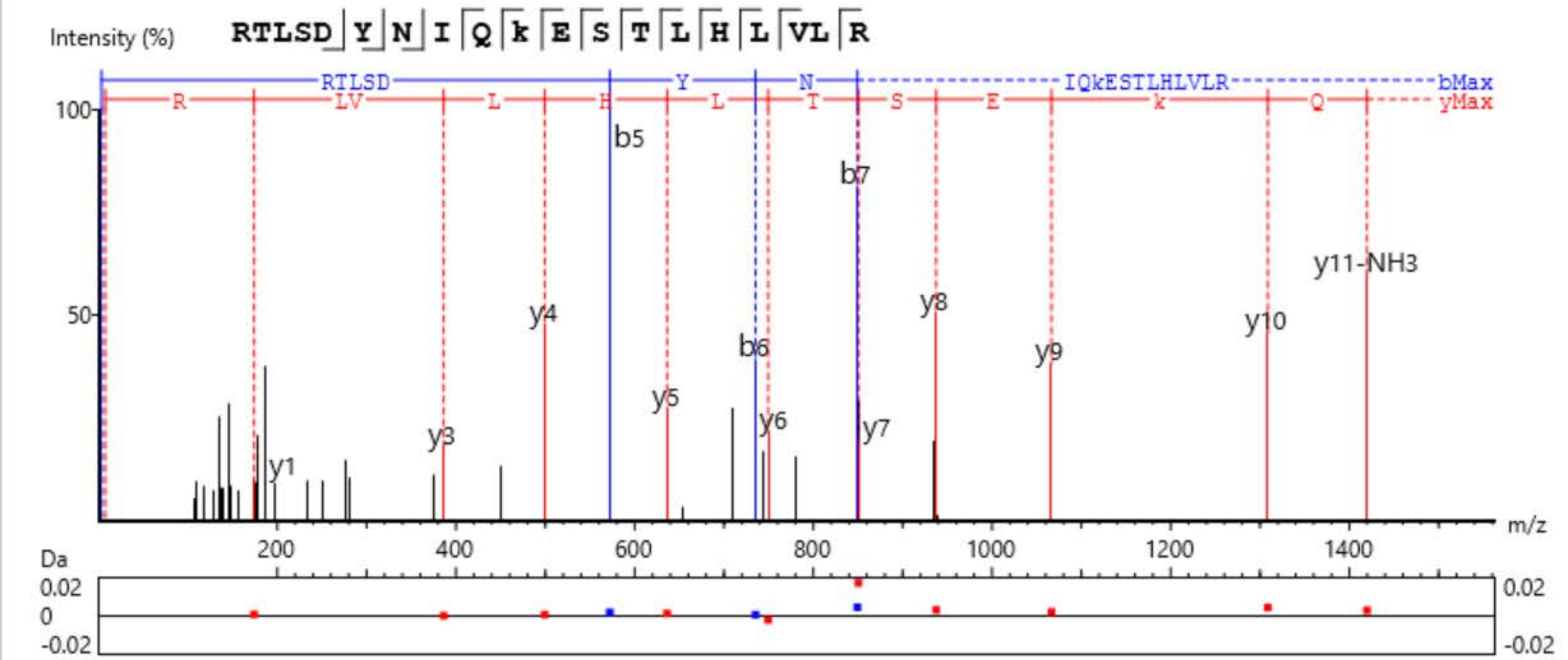

alignment error map

Ion Match Survey

#	b	b-H2O	b-NH3	b(2+)	Seq	y	y-H2O	y-NH3	y(2+)	#
1	157.11	139.10	140.08	79.05	R					19
2	258.16	240.15	241.13	129.58	T	2244.20	2226.19	2227.17	1122.60	18
3	371.24	353.23	354.21	186.12	L	2143.15	2125.14	2126.12	1072.08	17
4	458.27	440.26	441.25	229.64	S	2030.07	2012.06	2013.04	1015.53	16
5	573.30	555.29	556.27	287.15	D	1943.03	1925.02	1926.01	972.02	15
6	736.36	718.35	719.34	368.68	Y	1828.01	1810.00	1810.98	914.50	14
7	850.40	832.40	833.38	425.70	N	1664.94	1646.93	1647.92	832.97	13
8	963.49	945.48	946.46	482.24	I	1550.90	1532.89	1533.87	775.95	12
9	1091.55	1073.54	1074.52	546.27	Q	1437.82	1419.81	1420.79	719.41	11
10	1333.69	1315.68	1316.66	667.34	K(+114.04)	1309.75	1291.75	1292.73	655.38	10
11	1462.73	1444.72	1445.70	731.86	E	1067.62	1049.61	1050.59	534.31	9
12	1549.76	1531.75	1532.73	775.38	S	938.58	920.57	921.55	469.79	8
13	1650.81	1632.80	1633.78	825.90	T	851.53	833.54	834.52	426.27	7
14	1763.89	1745.88	1746.87	882.45	L	750.50	732.49	733.47	375.75	6
15	1900.95	1882.94	1883.92	950.98	H	637.41	619.40	620.39	319.21	5
16	2014.04	1996.03	1997.01	1007.52	L	500.36	482.34	483.33	250.68	4
17	2113.10	2095.09	2096.08	1057.05	V	387.27	369.26	370.24	194.14	3
18	2226.19	2208.18	2209.16	1113.59	L	288.20	270.19	271.18	144.60	2
19					R	175.12	157.11	158.09	88.06	1

Sample 11 | CL_09.raw | Feature Id: 0, m/z: 561.8054, RT: 62.65, CV:-60.0, z: 4, -10lgP: 85.57

Scan 29594, m/z=561.8054, z=4, RT=62.65, CV=-60.0, Length=18, -10lgP=85.57, ppm=0.7   1/1

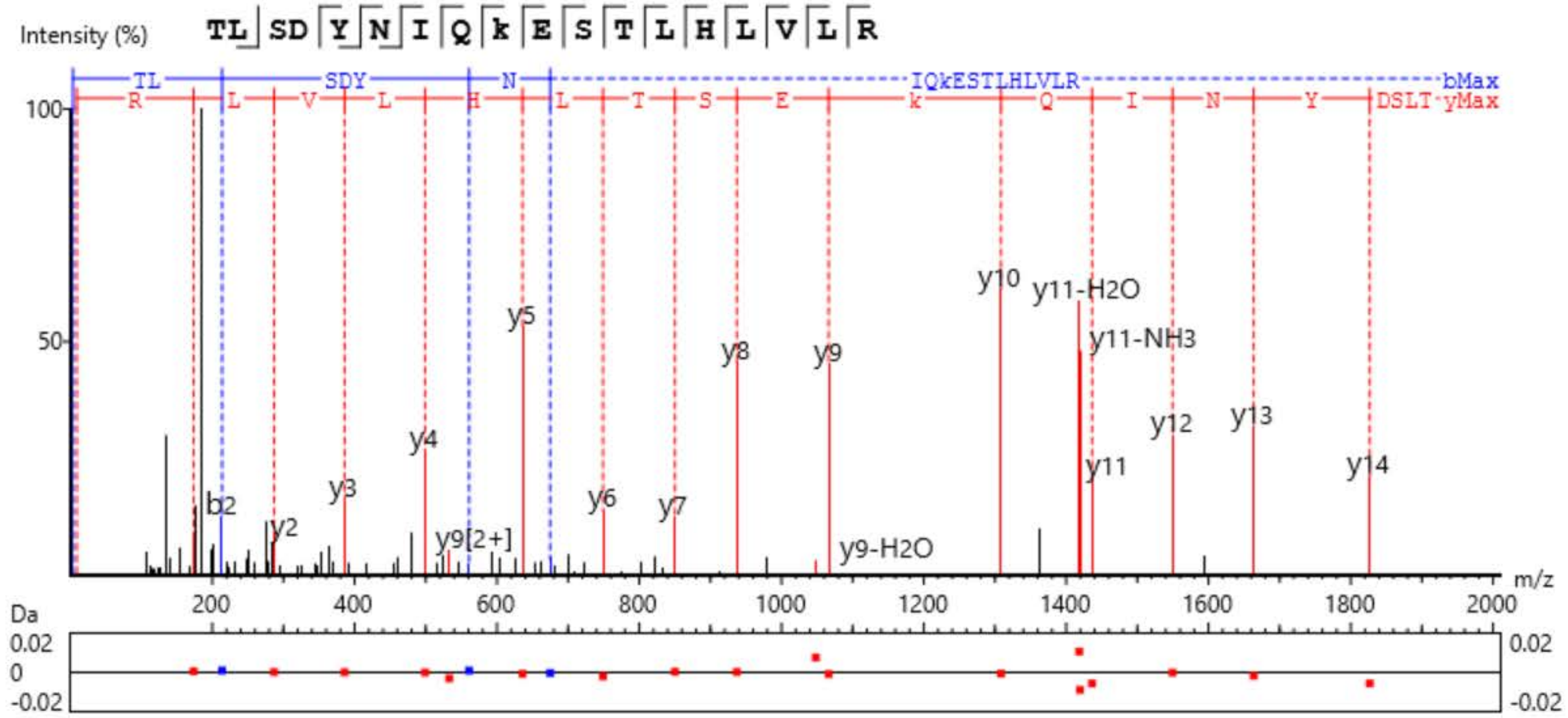

alignment   error map

Ion Match | Survey

#	b	b-H2O	b-NH3	b(2+)	Seq	y	y-H2O	y-NH3	y(2+)	#
1	102.06	84.04	85.03	51.53	T					18
2	215.14	197.13	198.11	108.07	L	2143.15	2125.14	2126.12	1072.08	17
3	302.17	284.16	285.14	151.59	S	2030.07	2012.06	2013.04	1015.53	16
4	417.20	399.19	400.17	209.10	D	1943.03	1925.02	1926.01	972.02	15
5	580.26	562.25	563.23	290.63	Y	1828.01	1810.00	1810.98	914.50	14
6	694.30	676.30	677.28	347.65	N	1664.95	1646.93	1647.92	832.97	13
7	807.39	789.38	790.36	404.19	I	1550.90	1532.89	1533.87	775.95	12
8	935.45	917.44	918.42	468.22	Q	1437.82	1419.80	1420.80	719.41	11
9	1177.59	1159.57	1160.56	589.29	K(+114.04)	1309.76	1291.75	1292.73	655.38	10
10	1306.63	1288.62	1289.60	653.81	E	1067.62	1049.60	1050.59	534.31	9
11	1393.66	1375.65	1376.63	697.33	S	938.58	920.57	921.55	469.79	8
12	1494.71	1476.70	1477.68	747.85	T	851.55	833.54	834.52	426.27	7
13	1607.79	1589.78	1590.76	804.40	L	750.50	732.49	733.47	375.75	6
14	1744.85	1726.84	1727.82	872.93	H	637.42	619.40	620.39	319.21	5
15	1857.93	1839.92	1840.91	929.47	L	500.36	482.34	483.33	250.68	4
16	1957.00	1938.99	1939.98	979.00	V	387.27	369.26	370.24	194.14	3
17	2070.09	2052.08	2053.06	1035.54	L	288.20	270.19	271.18	144.60	2
18					R	175.12	157.11	158.09	88.06	1

Sample 11 〉 CL_09.raw 〉 Feature Id: 6029, m/z: 508.7595, RT: 42.59, CV:-60.0, z: 2, -10lgP: 28.62

Scan 17154, m/z=508.7595, z=2, RT=42.62, CV=-60.0, Length=8, -10lgP=28.62, ppm=-0.2 4/7

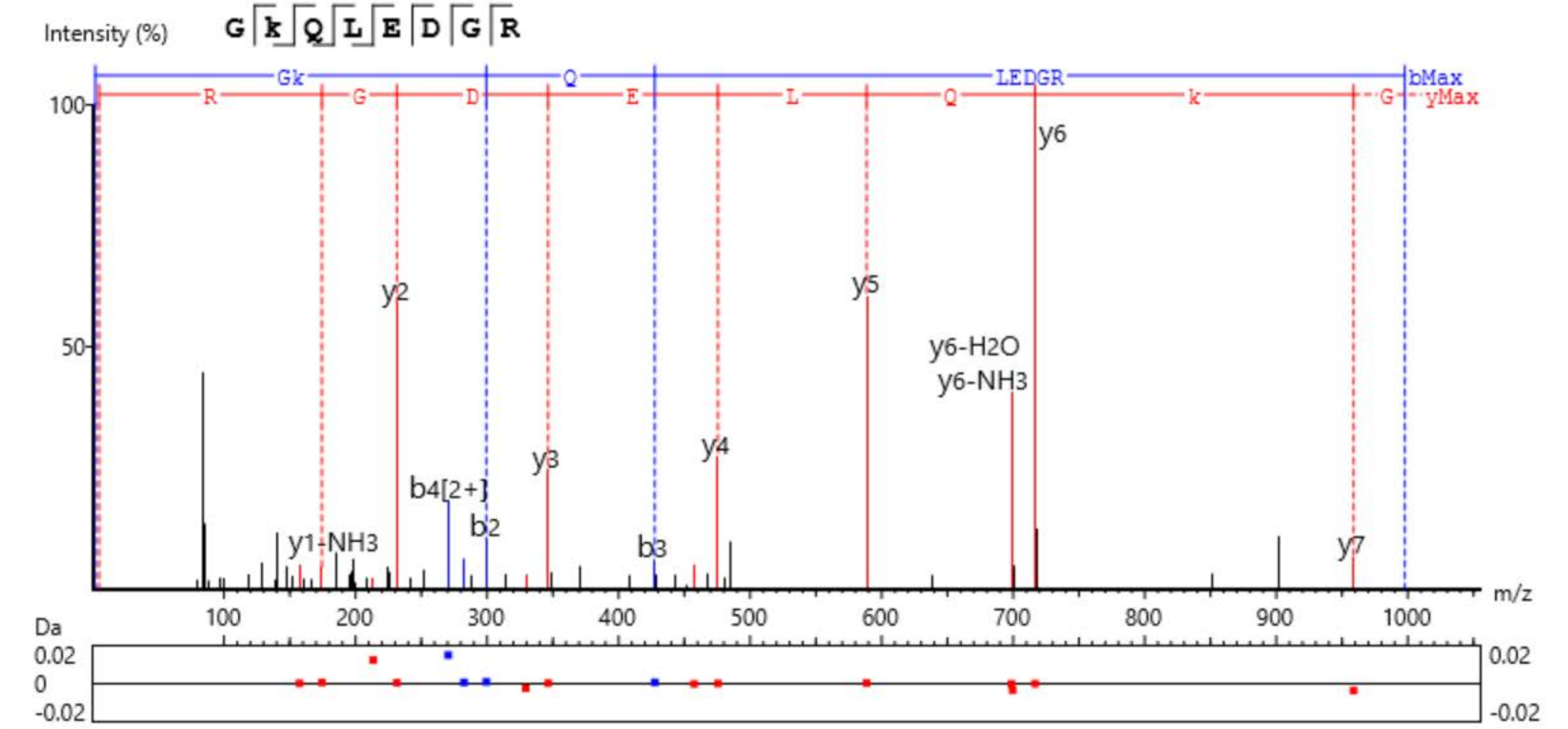

alignment error map

Ion Match | Survey

#	b	b-H2O	b-NH3	b(2+)	Seq	y	y-H2O	y-NH3	y(2+)	#
1	58.03	40.02	41.00	29.51	G					8
2	300.17	282.16	283.14	150.58	K(+114.04)	959.49	941.48	942.46	480.25	7
3	428.23	410.22	411.20	214.61	Q	717.35	699.34	700.33	359.18	6
4	541.31	523.30	524.28	271.14	L	589.29	571.28	572.27	295.15	5
5	670.35	652.34	653.33	335.68	E	476.21	458.20	459.18	238.60	4
6	785.38	767.37	768.35	393.19	D	347.17	329.16	330.14	174.08	3
7	842.40	824.39	825.37	421.70	G	232.14	214.12	215.11	116.57	2
8					R	175.12	157.11	158.09	88.06	1

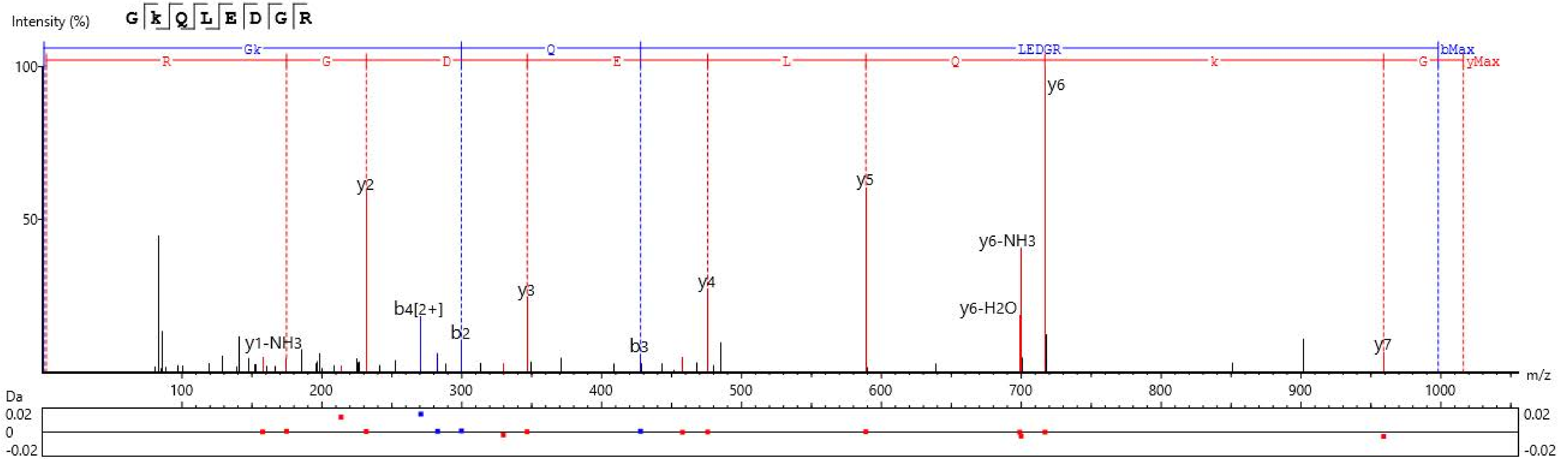

Intensity (%)
G k Q L E D G R
Gk
Q
LEDGR
bMax
R
G
D
E
L
Q
k
G
yMax
100
50
y6
y2
y5
y6-NH3
y4
y3
b4[2+]
y6-H2O
b2
y1-NH3
b3
y7
m/z
100
200
300
400
500
600
700
800
900
1000
Da
0.02
0
-0.02
0.02
-0.02
